# Synthesis of Coumarin and Homoisoflavonoid Derivatives and Analogs: The Search for New Antifungal Agents

**DOI:** 10.3390/ph15060712

**Published:** 2022-06-03

**Authors:** Alana R. Ferreira, Danielle da N. Alves, Ricardo D. de Castro, Yunierkis Perez-Castillo, Damião P. de Sousa

**Affiliations:** 1 Laboratory of Pharmaceutical Chemistry, Department of Pharmaceutical Sciences, Federal University of Paraiba, João Pessoa 58051-900, Paraíba, Brazil; alana.rf@ltf.ufpb.br; 2 Laboratory of Experimental Pharmacology and Cell Culture of the Health Sciences Center, Department Clinical and Social Dentistry, Federal University of Paraiba, João Pessoa 58051-900, Paraíba, Brazil; dnobregaalves@msn.com (D.d.N.A.); rcastro@css.ufpb.br (R.D.d.C.); 3 Bio-Cheminformatics Research Group, Universidad de Las Américas, Quito 170125, Ecuador; yunierkis.perez@udla.edu.ec

**Keywords:** coumarin, medicinal plant, drug, molecular docking, flavonoid, natural products, mechanism of action, antimicrobial, *Candida*

## Abstract

A set of twenty-four synthetic derivatives, with coumarin and homoisoflavonoid cores and structural analogs, were submitted for evaluation of antifungal activity against various species of *Candida.* The broth microdilution test was used to determine the Minimum Inhibitory Concentration (MIC) of the compounds and to verify the possible antifungal action mechanisms. The synthetic derivatives were obtained using various reaction methods, and six new compounds were obtained. The structures of the synthesized products were characterized by FTIR spectroscopy: ^1^H-NMR, ^13^C-NMR, and HRMS. The coumarin derivative **8** presented the best antifungal profile, suggesting that the pentyloxy substituent at the C-7 position of coumarin ring could potentiate the bioactivity. Compound **8** was then evaluated against the biofilm of *C. tropicalis* ATCC 13803, which showed a statistically significant reduction in biofilm at concentrations of 0.268 µmol/mL and 0.067 µmol/mL, when compared to the growth control group. For a better understanding of their antifungal activity, compounds **8** and **21** were submitted to a study of the mode of action on the fungal cell wall and plasma membrane. It was observed that neither compound interacted directly with ergosterol present in the fungal plasma membrane or with the fungal cell wall. This suggests that their bioactivity was due to interaction involving other pharmacological targets. Compound **8** was also subjected to a molecular modeling study, which showed that its antifungal action mechanism occurred mainly through interference in the redox balance of the fungal cell, and by compromising the plasma membrane; not by direct interaction, but by interference in ergosterol synthesis. Another important finding was the antifungal capacity of homoisoflavonoids **23** and **24**. Derivative **23** presented slightly higher antifungal activity, possibly due to the presence of the methoxyl substituent in the *meta* position in ring B.

## 1. Introduction

The incidence and severity of fungal infections, particularly those caused by *Candida* spp., has been increasing worldwide [1]. Candidemia is the most common form of invasive candidiasis in hospital settings, and recent studies in the US indicate that it is the third or fourth most common hospital-acquired infection [2]. *Candida albicans* is still the main species of *Candida* isolated in patients with candidemia. However, in recent years, the percentage of invasive candidiasis caused by non-*albicans* species, resistant to the available antifungals, has been increasing considerably [1,3,4]. The reduced susceptibility of species of *Candida* is related to exposure to and inappropriate use of antifungals [1]. Antifungal resistance is a growing health problem worldwide; considering the limited number of antifungal agents available, it has become necessary to develop new, safer, and more effective molecules [5].

Natural products have become a source of inspiration in new drug discovery and development [6]. The benzopyrone nucleus is found in many natural products and synthetic compound classes (such as coumarins and their derivatives), and constitutes the substructure of other chemical classes, for example flavonoids and homoisoflavonoids. These compounds commonly have diverse pharmacological properties [6,7,8,9].

**Figure 1:**
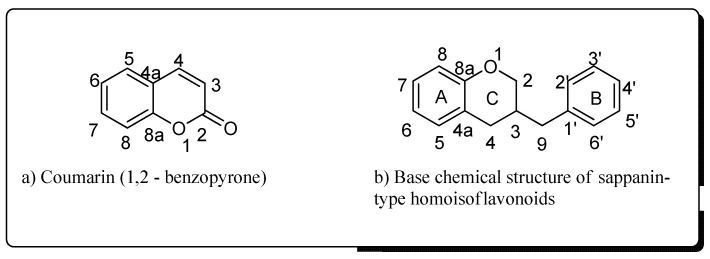
(**a**) Coumarin (1,2-benzopyrone); (**b**) Base chemical structure of sappanin-type homoisoflavonoids.

Coumarins are among the principal secondary metabolites produced by plants. The base skeleton of coumarin is 1,2-benzopyrone (Figure 1a), which is used in design, synthesis, and obtention of many bioactive analogs [10]. Natural and synthetic coumarin derivatives have been associated with a variety of biological activities, such as anti-inflammatory [8], anticancer [11], antioxidant [12], anticoagulant [13], antibacterial [14], antiviral [15] and antifungal activity [16]. Jia et al. 2019 demonstrated that coumarin inhibits growth of *Candida albicans*, reducing strain viability through mechanisms involving fungal cell apoptosis [16].

Homoisoflavonoids (3-benzylidene-4-chromanones) are a small class of compounds structurally related to flavonoids that are rare and uncommon, and have an additional carbon atom in their carbon skeleton [17,18]. They can be subdivided into five subclasses, the most common of which is the sappanin-type (Figure 1b). Homoisoflavonoids, like flavonoids, often have antitumor [19], cardioprotective [20], anti-diabetic [21], antioxidant [8], antiviral, antibacterial and antifungal activity [22], and various studies have confirmed the bioactivity of flavonoids against species of *Candida* [23,24].

Thus, considering the potential bioactivity of coumarins and flavonoids, a series of derivatives and analogs of these chemical classes were prepared to evaluate antifungal activity against species of *Candida*. In order to propose biological targets for these agents, mechanism of action and molecular modeling studies were also performed.

## 2. Results

### 2.1. Chemistry of Compounds ***1***–***24***

**Scheme 1:**
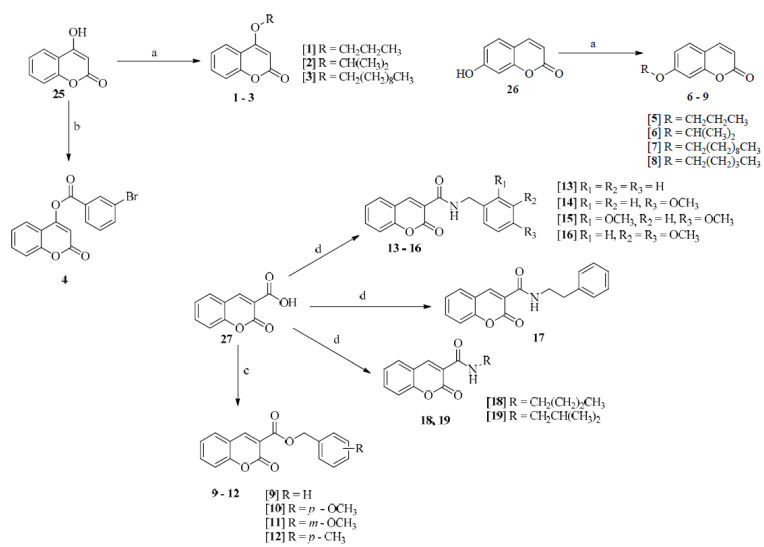
Synthesis of **1–19:** (a) RBr, DMF, K_2_CO_3_, r.t. (b) BrPhCOCl, THF, Et_3_N, 0 °C to r.t. (c) ROH, DCC/DMAP, CH_2_Cl_2_, r.t. (d) RNH_2_, *py*BOP, Et_3_N, DMF, 0 °C to r.t. **25**: 4-hydroxycoumarin, **26**: 7-hydroxycoumarin. **27**: coumarin-3-carboxylic acid.

Derivatives **1**–**8** were prepared through alkylation and acylation reactions of commercial coumarins 4-hydroxycoumarin (**25**) and 7-hydroxycoumarin (**26**), (Scheme 1), according to previously described procedures [25,26]. To obtain the *O*-alkylated derivatives, differing alkyl halides were used, and the yields varied between 24% and 77%. In the acylation reaction, 3-bromo-benzoyl chloride was used, and the yield was 27%. The formation of compound **1** was observed in the ^1^H NMR spectrum, in the C_3_–H singlet with a displacement of δ_H_ 5.64 ppm and aromatic hydrogens of the coumarin skeleton between δ_H_ 7.81 ppm and 7.25 ppm. In the ^13^C NMR spectrum, the shifts of C_2_ at 163.14 ppm and C_4_ at 165.83 ppm were observed. The spectral data for compounds **2** and **3** were similar to those for compound **1**. Unlike compounds **1**–**3**, in spectroscopic analyses of compounds **5**–**8**, two doublets in δ_H_ 7.60 ppm and δ_H_ 6.20 were observed in the ^1^H NMR, respectively, referring to hydrogens C_4_–H and C_3_–H. The ^13^C NMR presented shifts for C-7 at around δ_C_ 162.58–161.42 ppm, and for C-2 between 161.44 and 161.39 ppm.

The ^1^H NMR spectrum of analog **4** showed a singlet at δ_H_ 6.60 ppm referring to C_3_ –H. In the ^13^C NMR, it was possible to observe the shifts of the two carbonyls between δ_C_ 161.31 ppm and 161.38 ppm; it was also possible to observe the shift of C4 at 158.71, and the carbon attached to the bromine atom (C-3′) at δ_C_ 123.25 ppm.

Scheme 1 presents the respective reactions for obtaining esters (**9**–**12**) and amides (**13**–**19**) from coumarin-3 carboxylic acid (**27**) using Steglich esterification (DCC/DMAP) [27] and coupling reactions with *py*BOP [28]. The reaction yields of esters **9**–**12** ranged from 14% to 38%; the formation of the derivatives was evidenced in the ^1^H NMR spectra, in which C_4_–H appeared as a singlet between δ_H_ 9.18 and 8.49 ppm, and in the ^13^C NMR spectra with shifts of the ester C=O (C-9) from δ_C_ 162.92 ppm to 161.62 ppm, and in the lactone C=O (C-2) at δ_C_ 161.49 ppm and 156.74 ppm. The reaction yields of amides **13**–**19** varied between 47% and 73%, and their formation was confirmed in the ^1^H NMR spectra by the singlet with displacement around δ_H_ 8.94 ppm, referring to C_4_–H, and a singlet around δ_H_ 9.20–9.11 ppm, referring to hydrogen –NH. In the ^13^C NMR spectra, the carbonyl carbon shifts between δ_C_ 161 ppm and 160 ppm stood out [29].

**Scheme 2:**
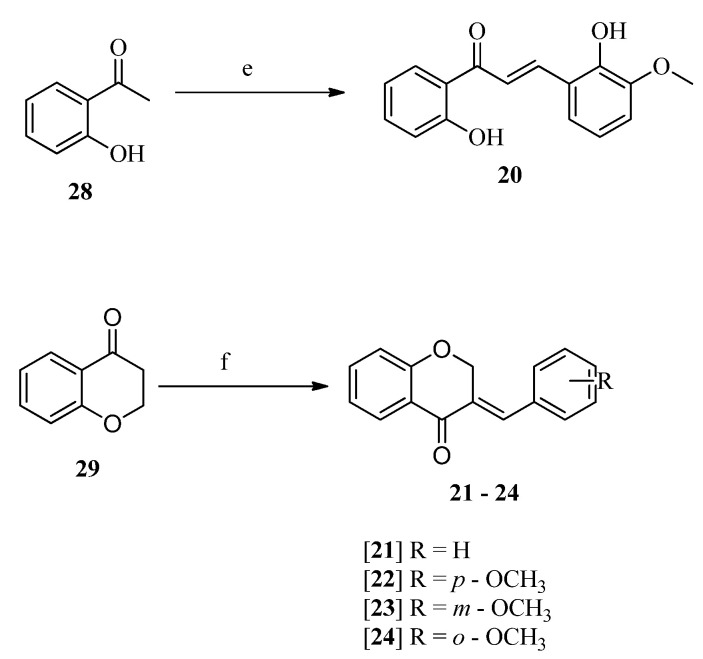
Synthesis of **20–24:** (e) RCHO, NaOH 60%, methanol, r.t (f) RCHO, pyrrolidine, methanol. **28**: 4-chromanone, **29**: 2′-hydroxyacetophenone.

The chalcone **20** was obtained by reaction between 2-hydroxyactophenone (**28**) and 2-hydroxy-3-methoxybenzaldehyde (Scheme 2). In the ^1^H NMR spectrum of **20**, we noted displacement of 2′–*O*-H at δ _H_ 12.47 ppm, and of the hydrogens C-α and C-β to the carbonyl as doublets, respectively δ_H_ 7.81 (d, *J* = 15.6 Hz) and 8.09 (d, *J* = 15.6 Hz). In the ^13^C NMR spectrum, the carbonyl carbon shift was observed at δ_C_ 193.89 ppm, and those of C-α and C-β at around δ_C_ 120.05 ppm and 140.17 ppm, respectively [30].

Homoisoflavonoids **21**–**24**, Scheme 2, were prepared by reactions between 4-chromanone (**29**) and differing aldehydes as catalyzed by pyrrolidine [17]. The yields ranged between 15% and 76%. Structural confirmation involved analyzing ^1^H NMR spectrums, in which a singlet in δ_H_ 7.88 ppm referred to the olefinic hydrogen (C_9_–H) due to the nearby carbonyl group. The doublet in δ_H_ 5.35 (d; *J* = 1.9 Hz; 2H, H-2) corresponds to the hydrogens of C-2, due to the proximity of the phenyl ring. Aromatic hydrogens were noted between δ_H_ 8.03 ppm and 6.95 ppm. In the ^13^C NMR spectra, C-4 shifts around δ_C_ 182.31 ppm, C-9 around δ_C_ 137.56, C-3 around δ_C_ 134.50 ppm, and C-2 around δ_C_ 67.7 ppm were observed [18]. Compounds **3**, **4**, **10**, **11**, **12**, and **16** have not been published in the literature and their structures were confirmed by high resolution mass spectrometry; the spectra are provided in the Supplementary Material.

### 2.2. Antifungal Activity of Compounds ***1***–***24***

In this study, compounds **1**–**24** were tested against strains of *Candida*: *C. albicans* (ATCC 90028), *C. albicans* (ATCC 60193) *C. tropicalis* (ATCC 13803), *C. krusei* (ATCC 6258), *C. parapsilosis* (ATCC 22019) and *C. glabrata* (ATCC 90030). These are the most important pathogens of the genus *Candida* that can cause human diseases, and the main ones involved in invasive infections [1,2]. The bioactivity of the compounds was determined from minimum inhibitory concentration (MIC) values and classified according to Alves et al. 2021 [31], into the following categories: (a) very strong bioactivity (MIC < 3.515 µg/mL); (b) strong bioactivity (MIC between 3.515 and 25 µg/mL); (c) moderate bioactivity (MIC between 26 and 100 µg/mL); (d) weak bioactivity (MIC from 101 to 500 µg/mL); (e) very weak bioactivity (MIC in the range of 501–2000 µg/mL). Table 1 shows the MIC values of all compounds and Table 2 shows the minimum fungicidal concentration (MFC) values and the MFC/MIC ratio for all tested derivatives, through which it is possible to analyze the fungicidal/fungistatic capacity of the respective derivatives.

Compounds **1**, **3**, **5**, **6**, **8**, **16**, **18** and **20**–**24** were bioactive against at least one of the tested strains of *Candida*. Derivative **8**, obtained from 7-hydroxycoumarin, presented the best antifungal profile with a strong activity (MIC of 0.067 µmol/mL) against *C. albicans* (ATCC 90028) and *C. tropicalis* (ATCC 13809), moderate activity (MIC of 0.269 µmol/mL) against *C. krusei* (ATCC 6258), and a weak activity (MIC between 1.07 µmol/mL and 2.15 µmol/mL) against *C. albicans* (ATCC 60193), *C. parapsilosis* (ATCC 22019) and *C. glabrata* (ATCC 90030). Further, according to its MFC values and the MFC/MIC ratio, derivative **8** also exhibited fungicidal capacity against *C. albicans* (ATCC 90028), *C. tropicalis* (ATCC 13809) and *C. krusei* (ATCC 6258). As it showed better antifungal activity in the initial screening, compound **8** was also evaluated for its ability to inhibit the biofilm of *C. tropicalis* ATCC 13803.

Another important finding was the bioactivity of homoisoflavonoid derivatives **21**–**24**. The analogs **23** and **24** presented moderate bioactivity against two or three strains tested.

Further, in order to obtain a better understanding of the antifungal mechanism of the action, compounds **8** and **21** were submitted to tests verifying their mode of action on the fungal cell wall and membrane. Compound **8** was also evaluated in a molecular modeling study.

#### 2.2.1. Verification of the Mode of Action on the Fungal Cell Wall and Membrane

Compounds **9** and **21** were submitted to mechanism of action tests against *C. albicans* ATCC 90028 using a microdilution technique to determine the MIC in the presence of ergosterol and sorbitol.

Ergosterol is one of the main components of the yeast cell membrane, that functions to modulate membrane fluidity, and sorbitol is an osmotic protector that acts by inhibiting changes in the fungal cell wall. To show that the antifungal activity of compounds **8** and **21** resulted from direct interaction with membrane ergosterol or the fungal cell wall, the supply of ergosterol or sorbitol to the culture medium must promote an increase in the MIC of the molecules, because in the presence of exogenous ergosterol, a higher concentration of the compound is required to reach the plasma membrane ergosterol; in the presence of exogenous sorbitol, fungal cells have the osmotic support that allows their growth [32,33].

As reported in Table 3 and Table 4, there was no change in the minimum inhibitory concentration values (MIC) for compounds **8** and **21** when subjected to microdilution tests in the presence of ergosterol and sorbitol. Thus, no direct interaction with plasma membrane ergosterol or with the fungal cell wall was evidenced.

#### 2.2.2. Evaluation of the Antimicrobial Activity of Compound **8** on the Reduction of Fungal Biofilm

**Figure 2:**
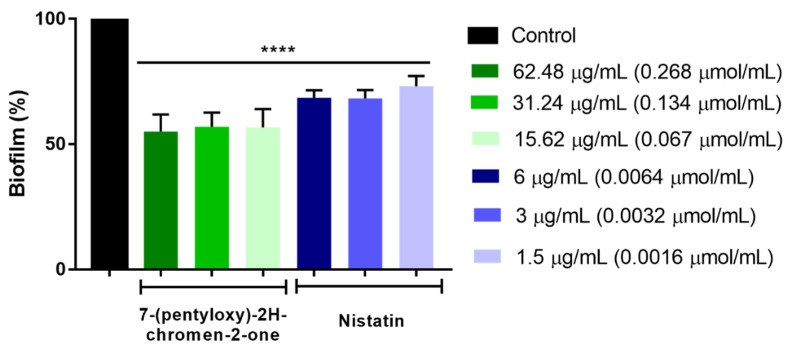
Inhibitory effect (mean, standard deviation) of 7-(pentyloxy)-2H-chromen-2-one (**8**) and nystatin on *Candida* uni-species biofilm. Results presented as mean ± SD of three independent experiments (One-way ANOVA with Tukey post-test, **** *p* < 0.0001).

Figure 2 shows the results of the inhibitory effect of compound **8** and nystatin on *C. tropicalis* biofilm. The test was performed as described in the Section 4. The strain for the assay, *C. tropicalis* strain ATCC 13803, was chosen after preliminary screening of the strains used in the experiment to define the Minimum Inhibitory Concentration.

For compound **8**, there was a reduction of 73% to 68% between the concentrations 0.268 µmol/mL and 0.067 µmol/mL, respectively. For nystatin there was a reduction of 56% to 55% between concentrations of 0.0065 µmol/mL and 0.0016 µmol/mL, respectively. There was a statistical difference when comparing the three concentrations of **8** with the three concentrations of the positive control (nistatin), *p* < 0.0001. There was a statistically significant difference when comparing the growth control with all groups tested, *p* < 0.0001.

### 2.3. Molecular Modeling

Of the series of compounds evaluated, **8** exhibited the best antifungal profile against all the strains tested. Therefore, it was subjected to a molecular modeling study.

Given that the employed modeling workflow included computationally intensive techniques, such as molecular dynamics simulations, the modeling studies focused on compound **8** that had the best antifungal activity. Table 5 lists the potential targets of compound **8** in *C. albicans* using the protocol described in the methods section. The information in the table includes the uniprot accession codes, the ids used for each target during this research and a functional description of each target. *Candida albicans* was selected for the modeling studies because it is the model organism for investigating fungal pathogens and the most studied species of the genus *Candida*. Notably, this set of potential targets is enriched with proteins related to the reductase and dehydrogenase activities. Compound **8** was docked to all proteins listed in Table 5 following the procedure described in the methods section. The results of the docking calculations are provided as supporting information in Table S1 (Supplementary Materials).

According to the molecular docking results, more than one binding pose has a consensus score greater than 1 for all targets except ALD5 and UGA2. This leads to 25 potential ligand-receptor complexes to analyze. The visual inspection of the predicted complexes shows the ligand inside the cavities, complementing with the receptor shape and making favorable ligand-receptor interactions. Furthermore, the best docking scores are obtained for ALD5 and HST2, while the lowest (worst) values are predicted for FBA1 and ARA1.

During the analysis of the molecular docking results, it must be considered that this type of algorithm simplifies or neglects many factors related to molecular interactions. These approximations are necessary for making docking algorithms fast enough to process large databases of molecules in a short period of time in a virtual screening scenario. In consequence, although successful in ranking chemical compounds according to their probabilities of binding one target, the docking scores are not good estimators of the free energy of binding of a ligand to its receptor. To better describe the predicted compound **8**-target complexes, these were subjected to MD simulations and the free energies of binding were predicted with the more accurate MM-PBSA method. Similar approaches employing MD-based tools for the refining of molecular docking predictions have been previously described in the literature. This approach led to 500 ns of MD simulations across all the 25 docking predicted complexes [34,35,36].

**Figure 3:**
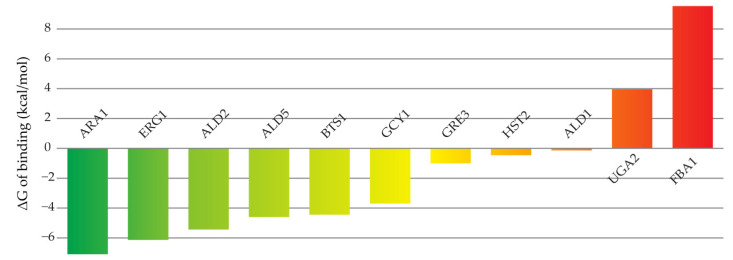
Predicted free energies of binding of compound **8** to its potential targets.

The detailed results of the MM-PBSA calculations are provided as Supporting Information in Table S2 (Supplementary Materials) and summarized in Figure 3. Only the ligand pose having the lowest (best) free energy of binding to each target is presented in the figure. The results of the MM-PBSA calculations show that the most probable targets of compound **8** in *C. albicans* are ARA1, ERG1 and ALD2. Among the top six ranked targets, four (ARA1, ALD2, ALD5 and GCY1) are annotated with the dehydrogenase and reductase activities, thus probably related to the maintaining of the redox balance in the cell. The remaining proteins in this set of six proteins are ERG1 and BTS1, ranked at positions 2 and 5, respectively.

Previous investigations have linked the antifungal activity of coumarin derivatives with the impairing of the redox balance in the cell and with the inhibition of the ergosterol pathway [16,37,38]. Our results are consistent with these previous reports, because, as previously pointed out, four targets related to the redox balance in the cell were identified among the top six candidate targets. In addition, the second most likely target of compound **8** was ERG1 that is part of the ergosterol synthetic pathway. Based on these observations, we further examined in detail the predicted binding modes of the ligand to ERG1, ARA1 and ALD5. The selection of ALD5 over ALD2 for more in depth structural analyses was justified because ARA1, ALD2 and GCY1 share the same folding and highly similar overall orientations of the ligand within their binding cavities.

**Figure 4:**
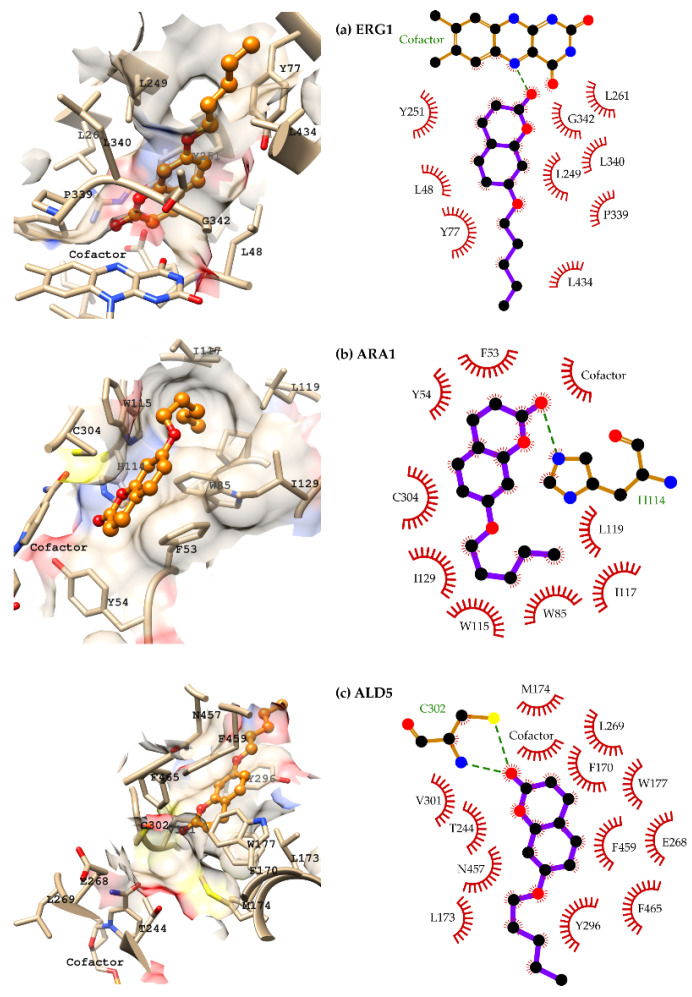
Predicted binding modes and diagrams of ligand-receptor interactions for the proposed complexes compound **8** with ERG1 (**a**), ARA1 (**b**) and ALD5 (**c**). On the left, the ligand is depicted in orange balls and sticks, and the receptors in tan. Oxygen atoms are colored red, nitrogen blue, and sulfur yellow. For the interactions diagrams (right), all atoms are only represented for residues hydrogen bonding the compound.

In Figure 4 are represented the predicted binding pose of compound **8** to ERG1, ARA1 and ALD5. The represented ligand conformation in each complex is the centroid of the largest cluster resulting from clustering the 100 MD snapshots employed for the MM-PBSA calculations. Only residues interacting with the ligand in at least 40% of the analyzed snapshots are labelled in the figure. The pictures showing the complexes’ structure were generated with UCSF Chimera [39], the interaction diagrams were obtained with LigPlot+ [40] and the frequencies of ligand-receptor interactions were analyzed with the Chimera interface of Cytoscape [41].

A feature common to all predicted complexes is that the coumarin ring orientates toward the cofactor. Furthermore, the carbonyl oxygen accepts hydrogen bonds from the receptor in all cases. In ERG1, this hydrogen bond is accepted from the flavin system. In addition, the coumarin ring is located in a hydrophobic pocket of ERG1 stacking in front of Y251 and interacting with L48, L249, L261, P339, L340 and G342. The pentyl tail of compound **8** is predicted to mainly interact with the side chains of Y77 and L434. Likewise, in the predicted complex with ARA1, the coumarin ring of the compound is located in a hydrophobic region lined by the cofactor, F53, Y54, W85, H114, W115, I129 and C304. On the other side, the pentyl moiety is accommodated in a superficial small groove shaped by the side chains of W85, P86, W115, I117 and L119.

Finally, the compound **8**-ALD5 complex shows higher flexibility than the former with the ligand occupying two main regions. In some MD snapshots, the ligand is observed close to the cofactor, completely buried in the cavity, and directly interacting with the cofactor and M174, T244, E368 and L269. The other majority group of ligand conformations adopt the binding mode represented in Figure 4, where the ligand hydrogen bonds C302 and the coumarin ring is stacked between F170 and F459 while it interacts with L173, W177, V301 and F465. The pentyl ring in this ligand orientation is mainly exposed to the solvent flanked by the active site entrance residues Y296 and N457.

In addition to the energetic stability of the predicted complexes discussed above, their conformational stability was also analyzed. This was monitored by computing the RMSD of the ligand in each complex relative to its initial docked conformation during the production runs. The RMSD plots for all complexes are provided as Supplementary Materials in Figures S1–S25 (Supplementary Materials). From this analysis, it can be inferred that the ligand remains stable in all complexes during MD simulations, with the RMSD values relative to the starting conformations lower than 2 Å, or close to this value, in all trajectories. Furthermore, the variations in the RMSD values suggest that the main objective of the performed MD simulations, that was to obtain an ensemble of complex conformations for free energy calculations, was achieved.

The conservation of the amino acids from the most probable targets of compound **8** in *C. albicans* interacting with the ligand in *C. tropicalis* and *C. krusei* was also analyzed. These analyzes showed that all interacting residues were conserved in ERG1 across the three species, while no interacting residues of ALD2, ALD5 and GCY1 were mutated in *C. tropicalis*. A few mutations were observed on the later three proteins of *C. krusei* relative to *C. albicans*: I51V and N80S in ALD2, N85S, C286V in GCY1 and Y296F in ALD5. The less conserved protein across the three species was ARA1 that harvest the mutations F53Y and I129V in *C. tropicalis*, and F53Y, I117R, L119F, I129V and C304R in *C. krusei*. The fact that *C. krusei* ARA1 was the most divergent protein among the identified potential targets of compound **8** in *C. albicans*, combined with the four-fold reduction on the activity of the chemical against the first species, led us to hypothesize that ARA1 could have a relevant role on the antifungal mechanism of action of the compound. Despite this, ARA1 was not proposed as a target for antifungal compounds; based on the obtained results we consider that additional experiments focusing on the evaluation of its potential as antifungal target should be performed.

### 2.4. ADMET Predictions

**Figure 5:**
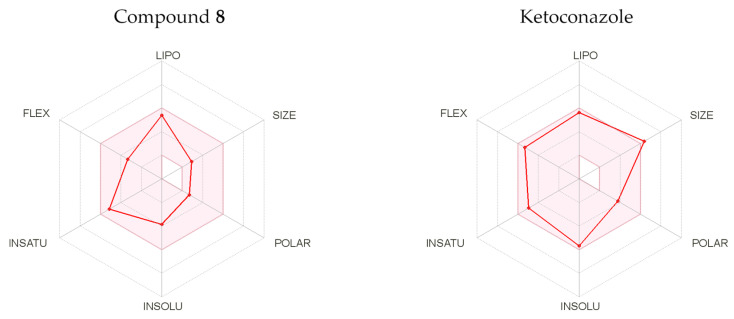
Bioavailability radars for compound **8** and the control ketoconazole.

The ADMET predictions were performed as described in the Section 4. The predicted ADMET properties of compound **8** and ketoconazole are presented in Table 6 and their oral bioavailability radars, as provided by the SwissADME server, are shown in Figure 5. As observed from Table 6 and Figure 5, both compounds fall into the suitable physicochemical space for oral bioavailability (colored zone). In contrast to ketoconazole, that has physicochemical properties close to the properties’ limits, compound **8** can still be modified to improve its bioactivity without falling outside the favorable oral bioavailability region. According to these results, the future optimization of compound **8** can include increases in the number of rotatable bonds, molecular weight, polarity, and insolubility. On the other hand, any newly derivative of compound **8** should not increase lipophilicity nor unsaturation to keep new compounds within the favorable oral bioavailability region.

For ADMET properties, the absorption endpoints are very similar for compound **8** and ketoconazole, with the first predicted with slightly better gastrointestinal absorption than ketoconazole. Distribution metrics show that compound **8** has a higher chance of crossing the blood-brain barrier than ketoconazole and this property must be improved in further optimization campaigns. On the other hand, the predicted cytochrome P450 metabolism profile is more favorable for compound **8** as it is only predicted to inhibit two of the evaluated enzyme isoforms, in contrast to the four variants of the protein inhibited by ketoconazole. Finally, the toxicity of compound **8** compared to ketoconazole shows mixed results. The studied compound is predicted to be non-hepatotoxic, while ketoconazole is predicted to be toxic for the liver, and a similar scenario is predicted for hERG II inhibition.

## 3. Discussion

In this study, twenty-four compounds, containing either benzopyrone or chromanone nuclei or structural analogs, were synthesized and (along with their starting materials) subjected to antifungal evaluation. The antimicrobial potential of this potentially bioactive collection of compounds was investigated, and despite the structural diversity of this synthetic derivative group, the compounds are related through their base nuclei: coumarin (1,2-benzopyrone) of compounds **1**–**19** and the 4-chromanone (2,3-dihydro-1-benzopyran-4-one) of analogs **21**–**24**. In addition, due to structural similarity with the other compounds, chalcone **20** was also used for comparative study. Small structural modifications can result in large differences in the chemistry and bioactivity of these compounds [42].

Analyzing the results of antifungal activity, see Table 1 and Table 2, of all molecules that present a 1,2-benzopyrone (coumarin) skeleton in their structure (compounds **1**–**19**), the C_7_ -*O*-alkylated coumarin derivatives **5**, **6**, and **8** showed the better antifungal activity. The substitutions performed at the C-3 and C-4 positions of coumarin did not lead to a molecule with potent antifungal bioactivity against different strains of *Candida*; most of these derivatives were inactive against all tested strains or presented weak bioactivity.

It is worth noting that compound **3** (4-(decyloxy)-*2h*-chromen-2-one), a new compound in the literature, exhibited moderate bioactivity against *C. krusei* ATCC 6258 with a MIC of 0.103 µmol/mL with fungicidal capacity, according to MFC/MIC value (MFC = 0.206 µmol/mL). When compared to the rest of the molecules evaluated, compound **3** showed the best antifungal activity against *C. krusei* ATCC 6258. This finding is interesting because *Candida krusei* is among the non-*albicans* species most frequently involved in mild and severe *Candida* infections, which have been associated with the increased resistance of non-*albicans* species to the available antifungals [43]. Obviously, further study is needed for a better understanding of the influence of the substitution positions in coumarin, as well defining ideal alkyl chain lengths for better activity against species of *Candida.*

Comparing the bioactivity results (MIC and MFC values) of **5**, **6**, **8** with that of **26** (their starting material), it was observed that the insertion of modifications at the C-7 position of coumarin led to a compound with more potent antifungal activity (compound **8**); in this case, it can be suggested that the size of the alkyl group substituted in the C-7 position is important for bioactivity, considering that with the increase in the chain length from three to five carbons, compound **8** (7-(pentyloxy)-*2H*-chromen-2-one) exhibited a better antifungal profile than its analogues **5** and **6** (three carbon side chains). Compound **9** (with a ten-carbon side chain) was inactive against all strains in the test. Pan et al. 2018 made similar observations in their antifungal studies against phytopathogenic fungi with umbelliferone derivatives [44]. Chu et al. 2017 indicated that the increase in lipophilicity arising from a methoxyl at the C-7 position of coumarin can influence the antimicrobial bioactivity of herniarin derivatives [45].

The chalcone **20** showed moderate activity (MIC = MFC = 0.231 µmol/mL) against *C. tropicalis* ATCC 13803, with fungicidal capacity against this same strain, according to the MFC/MIC value. The antimicrobial activity of flavonoids and their precursors, as well as the ability to inhibit the growth of *Candida* spp. has been associated with A and B ring substituents, especially by presence, number, and position of hydroxyl groups, especially in the B ring of their chemical structures [46,47]. Considering their influence on aromatic ring resonance, the 2′–OH and 3′–OCH_3_ groups were likely important for the bioactivity exhibited by compound **20** [48].

The homoisoflavonoids **21**–**24** were the next most active compounds, after compound **8**. This class of natural products presents many biological properties, such as cytotoxic [49], antioxidant, anti-inflammatory [50], antibacterial [51], antiviral [52] and antifungal activity [53]. For the antifungal activity observed in this study, we evaluated the influence of the methoxyl substituent present in the B ring of derivatives **22**, **23**, and **24**. Comparing derivatives **21** (non-methoxylated) and **23**, it was observed that the –OCH_3_ group in the *meta* position of the B ring conferred a slightly higher antifungal activity to derivative **23**, which presented moderate bioactivity according to the MIC value (MIC = 0.234 µmol/mL) and fungicidal capacity according to MFC/MIC ratio (MFC = 0.234 µmol/mL) against *C. albicans* 90028, *C. tropicalis* ATCC 13803 and *C. krusei* ATCC 6258, unlike **21** that showed weak bioactivity (MIC = MFC = 1.06 µmol/mL) against *C. tropicalis* ATCC 13803 and was inactive against *C. albicans* ATCC 60193, *C. parapsilosis* ATCC 22019 and *C. glabrata* ATCC 90030. Derivatives **22** (*p* -OCH_3)_ and **24** (*o*-OCH_3)_, as compared to **23**, did not present better antifungal activity, as derivative **22** was inactive against most of the tested strains and derivative **24** presented weak activity (MIC = 1.87 µmol/mL, MFC = 3.75 µmol/mL) against *C. krusei* ATCC 6258. Our findings suggest that the presence of the methoxyl group at the *meta* position of the B ring contributed to the better antifungal bioactivity found for derivative **23**. Das et al. 2015 [51] evaluated analogues **22**, **23**, and **24**, among other 3-benzylidene-4-chromanones derivatives, for anti-mycobacterial activity (*Mycobacterium tuberculosis*); to obtain potent anti-mycobacterial agents, the authors suggested the importance of inserting small substituent groups into the aromatic rings of the analog series under study. They also related the better bioactivity with the position of aromatic substituents in the B ring. Thus, molecules containing this substituent in the *meta* position*,* such as derivative **23** (*m*-OCH_3_), presented the most potent anti-mycobacterial activity, followed by *ortho*-substituted and later *para*-substituted derivatives (*meta* > *ortho* > *para*).

Of the set of molecules evaluated, compound **8** showed the best antifungal profile, followed by homoisoflavonoids **23** and **24**. The antifungal activity of **8** was then investigated against the biofilm of *C. tropicalis* ATCC 13803 (Figure 3). The ability of *Candida* spp. to form biofilms is an important virulence factor contributing to drug resistance in the clinic; biofilm formation on implanted medical devices represents a major source of long-term candidaemia [1,2]. The assay showed that for the three tested concentrations of compound **8**, 0.268 µmol/mL, 0.134 µmol/mL, and 0.067 µmol/mL, there were statistically significant differences in relation to the growth control group, observing, respectively, a reduction of biofilm of approximately 73% in *p* < 0.0001, 68% at *p* < 0.0001, and 68% at *p* < 0.0001. Likewise, nystatin expressed a statistically significant difference in relation to growth control at the three concentrations tested 0.0064 µmol/mL, 0.0032 µmol/mL, and 0.0016 µmol/mL, with the respective reductions 56%, 56% and 55%, *p* < 0.0001.

To better understand the antifungal mechanism of action of the derivatives, compounds **8** and **21** (which has the same basic structural core as analogues **23** and **24**) were submitted to a test of verification of the mode of action on the fungal cell wall and plasma membrane, using the strain *C. albicans* 90028. The test results (Table 3 and Table 4) showed that there was no direct interaction between the molecules and ergosterol, component of the fungal cell membrane, or sorbitol, osmotic protector of the cell wall. This suggests that the antifungal mechanism of action found for compounds **8** and **21** involves interaction with other pharmacological targets.

Antifungal activity of compound **8** was further investigated through a molecular modeling study. The proposed binding modes of compound **8** to its most probable targets was consistent with the observed antifungal activities. Compound **8** contains a coumarin nucleus while the next most active compounds (**21** and **23**) are chromone derivatives. For all three molecular targets analyzed above, the carbonyl oxygen of the coumarin ring is predicted to hydrogen bond either a cofactor or the receptor. The positional change of this oxygen in the chromone moiety would interfere with these hydrogen bonds predicted for compound **8**, making the complexes with compounds **21** and **23** less energetically stable. We consider this a plausible hypothesis as, in all the three cases, there is enough space in the binding cavities to accommodate the substituents of compounds **21** and **23**.

Taken together, the results presented herein suggest a multi-target antifungal mechanism of action of compound **8**. This is predicted to interfere with two processes critical for survival of *C. albicans*, the synthesis of the cell membrane and the redox balance in the cell. The presented hypothesis for the mechanism of action of the compound is supported by previous experimental evidence showing the coumarin derivatives interfere with the synthesis of ergosterol and induce oxidative stress in *C. albicans* [16,38]. The presented research could help to guide future experiments focusing on the experimental identification of the targets of compound **8** in *C. albicans* and in the optimization of its antifungal activity.

Overall, the ADMET properties of compound **8** show a profile like that of the reference antifungal drug ketoconazole. Considering that the chemical under investigation is a hit compound, the predictions presented in this section are a valuable tool for its further development. In consequence, any future lead candidate must have improved ADMET properties relative to compound **8** and the improvement of their ADMET properties must be considered along with the optimization of the antifungal activity.

## 4. Materials and Methods

### 4.1. Chemistry

Structural confirmation of the prepared compounds was carried out by infrared spectroscopy analysis in an Agilent technologies Cary 630 FTIR instrument in a spectral range in the region of 4000–400 cm^−1^, ^1^H NMR and ^13^C NMR; in Ascend^TM^–Bruker spectrometers operating at 400 MHz (^1^H) and 100 MHz (^13^C) and the Varian-NMR-System operating at 500 MHz (^1^H) and 125 MHz for (^13^C). The High Resolution Mass Spectrometry analysis was performed on a TOF/TOF Ultraflex II mass spectrometer equipped with a high performance solid state laser (λ = 355) and reflector. The system was operated using FlexControl 2.4 (Bruker Daltonics GmbsH, Bremen, Germany) and a QqToF Impact HD mass spectrometer (Bruker Daltonics GmbH, Bremen, Germany) equipped with an ionization source ESI coupled to the Agilent 1290 Infinity II UHPLC chromatographic system (Agilent Technologies, Agilent 1290 Infinity II LC, Waldbronm, Germany)) consisting of a binary pump (G7120A–High speed Pump), auto-injector, column compartment (G7129B–1290 Vialsampler) and variable wavelength ultraviolet light (G7114B–model 1260 Infinity II–VWD). In this case, data acquisition and processing were performed using Data Analysis^®^ software (Bruker Daltonics GmbH, Bremen, Germany). Melting points were determined in a Microquímica apparatus (Microquímica equipamentos LTDA, Model MQAPF 302, Serial No.: 403/18, Palhoça, Brazil)) with temperature measurement in the 10 °C to 350 °C range. All reactions were monitored by analytical thin layer chromatography.

#### 4.1.1. Methodology for Obtaining Ethers Derived from 4-Hydroxycoumarin (**1**–**3**) and 7-Hydroxycoumarin (**5**–**8**)

Hydroxycoumarin (1.233 mmol) was solubilized in dimethylformamide (5.0 mL). Alkyl halide (1.0 equiv.) and potassium carbonate (K_2_CO_3_, 3 equiv.) were then added. The reactions were kept under stirring at room temperature for 24 h. The reaction mixture was then filtered and poured into chilled distilled water; the precipitate formed was solubilized in dichloromethane for drying with anhydrous sodium sulfate (Na_2_SO_4_) to obtain the products. Compounds **7** and **8** were further purified with silica gel 60 column chromatography using hexane and ethyl acetate as eluents [25].

4-Propoxy-*2H*-chromen-2-one (**1**): Crystalline white solid. Yield: 46% (0.244 mmol; 58 mg). M.P.: 105–106 °C (lit. = 110–111 °C [54]); TLC (6:4 hexane/EtOAc); R*_f_* = 0.74. IR ʋmax (KBr, cm^−1^): 3061 (C-H, sp^2^); 2968 (C-H, sp^3^); 1707 (C=O); 1624, 1606, 1563 (C=C aromatic); 1240, 1179 (C-O). ^1^H NMR (400 MHz, CDCl_3_): δ = 7.81 (dd, *J* = 7.9; 1.5 Hz; 1H, H-5); 7.52 (ddd, *J* = 8.4; 7.2; 1.6 Hz; 1H, H-7); 7.29 (dd; *J* = 8.4; 1.1 Hz; 1H, H-8); 7.25 (ddd, *J* = 7.9; 7.3; 1.1 Hz; 1H, H-6); 5.64 (s, 1H, H-3); 4.07 (t, *J* = 6.4 Hz, 2H, H-1′); 1.92 (sext, *J* = 7.4 Hz; 2H, H-2′); 1.09 (t, *J* = 7.4 Hz, 2H, H-3′). ^13^C NMR (100 MHz, CDCl_3_): δ = 165.83 (C-4); 16.14 (C-2); 153.43 (C-8a); 132.39 (C-7); 123.92 (C-5); 123.10 (C-6); 116.82 (C-8), 115.92 (C-4a); 90.44 (C-3); 70.89 (C-1′); 22.03 (C-2′); 10.54 (C-3′) [54].

4-Isopropoxy-*2H*-chromen-2-one (**2**): White solid. Yield: 24.5% (0.156 mmol; 32 mg). M.P.: 116.4–116.8 °C.; TLC (7:3 hexane/EtOAc); R*_f_* = 0.53. IV ʋmax (KBr, cm^−1^): 3086 (C-H, sp^2^), 2994 (C-H, sp^3^); 1710 (C=O); 1621, 1606, 1561 (C=C aromatic); 1250, 1103 (C-O). ^1^H NMR (400 MHz, CDCl_3_): δ = 7.82 (dd; *J* = 7.9; 1.5 Hz; 1H, H-5); 7.53 (ddd, *J* = 8.4; 7.2; 1.6 Hz; 1H, H-7); 7.31 (dd; *J* = 8.4; 1.1 Hz; 1H, H-8); 7.25 (ddd, *J* = 8.0; 7.3; 1.1 Hz; 1H, H-6); 5.65 (s, 1H, H-3); 4.72 (sept, *J* = 6.0 Hz; 1H, H-1′); 1.47 (d, *J* = 6.0 Hz; 6H, H-2′). ^13^C NMR (100 MHz, CDCl_3_): δ = 164.69 (C-4); 163.42 (C-2); 153.63 (C-8a); 132.40 (C-7); 123.89 (C-5); 123.36 (C-6); 116.87 (C-8); 116.34 (C-4a); 90.78 (C-3); 72.44 (C-1′); 21.60 (C-2′) [55].

4-Decyloxy-*2H*-chromen-2-one (**3**): White solid. Yield: 32% (0.3901 mmol; 118 mg). M.P.: 69.2–70.1 °C.; TLC (7:3 hexane/EtOAc); R*_f_* = 0.74. IR ʋmax (KBr, cm^−1^): 3061 (C-H sp^2^) 2950 (C-H sp^3^); 1714 (C=O); 1624, 1607, 1563 (C=C aromatic); 1238, 1110 (C-O). ^1^H NMR (500 MHz, CDCl_3_) δ: 7.82 (dd, *J* = 7.9; 1.5; 1H, H-5); 7.54 (ddd, *J* = 8.4; 7.2; 1.6 Hz; 1H, H-7); 7.31 (dd; *J* = 8.4; 1.1 Hz; 1H, H-8); 7.27 (ddd, *J* = 7.9; 7.3; 1.1 Hz; 1H, H-6); 5.66 (s, 1H, H-3); 4.12 (t, *J* = 6.5 Hz; 1H, H-1′); 1.90 (m, 2H, H-2′); 1.50 (m, 2H, H-3′); 1.31 (m, 12H, H-4′–H-9′); 0.88 (t, *J* = 6.8 Hz; 1H, H-10′). ^13^C NMR (125 MHz, CDCl_3_) δ: 165.71 (C-4); 163.04 (C-2); 153.31 (C-8a); 132.06 (C-7); 123.72 (C-5); 123.00 (C-6); 116.74 (C-8); 115.84 (C-4a); 90.34 (C-3); 69.43 (C-1′); 31.85 (C-8′); 29.48 (C-2′); 29.47 (C-4′); 29.26 (C-5′); 29.21 (C-6′); 28.45 (C-7′); 25.92 (C-3′); 22.64 (C-9′); 14.08 (C-10′). LC-MS/MS analyze: C_19_H_26_O_3_ calculated theoretical value (M + H^+^) = 303.1962. Found = 303.1964.

7-propoxy-*2H*-chromen-2-one (**5**): Crystalline white solid. Yield: 77% (0.957 mmol). M.P.: 60.7–61 °C (lit.: 67.6 °C [56]); TLC (7:3 hexane/EtOAc); R*_f_* = 0.68. IR ʋmax (KBr, cm^−1^): 3087 (C-H sp^2^); 2965 (C-H, sp^3^); 1726 (C=O); 1619 (C=C), 1511, 1472, 1401 (C=C aromatic); 1231, 1123 (C-O). ^1^H NMR (500 MHz, CDCl_3_): δ 7.60 (d, *J* = 9.5 Hz, 1H, H-4); 7.33 (d, *J* = 8.6 Hz, 1H, H-5), 6.81–6.79 (m, 1H, H-6), 6.76–6.75 (m, 1H, H-8), 6.20 (d, *J* = 9.5 Hz, 1H, H-3) 3.95 (t, *J* = 6.5 Hz, 2H, H-1′), 1.82 (sext, *J* = 7.4 Hz, 2H, H-2′), 1.03 (t, *J* = 7.4 Hz, 3H, H-3′). ^13^C NMR (125 MHz, CDCl_3_): δ 162.50 (C- 7), 161.31 (C-2), 155.84 (C-8a), 143.53 (C-4), 128.79 (C-5), 112.99 (C-6) *, 112.93 (C-3) *, 112.44 (C-4a), 101.41 (C-8), 70.18 (C-1′), 22.41 (C-2′), 10.49 (C-3′) [56]. * interchangeable.

7-isopropoxy-*2H*-chromen-2-one (**6**): Amorphous white solid. Yield: 60.8% (0.751 mmol; 153.4 mg). M.P.: 55.3–56.4 °C (lit.: 49–50 °C [57]); TLC (7:3 hexane/EtOAc); R*_f_* = 0.68. IR ʋmax (KBr, cm^−1^): 3061 (C-H, sp^2^); 2983 (C-H, sp^3^); 1720 (C=O); 1622 (C=C); 1239, 1135 (C-O). ^1^H NMR (500 MHz, CDCl_3_): δ 7.61 (d, *J* = 9.5 Hz, 1H, H-4), 7.33 (d, *J* = 8.4 Hz, 1H, H-5), 6.80–6.75 (m, 2H, H-6, H-8), 6.20 (d, *J* = 9.5 Hz, 1H, H-3), 4.59 (hept, *J* = 6.0 Hz, 1H, H-1′), 1.35 (d, *J* = 6.0 Hz, 6H, H-2′). ^13^C NMR (125 MHz, CDCl_3_): δ 161.42 (C-7) *, 161.35 (C-2) *, 156.01 (C-8a), 143.57 (C-4), 128.85 (C-5), 113.83 (C-6), 112.86 (C-3), 112.31 (C-4a), 102.30 (C-8), 70.78 (C-1′), 21.87 (C-2′) [57]. * Interchangeable.

7-(decyloxy)-*2H*-chromen-2-one (**7**): Amorphous solid, yellow. Yield: 41.9% (0.5168 mmol, 156.3 mg). M.P.: 44.2–45.7 °C. TLC (9:1 hexane/EtOAc); R*_f_* = 0.36. IR ʋmax (KBr, cm^−1^): 3081 (C-H, sp^2^); 2922 (C-H, sp^3^); 1729 (C=O); 1615 (C=C); 1236, 1125 (C-O). ^1^H NMR (500 MHz, CDCl_3_): δ 7.62 (d, *J* = 9.6 Hz, 1H, H-4), 7.35 (d, *J* = 8.5 Hz, 1H, H-5), 6.82 (dd, *J* = 8.5; 2.4 Hz, 1H, H-6), 6.79 (d, *J* = 2.4 Hz, 1H, H-8), 6.23 (d, *J* = 9.5 Hz, 1H, H-3), 4.00 (t, *J* = 6.6 Hz, 1H), 1.84–1.77 (m, 2H, H-2′), 1.48–1.42 (m, 2H, H-3′), 1.33–1.26 (m, 12H, H-4′ à H-9′), 0.87 (t, *J* = 6.9Hz, 3H). ^13^C NMR (125 MHz, CDCl_3_): δ 162.58 (C-7), 161.44 (C-2), 156.06 (C-8a), 143.59 (C-4), 128.86 (C-5), 113.13 (C-6), 113.02 (C-3), 112.48 (C-4a), 101.45 (C-8), 68.81 (C-1′), 32.01 (C-2′), 29.66 (C-3′, C-4′), 29.46 (C-5′), 29.43 (C-6′), 29.10 (C-7′), 26.07 (C-8′), 22.80 (C-9′), 14.24 (C-10′).

7-(pentyloxy)-*2H*-chromen-2-one (**8**)**:** Yellow oil. Yield: 63% (0.783 mmol). TLC (9:1 hexane/EtOAc); R*_f_* = 0.30. IR ʋmax (KBr, cm^−1^): 3082 (C-H, sp^2^), 2932 (C-H, sp^3^); 1728 (C=O); 1613 (C=C); 1509 (C=C aromatic), 1234, 1122 (C-O). ^1^H NMR (500 MHz, CDCl_3_): δ 7.62 (d, *J* = 9.5 Hz, 1H, H-4), 7.35 (d, *J* = 8.6 Hz, 1H, H-5), 6.82 (dd, *J* = 8.6; 2.5 Hz, 1H, H-6), 6.79 (d, *J* = 2.5 Hz, 1H, H-8), 6.22 (d, *J* = 9.5 Hz, 1H, H-3), 4.00 (t, *J* = 6.6 Hz, 2H, H-1′), 1.81 (quint, *J* = 6.6, 2H, H-2′), 1.50–1.33 (m, 4H, H-3′, H-4′), 0.93 (t, *J* = 7.1Hz, 3H, H-5′). ^13^C NMR (125 MHz, CDCl_3_): δ 162.58 (C-7), 161.39 (C-2), 156.06 (C-8a), 143.56 (C-4), 128.81 (C-5), 113.11 (C-6), 113.03 (C-3), 112.50 (C-4a), 101.47 (C-8), 68.80 (C-1′), 28.80 (C-2′), 28.22 (C-3′), 22.53 (C-4′), 14.10 (C-5′).

#### 4.1.2. Methodology for Obtaining 4-Hydroxycoumarin Derivative **4**

4-hydroxycoumarin (1.23 mmol) was dissolved in 5.0 mL of tetrahydrofuran. The solution was stirred in an ice bath; triethylamine (3.0 equiv., 3.70 mmol) and acid chloride (1.85 mmol; 1.5 equiv.) were then added drop wise. After 30 min, the reactions were kept at room temperature for 5 h. The precipitate formed was filtered and purified on silica gel 60 column chromatography with a mixture of hexane and ethyl acetate as eluents [26].

2-Oxo-*2H*-chromen-4-yl-3-bromobenzoate (**4**): White solid. Yield: 27.1% (0.207 mmol; 71.4 mg). M.P.: 127–128.7 °C.; TLC (7:3 hexane/EtOAc); R*_f_* = 0.63. IR ʋmax (KBr, cm^−1^): 3086 (C-H sp^2^), 2926 (C-H sp^3^), 1752 (C=O); 1734 (C=O, ester); 1629 (C=C, alkene); 1611, 1571 (C=C aromatic); 1241, 1104 (C-O). ^1^H NMR (400 MHz, CDCl_3_) δ: 8.36 (m; 1H, H-2′); 8.17 (ddd; *J* = 7.8; 1.7; 1.1 Hz; 1H, H-6′); 7.86 (ddd; *J* = 8.0; 2.0; 1.1 Hz; 1H, H-4′); 7.67 (dd; *J* = 7.9; 1.5 Hz; 1H, H-5); 7.62 (ddd, *J* = 8.4; 7.3; 1.5, 1H, H-7); 7.50–7.45 (m; 1H, H-5′); 7.42 (dd; *J* = 8.4; 1.0 Hz; 1H, H-8); 7.33 (ddd; *J* = 7.9; 7.4; 1.1 Hz; 1H, H-6); 6.60 (s; 1H; H-3). ^13^C NMR (100 MHz, CDCl_3_) δ: 161.51 (C-2) *; 161.38 (C-7′) *; 158.71 (C-4); 153.85 (C-8a); 137.86 (C-4′); 133.48 (C-7); 133.14 (C-2′); 130.73 (C-5′); 129.94 (C-1′); 129.16 (C-6′); 124.64 (C-5); 123.25 (C-3′); 122.81 (C-6); 117.40 (C-8); 115.62 (C-4a); 106.07 (C-3). MALDI-TOF (*m/z*) analyze: C_16_H_9_BrO_4_ calculated value [M + Na]^+^ = 366.9581, Found = 366.9481 [M + Na]^+^; calculated value [M + H]^+^ = 344.9763, Found [M + H]^+^ = 344.9733. * Interchangeable.

#### 4.1.3. Methodology for Obtaining Esters **9**–**12** Derived from Coumarin-3-Carboxylic Acid

Coumarin-3-carboxylic acid (1.233 mmol), aromatic alcohol (1.0 equiv.) and 4-(dimethylamino)-pyridine (DMAP, 0.1 equiv.) were dissolved in 4.0 mL dichloromethane, the mixture was subjected to constant stirring at room temperature. Then, a solution of dicyclohexylcarbodiimide (DCC, 1.1 equiv.) in dichloromethane (3.0 mL) was added; the reactions were kept under constant stirring and at room temperature for 24 h. The DCU formed was filtered and the products purified in silica gel 60 column chromatography, using a mixture of hexane and ethyl acetate in different proportions as eluent [27].

Benzyl 2-oxo-2H-chromene-3-carboxylate (**9**): White solid. Yield: 14.2% (0.148 mmol; 41.9 mg). M.P.: 87–88 °C (lit. 80–81 [58]; TLC (7:3 hexane/EtOAc); R*_f_* = 0.47. IR ʋmax (KBr, cm^−1^): 3053 (C-H sp^2^); 1758 (C=O ester), 1698 (C=O, lactone); 1619, 1569, 1459 (C=C aromatic); 1215, 1156 (C-O). ^1^H NMR (400 MHz, CDCl_3_) δ 8.53 (s; 1H; H-4); 7.65 (ddd; *J* = 8.7; 7.3; 1.6 Hz; 1H; H-7); 7.59 (dd; *J* = 7.8; 1.5 Hz; 1H; H-5); 7.50–7.46 (m; 2H; H-2′; H-6′); 7.41–7.30 (m; 5H; H-6; H-8; H-3′; H-5′; H-4′); 5.39 (s; 2H; H-7′). ^13^C NMR (100 MHz, CDCl_3_) δ 162.91 (C-9, C=O); 156.71 (C-2); 155.32 (C-8a); 149.02 (C-7); 135.50 (C-1′); 134.59 (C-4); 129.68 (C-5); 128.77 (C-3′; C-5′); 128.55 (C-4′); 128.45 (C-2′; C-6′); 124.97 (C-6); 118.07 (C-4a); 117.93 (C-3); 116.92 (C-8); 67.58 (C-7′) [59].

4-Methoxybenzyl 2-oxo-*2H*-chromene-3-carboxylate (**10**): Yellow solid. Yield: 29.1% (95 mg; 0.306 mmol). M.P.: 113–114 °C.; TLC (7:3 hexane/EtOAc); R*_f_* = 0.34. IR ʋmax (KBr, cm^−1^): 3049 (C-H sp^2^); 2940 (C-H sp^3^); 1764 (C=O), 1719 (C=O); 1610, 1514, 1454 (C=C aromatic); 1244, 1130 (C-O). ^1^H NMR (500 MHz, CDCl_3_) δ 8.49 (s, 1H; H-4), 7.62 (ddd, *J* = 8.6; 7.4; 1.5 Hz, 1H; H-7), 7.57 (dd, *J* = 7.8, 1.4 Hz, 1H; H-5), 7.41 (d, *J* = 8.6 Hz; 2H; H-2′; H-6′), 7.34–7.30 (m; 2H; H-6; H-8), 6.90 (d, *J* = 8.7 Hz; 2H; H-3′; H-5′), 5.31 (s, 2H; C-7′), 3.80 (s; 3H; H-8′). ^13^C NMR (125 MHz; CDCl_3_) δ 162.92 (C-9, C=O); 159.92 (C-2); 156.70 (C-4′); 155.30 (C-8a); 148.80 (C-7); 134.49 (C-4); 130.40 (C-2′; C-6′); 129.62 (C-5); 127.63 (C-1′); 124.93 (C-6); 118.22 (C-4a); 117.95 (C-3); 116.89 (C-8); 114.15 (C-3′; C-5′); 67.45 (C-7′); 55.41 (C-8′). MALDI-TOF (*m/z*): C_18_H_14_O_5_ calculated value (M + Na)^+^ = 333.0738, Found = 333.0733.

3-Methoxybenzyl 2-oxo-*2H*-chromene-3-carboxylate (**11**): Yellow solid. Yield: 37.1% (121 mg, 0.3899 mmol). M.P.: 101–102 °C.; TLC (7:3 hexane/EtOAc); R*_f_* = 0.34. IR ʋmax (KBr, cm^−1^): 3051 (C-H sp^2^); 2935 (C-H sp^3^); 1758 (C=O), 1702 (C=O); 1618, 1567, 1519 (C=C aromatic); 1250, 1102 (C-O). ^1^H NMR (500 MHz, CDCl_3_) δ 8.50 (s, 1H; H-4); 7.61 (ddd; *J* = 8.6; 7.4; 1.6 Hz; 1H; H-7); 7.56 (dd; *J* = 7.8; 1.5 Hz; 1H; H-5); 7.33–7.19 (m; 3H; H-6; H-8; H-5′); 7.02–6.99 (m; 2H; H-6′; H-2′); 6.84 (dd; *J* = 8.4; 2.4 Hz; 1H; H-4′) 5.33 (s, 2H; H-7′); 3.79 (s, 3H; H-8′). ^13^C NMR (125 MHz; CDCl_3_) δ 162.91 (C=O); 159.93 (C-2); 156.69 (C-3′); 155.33 (C-8a); 149.02 (C-7); 137.02 (C-1′); 134.57 (C-4); 129.78 (C-5); 129.68 (C-5′); 124.96 (C-6); 120.41 (C-6′); 118.09 (C-4a); 117.93 (C-3); 116.89 (C-8); 114.19 (C-2′); 113.62 (C-4′); 67.38 (C-7′); 55.40 (C-8′). MALDI-TOF (*m/z*): C_18_H_14_O_5_ calculated value (M + Na)^+^ = 333.0738, found = 333.0744.

4-Methylbenzyl 2-oxo-*2H*-chromene-3-carboxylate (**12**): Crystalline white solid. Yield: 38.1% (118 mg, 0.401 mmol). M.P.: 103–104.2 °C.; TLC (7:3 hexane/EtOAc); R*_f_* = 0.5. IR ʋmax (KBr, cm^−1^): 3077 (C-H sp^2^); 2938 (C-H sp^3^); 1757 (C=O), 1708 (C=O); 1610, 1567, 1491 (C=C aromatic); 1247, 1135 (C-O). ^1^H NMR (400 MHz, CDCl_3_) δ 8.51 (s, 1H; H-4); 7.63 (ddd; *J* = 8.5; 7.3; 1.6 Hz; 1H; H-7); 7.58 (dd; *J* = 7.8; 1.6 Hz; 1H; H-5); 7.38–7.29 (m; 4H; H-6; H-8; H-3′; H-5′); 7.19 (d, *J* = 7.8 Hz; 2H; H-2′; H-6′); 5.34 (s, 2H; H-7′); 2.36 (s, 3H; H-8′). ^13^C NMR (100 MHz; CDCl_3_) δ 162.86 (C=O); 156.74 (C-2); 155.29 (C-8a); 148.92 (C-7); 138.47 (C-4′); 134.,55 (C-4); 132.47 (C-1′); 129.65 (C-5); 129.43 (C-3′; C-5′); 128.69 (C-2′; C-6′); 124.96 (C-6); 118.08 (C-4a); 117.92 (C-3); 116.90 (C-8); 67.57 (C-7′); 21.34 (C-8′). LC-MS/MS: C_18_H_14_O_4_ Calculated value (M + H^+^) = 295.0972. Found = 295.0976.

#### 4.1.4. Methodology for Obtaining Amides **13**–**19** Derived from Coumarin-3-Carboxylic Acid

To a solution of the organic acid (0.515 mmol) and the amine (1.0 equiv.) in dimethylformamide (2.0 mL) was added triethylamine (1.0 equiv.). The reaction medium was subjected to magnetic stirring and refrigeration (ice bath) for the addition of *py*BOP (benzotriazole-1-yloxytripyrrolidinophosphonium hexafluorophosphate) dissolved in 2 mL of dichloromethane. After 30 min, the reactions remained at room temperature and constant stirring for 24 h. At the end of the reaction process the products were extracted with dichloromethane (3 × 10 mL); and the organic phase was treated with a 1N HCl solution (1 × 10 mL); then dried with anhydrous sodium sulfate (Na_2_SO_4_) and concentrated in a rotary evaporator [28]. The amides were purified by precipitation in ethyl ether. Derivative **18** required further purification using silica gel 60 column chromatography with hexane and ethyl acetate (8:2, Hex:AcOEt) as eluent.

*N*-benzyl-2-oxo-*2H*-chromene-3-carboxamide (**13**): Crystalline solid. Yield: 73.4% (0.387 mmol; 108 mg). M.P.: 132.4–133.2 °C (lit. 139.4–140.2 °C, [29]); TLC (6:4 hexane/EtOAc); R*_f_* = 0.58. IR ʋmax (KBr, cm^−1^): 3333 (N-H); 3056 (C-H sp^2^); 2935 (C-H sp^3^); 1720 (C=O, C-2); 1703 (C=O, amide); 1655 (C=C, alkene); 1612, 1567, 1533 (C=C aromatic). ^1^H NMR (400 MHz, CDCl_3_) δ 9.18 (s, 1H, H de N-H); 8.95 (s, 1H, H-4); 7.69 (dd; *J* = 7.7; 1.5 Hz 1H; H-5); 7.67–7.64 (m; 1H, H-7); 7.41–7.37 (m; 2H; H-2′; H-6′); 7.38–7.32 (m; 4H; H-6; H-8; H-3′; H-5′); 7.30–7.26 (m; 1H; H-4′); 4.67 (d; *J* = 5.9 Hz; 2H; H-7′). ^13^C NMR (100 MHz; CDCl_3_) δ 161.62 (C=O); 161.49 (C-2); 154.51 (8a); 148.68 (C-7); 137.96 (C-1′); 134.19 (C-4); 129.91 (C-5); 128.79 (C-3′; C-5′); 127.78 (C-2′; C-6′); 127.55 (C-4′); 125.39 (C-6); 118.69 (C-4a); 118.45 (C-3); 116.71 (C-8); 43.92 (C-7′) [29].

*N*-(4-methoxybenzyl)-2-oxo-*2H*-chromene-3-carboxamide (**14**): White solid. Yield: 53.4% (87 mg, 0.2812 mmol). M.P.: 142.3–143.8 °C (lit: 145.1–145.7 °C, [29]); TLC (6:4 hexane/EtOAc); R*_f_* = 0.45. IR ʋmax (KBr, cm^−1^): 3326 (N-H); 3047 (C-H sp^2^); 2941 (C-H sp^3^); 1719 (C=O); 1703 (C=O amide); 1657 (C=C alkene); 1610, 1575, 1535 (C=C aromatic). ^1^H NMR (500 MHz, CDCl_3_) δ 9.09 (s, 1H; H-NH); 8.94 (s, 1H; H-4); 7.70 (dd; *J* = 7.8; 1.4 Hz; 1H; H-5); 7.66 (ddd; *J* = 8.8; 7.5; 1.5 Hz; 1H; H-7); 7.39 (m; 2H; H-6; H-8); 7.29 (d; *J* = 8.6 Hz; 2H; H-2′; H-6′); 6.88 (d; *J* = 8.7 Hz; 2H; H-3′; H-5′); 4.60 (d; *J* = 5.8 Hz; 2H; H-7′); 3.80 (s, 3H; OCH_3_). ^13^C NMR (125 MHz; CDCl_3_) δ 161.55 (C=O) *; 161.52 (C-2) *; 159.18 (C-4′); 154.61 (C-8a); 148.63 (C-7); 134.19 (C-4); 130.20 (C-1′); 129.95 (C-5); 129.26 (C-2′; C-6′) 125.43 (C-6); 118.82 (C-4a); 118.67 (C-3); 116.79 (C-8); 114.28 (C-3′; C-5′); 55.46 (C-OCH_3_); 43.53 (C-7′) [29]. * interchangeable.

*N*-(2,4-dimethoxybenzyl)-2-oxo-*2H*-chromene-3-carboxamide (**15**): White solid. Yield: 54.3% (194 mg, 0.5716 mmol). M.P.:144.4–145.8 °C (lit. 142.9–143.7 °C, [29]); TLC (6:4 hexane/EtOAc); R*_f_* = 0.39. IR ʋmax (KBr, cm^−1^): 3342 (N-H); 3052 (C-H sp^2^); 2943 (C-H sp^3^) 1719 (C=O); 1707 (C=O amide); 1661 (C=C alkene); 1612, 1567, 1528 (C=C aromatic). ^1^H NMR (500 MHz, CDCl_3_) δ 9.25 (s, 1H; H-NH); 8.89 (s, 1H; H-4); 7.66 (dd; *J* = 7.7; 1.6 Hz; 1H; H-5) 1H); 7.63 (ddd; *J* = 8.4; 7.4; 1.6 Hz; 1H; H-7) 1H); 7.37–7.34 (m, 2H; H-6; H-8); 7.24 (d; *J* = 8.2 Hz; 1H; H-6′); 6.47 (d; *J* = 2.4 Hz; 1H; H-3′); 6.43 (dd; *J* = 8.3; 2.4 Hz; 1H; H-5′); 4.58 (d; *J* = 5.9 Hz; 1H; H-7′); 3.88 (s, 3H; H-8′); 3.79 (s, 3H; H-9′). ^13^C NMR (125 MHz; CDCl_3_) δ 161.36 (C=O) *; 161.16 (C-2) *; 160.70 (C-2′); 158.85 (C-4′); 154.53 (C-8a); 148.22 (C-7); 133.93 (C-4); 130.39 (C-5); 129.83 (C-6′); 125.27 (C-6); 118.98 (C-1′); 118.82 (C-4a); 118.72 (C-3); 116.68 (C-8); 104.06 (C-5′); 98.77 (C-3′); 55.52 (OCH_3_); 55.51 (OCH_3_); 39.49 (C-7′) [29]. * Interchangeable.

*N*-(3,4-dimethoxybenzyl)-2-oxo-*2H*-chromene-3-carboxamide (**16**): White solid, Yield: 53.3% (95 mg. 0.2799 mmol). M.P.: 169.3–170.5 °C.; TLC (6:4 hexane/EtOAc); R*_f_* = 0.29. IR ʋmax (KBr. cm^−1^): 3334 (N-H); 3053 (C-H sp^2^); 2942 (C-H sp^3^) 1720 (C=O); 1706 (C=O amide); 1653 (C=C alkene); 1609, 1567, 1519 (C=C aromatic). ^1^H NMR (500 MHz, CDCl_3_) δ 9.11 (s. 1H; H-NH); 8.93 (s; 1H; H-4); 7.69 (dd; *J* = 7.7; 1.5 Hz; 1H; H-5); 7.65 (ddd; *J* = 8.4; 7.4; 1.5 Hz; 1H; H-7); 7.41–7.35 (m; 2H; H-6. H-8); 6.91 (dd; *J* = 8.0; 2.0 Hz; 1H; H-6′); 6.89 (d; *J* = 1.9 Hz; 1H; H- 2′); 6.83 (d; *J* = 8.0 Hz; 1H; H-5′); 4.59 (d; *J* = 5.8 Hz; 2H; H-7′); 3.87 (s; 3H; O-CH_3_); 3.85 (s; 3H; O-CH_3_). ^13^C NMR (125 MHz; CDCl_3_) δ 161.53 (C=O) *; 161.50 (C-2) *; 154.59 (C-8a); 149.31 (C-3′; C-4′); 148.62 (C-7); 134.19 (C-4); 130.65 (C-1′); 129.92 (C-5); 125.41 (C-6); 120.23 (C-6′); 118.76 (C-4a); 118.61 (C-3); 116.75 (C-8); 111.47 (C-5′); 111.31 (C-2′); 56.08 (OCH_3_); 56.03 (OCH_3_); 43.83 (C-7′). MALDI-TOF (*m/z*): C_19_H_17_NO_5._ Calculated value = 339.1017. Found = 362.1017 [M + Na]^+^. * Interchangeable.

2-Oxo-*N*-phenethyl-*2H*-chromene-3-carboxamide (**17**): Crystalline solid, Yield: 41.1% (64 mg. 0.2181 mmol). M.P.: 175.2–176.3 °C (lit. 178–180 °C [60]. TLC (6:4 hexane/EtOAc); R*_f_* = 0.61. IR ʋmax (KBr, cm^−1^): 3327 (N-H); 3055 (C-H sp^2^); 2943 (C-H sp^3^); 1720 (C=O); 1707 (C=O amide); 1657 (C=C alkene); 1610; 1569; 1525 (C=C aromatic). ^1^H NMR (500 MHz, CDCl_3_) δ 8.89 (s; 1H; H-4); 8.87 (s; 1H; H-NH); 7.67 (dd; *J* = 7.7; 1.4 Hz; 1H; H-5); 7.64 (ddd; *J* = 8.3; 7.4; 1.6 Hz; 1H; H-7); 7.39–7.29 (m; 4H; H-6; H-8; H-3′; H-5′); 7.25–7.20 (m; 3H; H-2′; H-6′; H-4′); 3.71 (td; *J* = 7.3; 5.9; 2H; H-8′); 2.93 (t; *J* = 7.3 Hz; 2H). ^13^C NMR (125 MHz; CDCl_3_) δ 161.56 (C=O) *; 161.47 (C-2) *; 154.53 (C-8a); 148.33 (C-7); 138.91 (C-1′); 134.09 (C-4); 129.90 (C-5); 128.89 (C-3′; C-5′); 128.74 (C-2′; C-6′); 126.66 (C-4′); 125.36 (C-6); 118.78 (C-4a); 118.62 (C-3); 116.72 (C-8); 41.47 (C-8′); 35.77 (C-7′) [29]. * interchangeable.

*N*-butyl-2-oxo-*2H*-chromene-3-carboxamide (**18**): Crystalline solid, Yield: 41.5% (107 mg. 0.4365 mmol). M.P.: 90–91 °C (Lit.: 74–76 °C [61]); TLC (6:4 hexane/EtOAc); R*_f_* = 0.63. IR ʋmax (KBr. cm^−1^): 3330 (N-H); 3055 (C-H sp^2^); 2950 (C-H sp^3^) 1719 (C=O C-2); 1705 (C=O amide); 1661 (C=O alkene); 1609; 1568; 1535 (C=C aromatic). ^1^H NMR (400 MHz, CDCl_3_) δ 8.90 (s; 1H; H-4); 8.81 (s; 1H; H-NH); 7.68 (dd; *J* = 7.7; 1.5 Hz; 1H; H-5); 7.65 (ddd; *J*= 8.4; 7.4; 1.6 Hz; 1H; H-7); 7.40–7.34 (m; 2H; H-6; H-8); 3.45 (td; *J* = 7.1; 5.8 Hz; 2H; H-1′); 1,61 (quint, *J* = 7,4 Hz; 2H; H-2′); 1,41 (sext; *J* = 7,4 Hz; 2H; H-3′); 0.95 (t; *J* = 7.3 Hz; 3H). ^13^C NMR (100 MHz; CDCl_3_) δ 161.61 (C=O) *; 161.54 (C-2) *; 154.49 (C-8a); 148.34 (C-7); 134.06 (C-4); 129.90 (C-5); 125.37 (C-6); 118.79 (C-4a); 118.65 (C-3); 116.71 (C-8); 39.75 (C-1′); 31.53 (C-2′); 20.30 (C-3′); 13.87 (C-4′) (SONAM et al., 2022) [61]. * interchangeable.

*N*-isobutyl-2-oxo-*2H*-chromene-3-carboxamide (**19**): Crystalline solid, Yield: 47.2 % (122 mg. 0.4974 mmol). M.P.: 117.1–118.1 °C.; TLC (6:4 hexane/EtOAc); R*_f_* = 0.63 (6:4. hexane/EtOAc). IR ʋmax (KBr, cm^−1^): 3346 (N-H); 3055 (C-H sp^2^); 2963 (C-H sp^3^); 1718 (C=O); 1706 (C=O amide); 1655 (C=C alkene); 1609; 1567; 1520 (C=C aromatic). ^1^H NMR (400 MHz, CDCl_3_) δ 8.90 (s; 1H; H-4); 8.87 (s; 1H; H-NH); 7.68 (dd; *J* = 7.7; 1.5 Hz; 1H; H-5); 7.64 (ddd; *J*= 8.5; 7.4; 1.6 Hz; 1H; H-7); 7.40–7.34 (m; 2H; H-8; H-6); 3.29 (dd; *J* = 6.8; 5.9 Hz; 2H; H-1′); 1.91 (sept; *J* = 6.7 Hz; 1H; H-2′); 0.98 (d; *J* = 6.7 Hz; 6H; H-3′; H-3′’). ^13^C NMR (100 MHz; CDCl_3_) δ 161.65 (C=O) *; 161.58 (C-2) *; 154.48 (C-8a); 148.35 (C-7); 134.05 (C-4); 129.88 (C-5); 125.36 (C-6); 118.78 (C-4a); 118.67 (C-3); 116.70 (C-8); 47.35 (C1′); 28.56 (C-2′); 20.33 (C-3′; C-3′’) [61]. * interchangeable.

#### 4.1.5. Methodology for Obtaining Chalcone **20**

The 2-hydroxyacetophenone (2.203 mmol) and the aldehyde (2.203 mmol, 1.0 equiv.) were dissolved in 2.5 mL of methanol. The mixture was cooled in an ice bath with magnetic stirring to add a chilled solution of NaOH (60%). The reaction was kept in the ice bath for 45 min and at room temperature for 48h. The mixture was poured into ice water and the pH adjusted to 2.0 with a 6N hydrochloric acid (HCl) solution. The yellow precipitate formed was filtered and recrystallized with methanol to obtain the chalcone **20** (136 mg; 22.8%) [30].

(*E*)-3-(2-hydroxy-3-methoxyphenyl)-1-(2-hydroxyphenyl)prop-2-en-1-one (**20**): Yellow solid, Yield: 22.8% (136 mg; 0.503 mmol). M.P.: 180–181.8 °C (lit. 130–131 [62]); TLC (7:3 hexane/EtOAc); R*_f_* = 0.47. IR ʋmax (KBr, cm^−1^): 3346 (O-H); 1631 (C=O); 1607 (C=C alkene); 1584, 1562, 1481 (C=C aromatic). ^1^H NMR (400 MHz. DMSO-d_6_) δ 12.47 (s; 1H; H-2′-OH); 8.09 (d; J = 15.6 Hz; 1H; H-β); 8.04 (dd; *J* = 8.3; 1.6 Hz; 1H; H-6′); 7.81 (d; *J* = 15.6 Hz; 1H; H-α); 7.44 (ddd; *J* = 8.3; 7.2; 1.6 Hz; 1H; H-4′); 7.39 (dd; *J* = 8.0; 1.1 Hz; 1H; H-5); 6.94 (dd; *J* = 8.0; 1.2 Hz; 1H; H-6); 6.90–6.86 (m; 2H; H-5′; H-3′); 6.74 (t; *J* = 8.0 Hz; 1H; H-2); 3.72 (s; 3H). ^13^C NMR (100 MHz, DMSO-d_6_) δ 193.89 (C=O); 161.88 (C-2′); 148.15 (C-3); 146.99 (C-2); 140.17 (C-β); 136.20 (C-4′); 130.63 (C-6′); 121.50 (C-1); 120.92 (C-1′); 120.68 (C-6); 120.05 (C-α); 119.28 (C-5′) *; 119.25 (C-3′) *; 117.82 (C-5); 114.09 (C-4); 56.07 (C-7) [30]. * interchangeable.

#### 4.1.6. Methodology for Obtaining Derivatives **21**–**24** from 4-Chromanone

4-Chromanone (0.684 mmol) and the aldehyde (1.48 equiv., 2.01 mmol) were dissolved in methanol (2 mL), and pyrrolidine (1.48 equiv., 2.01 mmol) was added to the solution. The mixture was stirred at room temperature for 24 h. The mixture was poured into chilled water, the precipitate filtered and washed with water, dissolved in dichloromethane, dried with anhydrous sodium sulfate (Na_2_SO_4)_ and then concentrated [17].

(*E*)-3-Benzylidenechroman-4-one (**21**): crystalline white solid, Yield: 72% (0.492 mmol). M.P. 80.8–81.6 °C (lit.: 111 °C [63]). TLC (9:1 hexane/EtOAc); R*_f_* = 0.62. IR ʋmax (KBr, cm^−1^): 2857 (C-H sp^3^); 1668 (C=O); 1610 (C=C). ^1^H NMR (CDCl_3_, 400MHz): δ 8.03 (dd; *J* = 7.9; 1.7 Hz; 1H; H-5); 7.88 (*s*; 1H; H-9); 7.51–7.40 (m; 4H; H-6′; H-2′; H-3′; H-5′); 7.31–7.30 (m; 2H; H-7; H-4′); 7.09–7.05 (m; 1H; H-6); 6.98–6.95 (m; 1H; H-8); 5.35 (d; *J* = 1.9 Hz; 2H; H-2). ^13^C NMR (100 MHz, CDCl_3_) δ 182.31 (C-4); 161.24 (C-8a); 137.56 (C-9); 135.96 (C-7); 134.50 (C-3); 131.03 (C-1′); 130.08 (C-3′, C-5′); 129.56 (C-4′); 128.83 (C-2′, C-6′); 128.05 (C-5); 122.13 (C-4a); 122.01 (C-6); 118.02 (C-8); 67.71 (C-2) [64].

(*E*)-3-(4-Methoxybenzylidene)chroman-4-one (**22**): Crystalline yellow solid, Yield: 76% (0.518 mmol). M.P. 133–134.1 °C (lit.: 118 °C [63]. TLC (9:1 hexane/EtOAc); R*_f_* = 0.44. IR ʋmax (KBr, cm^−1^): 3079 (C-H sp^2^), 2837 (C-H sp^3^); 1665 (C=O); 1606 (C=C); 1510; 1478 (C=C aromatic). ^1^H NMR (CDCl_3_, 400MHz): δ 8.01 (dd; *J* = 7.9; 1.7 Hz; 1H; H-5); 7.83 (s; 1H; H-9); 7.47 (ddd; *J* = 8.8; 7.3; 1.7 Hz; 1H; H-7); 7.28–7.26 (m; 2H; H-2′; H-6′); 7.08–7.03 (m; 1H; H-6); 6.98–6.94 (m; 3H; H-8; H-3′; H-5′); 5.37 (d; *J* = 1.9 Hz; 2H; H-2); 3.85 (s; 3H; OCH_3_). ^13^C NMR (100 MHz, CDCl_3_) δ 182.26 (C-4); 161.09 (C-8a); 160.85 (C-4′); 137.40 (C-9); 135.76 (C-7); 132.16 (C-2′, C-6′); 129.02 (C-3); 127.99 (C-5); 127.14 (C-1′); 122.24 (C-4a); 121.93 (C-6); 117.93 (C-8); 114.39 (C-3′, C-5′); 67.90 (C-2); 55.51 (OCH_3_) [64].

(*E*)-3-(3-methoxybenzylidene)chroman-4-one (**23**): Yellow amorphous solid, Yield: 15.5% (0.1049 mmol). M.P. 78.7–80.4 °C (lit.: 85–86 °C [65]. TLC (9:1 hexane/EtOAc); R*_f_* = 0.50. IR ʋmax (KBr, cm^−1^): 3071 (C-H, sp^2^), 2978 (C-H, sp^3^), 1669 (C=O); 1614 (C=C); 1600; 1583 (C=C aromatic). ^1^H NMR (CDCl_3_; 400MHz): δ 8.02 (dd; *J* = 7.9; 1.7 Hz; 1H; H-5); 7.84 (s; 1H; H-9); 7.49 (ddd; *J* = 8.8; 7.2; 1.8 Hz; 1H; H-7); 7.36 (m; 1H; H-5′); 7.09–7.05 (m; 1H; H-6); 6.98–6.94 (m; 2H; H-2′; H-6′); 6.89–6.87 (m; 1H; H-8); 6.84 (m; 1H; H-4′); 5.35 (d; *J* = 1.9 Hz; 2H; H-2); 3.84 (s; 3H; OCH_3_). ^13^C NMR (100 MHz; CDCl_3_) δ 182.33 (C-4); 161.32 (C-8a); 159.85 (C-3′); 137.50 (C-9); 136.01 (C-7); 135.83 (C-3); 131.30 (C-1′); 129.88 (C-5′); 128.09 (C-5); 122.41 (C-6); 122.16 (C-4a); 122.05 (C-6′); 118.06 (C-8); 115.57 (C-4′); 115.20 (C-2′); 67.79 (C-2); 55.49 (OCH_3_) [65].

(*E*)-3-(2-Methoxybenzylidene)chroman-4-one (**24**): Crystalline white solid, Yield: 63.3% (0.4277 mmol). M.P. 102.1–103.6 °C. TLC (9:1 hexane/EtOAc); R*_f_* = 0.46. IR ʋmax (KBr, cm^−1^): 3086 (C-H, sp^2^), 2981 (C-H, sp^3^), 1677 (C=O); 1611 (C=C); 1578; 1559 (C=C aromatic). ^1^H NMR (400 MHz; CDCl_3_) δ 8.06 (dd; *J* = 7.9; 1.8 Hz; 1H; H-5); 8.03 (s; 1H; H-9); 7.49 (ddd; J = 8.4; 7.2; 1.7 Hz; 1H; H-7); 7.41–7.36 (m; 1H; H-6′); 7.10–7.07 (m; 2H; H-6; H-8); 7.03–7.01 (m; 1H; H-4′); 6.97–6.95 (m; 2H; H-3′; H-5′); 5.24 (d; *J* = 1.8 Hz; 2H; H-2); 3.85 (s; 3H; OCH_3_). ^13^C NMR (100 MHz; CDCl_3_) δ 182.54 (C-4); 161.39 (C-8a); 158.30 (C-2′); 135.77 (C-9); 133.97 (C-7); 131.26 (C-4′); 130.93 (C-3); 130.53 (C-6′); 128.02 (C-5); 123.59 (C-4a); 122.29 (C-1′); 121.85 (C-6); 120.37 (C-5′); 117.95 (C-8); 111.03 (3′); 68.18 (C-2); 55.59 (OCH_3_).

### 4.2. Antifungal Activity

Reference strains of *Candida* spp. were obtained from the American Type Culture Collection (ATCC, Rockville, MD, USA): *Candida albicans* ATCC 90028, *Candida albicans* ATCC 60193, *Candida tropicalis* ATCC 13803, *Candida krusei* ATCC 6258, *Candida parapsilosis* ATCC 22019 and *Candida glabrata* ATCC 90030. Nystatin, ketoconazole, DMSO (Dimethyl Sulfoxide), Tween 80% and Ergosterol were obtained from Sigma-Aldrich^®^ Chemical Co. (St. Louis, MO, USA). Sorbitol (anhydrous D-sorbitol) was purchased from INLAB^®^ (São Paulo, Brazil).

#### 4.2.1. Determination of Minimum Inhibitory Concentration (MIC)

The MIC was determined using the microdilution technique described by the Clinical and Laboratory Standards Institute, 2008 [66]. The yeast suspension was prepared in (Roswell Park Memorial Institute Medium)-RPMI broth and adjusted to a turbidity equivalent of 2.5 × 10^3^ CFU/mL, 530 nm, absorbance between 0.08–0.116. Serial dilutions of the compounds placed in 96-well U-bottom microtiter plates containing RPMI, in concentrations ranging from 1000 to 7.81 μg/mL. Nystatin and ketoconazole were used as controls and were tested at concentrations ranging, respectively, from 48 to 0.75 μg/mL and 16 to 0.125 μg/mL. These plates were incubated for 24 h at 35 °C, and the results were read by visually observing cell aggregates at the bottom of the wells. Cell viability controls, sterility of the culture medium, and 5% DMSO solution, were used to prepare the compounds solutions and performed simultaneously with the assay. The MIC was defined as the lowest concentration capable of inhibiting visible growth. The bioactivity of the compounds was determined from the MIC values and classified according to the following categories: (a) very strong bioactivity (MIC < 3.515 µg/mL; (b) strong bioactivity (MIC between 3.515 and 25 µg/mL); (c) moderate bioactivity (MIC between 26–100 µg/mL); (d) weak bioactivity (MIC from 101 to 500 µg/mL); and (e) very weak bioactivity (MIC in the range of 501–2000 µg/mL) [31].

#### 4.2.2. Determination of Minimum Fungicide Concentration (MFC)

To determine the MFC, 10 μL aliquots from the wells corresponding to MIC, MICx2, and MICx4 were subcultured on Sabouraud Dextrose Agar (KASVI1, kasv Imp and Dist de Prod/laboratories LTDA, Curitiba, Brazil). The plates were incubated for 24 h at 35 °C, and reading was performed by visually observing the fungal growth in the solid medium. MFC was defined as the lowest concentration capable of inhibiting visible growth (colonies forming in solid culture medium). The MFC/MIC ratio was calculated to determine whether the substance presented fungistatic (MFC/MIC greater than or equal to 4) or fungicidal (MFC/MIC less than 4) activity [67].

#### 4.2.3. Verification of Mode of Activity on the Fungal Cell Wall and Membrane

##### Ergosterol Test

The MIC in the presence of ergosterol was defined as the lowest concentration of the substance capable of promoting the inhibition of visible microbial growth. The assay was also performed using the microdilution technique, however, in the presence of exogenous ergosterol (Sigma-Aldrich, São Paulo, Brazil) at a concentration of 400 µg/mL. The *C. albicans* strain ATCC 90028 was used, and the assay was conducted as described for MIC determination. Nystatin was used as a positive control [68].

##### Sorbitol Assay

The sorbitol assay was performed using the microdilution technique, aiming to compare MIC values against *C. albicans* ATCC 90028 in the absence and presence of 0.8 μM sorbitol. To conduct this experiment, the procedures described for determining the MIC were performed. After this step, the plates were incubated at 35 °C, and readings were taken 24 h after the incubation period. Caspofungin, at an initial concentration of 4 µg/mL, was used as a positive control. Sorbitol is an osmotic protector of the fungal cell wall and upon addition of this substance. Higher MIC values in the media indicate a possible mode of action on targets that involve cell wall functions [32,33].

#### 4.2.4. Evaluation of the Antimicrobial Activity of Compound **8** on the Reduction of Fungal Biofilm

Aliquots (1000 µL) of the *C. tropicalis* ATCC 13803 inoculum containing about 10^6^ CFU/mL were transferred to a 48-well microdilution plate. Molecule concentrations previously determined by the values of MIC (60 µg/mL–0.258 µmol/mL), MICx2 (30 µg/mL–0.129 µmol/mL), MICx4 (15 µg/mL–0.0645 µmol/mL) were added to the wells, followed by incubation for 48 h at an optimal growth temperature of 35 °C, allowing the yeast to adhere. Likewise, the concentrations of nystatin (positive control) were defined at the following concentrations: 4 µg/mL (0.0043 µmol/mL), 2 µg/mL (0.0021 µmol/mL) and 1 µg/mL (0.0011 µmol/mL) which correspond respectively to the values of MIC, MICx2 and MICx4. Then, the wells were washed with phosphate-buffered saline (PBS) to remove weakly bound cells and fresh medium was added. The plates were incubated for 48 h at 35 °C. For biofilm quantification, the wells were washed twice with PBS, air dried for 45 min and stained with 0.4% crystal violet solution. Absorbance values were read at 600 nm using a plate reader [69]. The untreated biofilm served as a growth control. The assays were carried out in quadruplicate and with sterility control without the addition of microorganisms. The strain was chosen after preliminary screening among the strains used in the experiment to define the Minimum Inhibitory Concentration [70].

### 4.3. Molecular Modeling Study

#### 4.3.1. Targets Selection

Potential targets for compound **8** in *C. albicans* were identified employing the previously reported homology-based target fishing protocol [71,72]. For this, the probable targets for compound **8** were first predicted with the Similarity Ensemble Approach (SEA) method [73]. Computational target fishing methods, such as SEA, use the ligand-target interactions available on databases that are biased mainly toward human, mammal and bacterial information for predicting ligand-protein associations. For this reason, the targets identified by the SEA web server were subject to a Blast [74] search against the *C. albicans* (tax id: 5476) proteins contained in the Reference proteins (refseq_protein) database. Proteins from the fungus identical in at least 35% to any SEA predicted targets and with their sequences covered in at least 70% by the Blast alignment were considered as potential targets of compound **8** in *C. albicans*.

#### 4.3.2. Molecular Docking

OpenEye’s Omega [75,76] was used to obtain one initial three-dimensional (3D) conformation of compound **8** and partial atomic charges of type am1bcc were added to it with MolCharge (QUACPAC) [77]. Among the predicted targets of the compound, only FBA1 had a 3D structure deposited in the Protein Data Bank (PDB) database. Large loops are missing in this structure (PDB code 6lnk) and these were added according to the AlphaFold [78] model of the protein available on the EMBL-EBI repository. The remaining *C. albicans* proteins selected for modeling had no structure deposited in the PDB database, thus homology models were generated for them with the SwissModel web server [79]. Several homology models were generated for each target sequence and among these, the one with the highest QMEAN score was selected for modeling studies. The Gold software [80] was selected for molecular docking calculations that proceeded following the consensus protocol described in our previous publications [81,82]. Briefly, hydrogen atoms were added to the receptors before molecular docking calculations. The ligand binding site on each receptor was defined from the compounds co-crystallized with the templates explored for homology models. Cofactors such as NAD and FAD were manually transferred to the target proteins in the cases when these are relevant for protein function and are not added to the homology models. For docking, the side chains of the residues pointing to the cavity were considered as flexible. The search efficiency parameter of Gold was set to 200% and primary scoring took place with the ChemPLP scoring function. For each target protein, 30 different docking solutions were produced, and these were rescored with the GoldScore, ChemScore and ASP scoring functions implemented in Gold. The rescored poses were next subject to a consensus ranking procedure consisting of the scaling of the four scoring functions to Z-scores. The final Z-score for each ligand pose was computed as the average of the four individual Z-scores. Any ligand poses with aggregated Z-score higher than 1 was selected for additional analyses. If no ligand pose meeting the former criterion was found, only the top scored conformer was further analyzed.

#### 4.3.3. Molecular Dynamics Simulations and Estimation of Free Energies of Binding

Amber 20 [83] was used for molecular dynamics (MD) simulations as described in our previous publication [84]. The same preparation, energy minimization, heating, equilibration, and production runs protocol were applied to all complexes. The ff19SB and gaff2 force fields were employed to parametrize proteins and compound **8**, respectively. Topologies and force field modifications for the ligand were generated with antechamber, while for cofactors these were obtained from the Amber parameter database maintained by the Bryce Group at The University of Manchester (http://amber.manchester.ac.uk/index.html, (accessed on 20 December 2021)). Systems were enclosed in truncated octahedron boxes and solvated with OPC water molecules. Excess charges on the solvated systems were neutralized by adding either Na^+^ or Cl^−^ counterions. The solvated and neutralized complexes were energy minimized in two stages, the first one of which included constraints for all atoms except the solvent, while during the second one all constraints were removed. The energy minimized systems were then gradually heated from 0 to 300 K for 20 ps before proceeding to the equilibration stage. Equilibration took place in the NTP ensemble maintaining the temperature at 300 K and pressure set to 1 bar. The equilibrated systems were used as input to five different production runs, each one lasting for 4 ns. The atomic velocities were randomly re-initialized before each production run to obtain a better description of the complexes’ conformational space. Free energies of binding were predicted with the MM-PBSA method as implemented in Amber. For this, 20 MD snapshots were evenly extracted from each of the five production runs, totaling 100 MD complex conformations for MM-PBSA calculations. In addition, snapshots for free energy of binding calculations were selected from the 1 ns–4 ns interval. The ionic strength was set to 150 mM and default implicit solvent parameters were used.

### 4.4. ADMET Predictions

The ADMET predictions for compound **8** and the control ketoconazole were performed with the SwissADME and pkCSM web servers following the procedure described. SwissADME was employed to retrieve the physicochemical parameters and lipophilicity properties. On the other hand, the pkCSM server was used to predict the pharmacokinetics properties and toxicity of the compounds [85,86].

## 5. Conclusions

From the series of molecules tested against species of *Candida,* compound **8** exhibited the best antifungal profile, with strong activity against the two tested strains: *C. albicans* ATCC 90028 and *C. tropicalis* ATCC 13803, and moderate activity against *C. krusei* ATCC 6258. Our findings suggest the importance of alkyl chain length to antifungal activity in *O*-alkylated derivatives at the coumarin C-7 position. Among homoisoflavonoids, the bioactivity of derivatives **23** and **24** stands out; derivative **23** presented slightly higher antifungal capacity, with moderate activity against three strains tested, which may be related to the *m*-OCH_3_ substituent of ring B. Compound **8** also showed the ability to reduce *C. tropicalis* ATCC 13803 biofilm from 73% to 68% at concentrations of 0.268 µmol/mL to 0.067 µmol/mL, respectively. The mode of action studies of compounds **8** and **21**, did not evidence direct interaction with plasma membrane ergosterol or the fungal cell wall. Molecular modeling of **8** suggested a mechanism of action involving interaction with several pharmacological targets (a multi-target antifungal mechanism of action), involving interference in the redox balance of the *C. albicans* cell and plasma membrane synthesis, such that membrane impairment does not occur through direct interaction with its components, but through interferences in ergosterol synthesis. The ADMET properties of compound **8** are similar to those represented by the antifungal drug ketoconazole. Therefore, in further studies the development of an antifungal drug candidate may have improved ADMET properties over compound **8**. The results of this study may contribute to the development of new antifungal agents.

## Figures and Tables

**Table 1:** Minimum Inhibitory Concentration (MIC) of compounds against *Candida* spp. MIC values are expressed in μg/mL and μmol/mL.

Compounds	*C. albicans* ATCC 90028		*C. albicans* ATCC 60193		*C. tropicalis* ATCC 13803		*C. krusei* ATCC 6258		*C. parapsilosis* ATCC 22019		*C. glabrata* ATCC 90030	
	MIC (µg/mL)	MIC (µmol/mL)	MIC (µg/mL)	MIC (µmol/mL)	MIC (µg/mL)	MIC (µmol/mL)	MIC (µg/mL)	MIC (µmol/mL)	MIC (µg/mL)	MIC (µmol/mL)	MIC (µg/mL)	MIC (µmol/mL)
**1**	1000	4.89	-	-	-	-	-	-	-	-	-	-
**2**	-	-	-	-	-	-	-	-	-	-	-	-
**3**	-	-	-	-	-	-	31.25	0.103	-	-	-	-
**4**	-	-	-	-	-	-	-	-	-	-	-	-
**5**	125	0.612	500	2.44	500	2.44	250	1.22	500	2.44	500	2.44
**6**	62.5	0.306	-	-	125	0.612	125	0.612	-	-	-	-
**7**	-	-	-	-	-	-	-	-	-	-	-	-
**8**	15.62	0.067	250	1.07	15.62	0.067	62.50	0.269	250	1.07	500	2.15
**9**	-	-	-	-	-	-	-	-	-	-	-	-
**10**	-	-	-	-	-	-	-	-	-	-	-	-
**11**	-	-	-	-	-	-	-	-	-	-	-	-
**12**	-	-	-	-	-	-	-	-	-	-	-	-
**13**	-	-	-	-	-	-	-	-	-	-	-	-
**14**	-	-	-	-	-	-	-	-	-	-	-	-
**15**	-	-	-	-	-	-	-	-	-	-	-	-
**16**	-	-	125	0.368	-	-	-	-	250	0.736	500	1.47
**17**	-	-	-	-	-	-	-	-	-	-	-	-
**18**	-	-	250	1.01	-	-	-	-	-	-	-	-
**19**	250	1.02	1000	4.07	125	1.02	-	-	-	-	-	-
**20**	250	0.925	-	-	62.5	0.231	500	1.84	-	-	-	-
**21**	62.5	0.264	-	-	250	1.06	62.5	0.264	-	-	-	-
**22**	1000	3.75	-	-	62.5	0.234	-	-	-	-	-	-
**23**	62.5	0.234	500	1.87	62.5	0.234	62.5	0.234	500	1.87	500	1.87
**24**	62.5	0.234	250	0.938	62.5	0.234	500	1.87	500	1.87	500	1.87
**25**	1000	6.17	-	-	250	1.54	-	-	-	-	-	-
**26**	500	3.08	-	-	125	0.770	-	-	-	-	-	-
**27**	500	2.63	-	-	1000	5.26	-	-	-	-	-	-
**28**	1000	6.75	-	-	1000	6.75	1000	6.75	-	-	-	-
**29**	1000	7.34	1000	7.34	-	-	-	-	1000	7.34	1000	7.34
Nystatin	1.5	0.0016	1.5	0.0016	1.5	0.0016	1.5	0.0016	1.5	0.0016	1.5	0.0016
Ketoconazole	0.5	0.00094	0.5	0.00094	4	0.0078	0.5	0.00094	0.5	0.0078	0.5	0.00094
DMSO	-	-	-	-	-	-	-	-	-	-	-	-

**Table 2:** Minimum Fungicidal Concentration (MFC) of compounds against *Candida* spp. MFC values are expressed in μmol/mL.

Compounds	*C. albicans* ATCC 90028		*C. albicans* ATCC 60193		*C. tropicalis* ATCC 13803		*C. krusei* ATCC 6258		*C. parapsilosis* ATCC 22019		*C. glabrata* ATCC 90030	
	MFC	MFC/MIC *	MFC	MFC/MIC *	MFC	MFC/MIC *	MFC	MFC/MIC *	MFC	MFC/MIC *	MFC	MFC/MIC *
**1**	>4.89	1	-	-	-	-	-	-	-	-	-	-
**2**	-	-	-	-	-	-	-	-	-	-	-	-
**3**	-	-	-	-	-	-	0.206	2	-	-	-	-
**4**	-	-	-	-	-	-	-	-	-	-	-	-
**5**	1.22	2	-	-	>4.89	-	4.89	4	-	-	-	-
**6**	0.306	1	-	-	1.22	2	0.612	1	-	-	-	-
**7**	-	-	-	-	-	-	-	-	-	-	-	-
**8**	0.134	2	-	-	0.067	1	0.269	1	-	-	-	-
**9**	-	-	-	-	-	-	-	-	-	-	-	-
**10**	-	-	-	-	-	-	-	-	-	-	-	-
**11**	-	-	-	-	-	-	-	-	-	-	-	-
**12**	-	-	-	-	-	-	-	-	-	-	-	-
**13**	-	-	-	-	-	-	-	-	-	-	-	-
**14**	-	-	-	-	-	-	-	-	-	-	-	-
**15**	-	-	-	-	-	-	-	-	-	-	-	-
**16**	-	-	1.47	4	-	-	-	-	1.47	2	1.47	2
**17**	-	-	-	-	-	-	-	-	-	-	-	-
**18**	-	-	-	-	-	-	-	-	-	-	-	-
**19**	1.02	1	4.07	1	2.04	2	-	-	-	-	-	-
**20**	3.69	4	-	-	0.231	1	1.84	1	-	-	-	-
**21**	0.264	1	-	-	1.06	1	0.264	1	-	-	-	-
**22**	3.75	1	-	-	0.469	2	-	-	-	-	-	-
**23**	0.234	1	3.75	2	0.234	1	0.234	1	3.75	2	3.75	2
**24**	0.234	1	3.75	4	0.469	2	3.75	2	3.75	2	3.75	2
**25**	6.17	1	-	-	1.54	1	-	-	-	-	-	-
**26**	6.17	2	-	-	0.770	1	-	-	-	-	-	-
**27**	2.63	1	-	-	5.26	1	-	-	-	-	-	-
**28**	6.75	1	-	-	6.75	1	6.75	1	-	-	-	-
**29**	7.34	1	-	-	-	-	-	-	-	-	-	-
Nystatin	0.0016	1	0.0016	1	0.0016	1	0.0016	1	0.0016	1	0.0016	1
Ketoconazole	0.00094	1	0.00094	1	0.0078	1	0.00094	1	0.00094	1	0.00094	1
DMSO	-	-	-	-	-	-	-	-	-	-	-	-

* MFC/MIC < 4, fungicide activity; MFC/MIC ≥ 4 fungistatic activity.

**Table 3:** The effect of exogenous ergosterol (1.008 mM) on the MIC of **21**, **8** and nistatin against *C. albicans* ATCC 90028. Values are expressed in μmol/mL.

*C. albicans* ATCC 90028								
**21**			**8**			**Nistatin**		
Concentration (μmol/mL)	Without ergosterol	With ergosterol	Concentration (μmol/mL)	Without ergosterol	With ergosterol	Concentration (μmol/mL)	Without ergosterol	With ergosterol
4.23	-	-	4.30	-	-	0.051	-	-
2.11	-	-	2.15	-	-	0.025	-	+
1.05	-	-	1.07	-	-	0.012	-	+
0.52	-	-	0.53	-	-	0.006	-	+
0.26	-	-	0.26	-	-	0.003	-	+
0.13	+	+	0.13	-	-	0.0016	-	+
0.065	+	+	0.067	-	-	0.0008	+	+
0.032	+	+	0.033	+	+	0.0004	+	+

Note: +, fungal growth; -, no fungal growth.

**Table 4:** MIC values of **21**, **8** and caspofungin in the absence and presence of sorbitol (0.8 M) against strains of *C. albicans* ATCC 90028. Values are expressed in μmol/mL.

*C. albicans* ATCC 90028								
**21**			**8**			**Caspofungin**		
Concentration (μmol/mL)	Without sorbitol	With sorbitol	Concentration (μmol/mL)	Without sorbitol	With sorbitol	Concentration (μmol/mL)	Without sorbitol	With sorbitol
4.23	-	-	4.30	-	-	0.0036	-	-
2.11	-	-	2.15	-	-	0.0018	-	-
1.05	-	-	1.07	-	-	0.00091	-	+
0.52	-	-	0.53	-	-	0.00045	-	+
0.26	-	-	0.26	-	-	0.00022	-	+
0.13	+	+	0.13	-	-	0.00011	+	+
0.065	+	+	0.067	-	-	0.000056	+	+
0.032	+	+	0.033	+	+	0.000028	+	+

Note: +, fungal growth; -, no fungal growth.

**Table 5:** Potential targets of compound **8** in *C. albicans*.

UniProt Accession	ID	Description
A0A1D8PNK3	GRE3	D-xylose reductase
Q5ADT3	ALD2	Aldo-keto reductase
Q5ADT4	GCY1	Glycerol 2-dehydrogenase
A0A1D8PI24	ARA1	D-arabinose 1-dehydrogenase
A0A1D8PGT5	ALD5	Aldehyde dehydrogenase, mitochondrial
A0A1D8PSW6	ALD1	Aldehyde dehydrogenase
Q59T88	UGA2	Succinate-semialdehyde dehydrogenase
Q9URB4	FBA1	Fructose-bisphosphate aldolase
Q92206	ERG1	Squalene monooxygenase
A0A1D8PNS6	BTS1	Farnesyltranstransferase
Q5A985	HST2	NAD-dependent protein deacetylase

**Table 6:** ADMET predictions for compound **8** and ketoconazole.

Parameters	Compound 8	Ketoconazole
**Physicochemical properties**		
Molecular weight (g/mol)	232.28	531.43
Rotatable bonds	5	8
H-bond acceptors	3	5
H-bond donors	0	0
Fraction Csp3	0.36	0.38
TPSA (A3)	39.44	0.38
**Lipophilicity (Log Po/w)**		
iLOGP	3.06	3.96
XLOGP3	3.92	4.34
MLOGP	2.45	2.47
Consensus	3.33	3.56
**Absorption**		
Water solubility (log(mol/L))	−3.476	−3.464
Gastrointestinal absorption (%)	96.494	94.465
Skin permeability (log(Kp))	−2.064	−2.736
**Distribution**		
Blood-brain barrier permeability (log(BB))	0.112	−1.5
CNS permeability (log(PS))	−2.092	−2.512
VDss (human, log(L/kg))	0.205	0.216
**Metabolism**		
CYP1A2 inhibitor	Yes	Yes
CYP2C9 inhibitor	No	Yes
CYP2C19 inhibitor	Yes	Yes
CYP3A4 inhibitor	No	Yes
CYP2D6 inhibitor	No	No
**Excretion**		
Total Clearance (log(mL/min/kg))	1.077	0.587
Renal OCT2 substrate	No	Yes
**Toxicity**		
AMES toxicity	No	No
Max. tolerated dose (human, log(mg/kg/day))	0.505	0.949
hERG I inhibitor	No	No
hERG II inhibitor	No	Yes
Oral Rat Acute Toxicity (LD50, mol/kg)	2.144	3.174
Oral Rat Chronic Toxicity (LOAEL, log(mg/kg_bw/day))	2.251	0.677
Hepatotoxicity	No	Yes
Skin Sensitization	No	No

## Data Availability

Data is contained within article and Supplementary Materials.

## Supplementary Materials

The following supporting information can be downloaded at https://www.mdpi.com/article/10.3390/ph15060712/s1: Table S1: Results of docking compound **8** to its potential targets. Table S2: Predicted free energies of binding of compound 8 to its potential targets and its components according to the MM-PBSA method. Figures S1–S25: The RMSD plots. Spectrums S1–S24: The spectroscopic data of the unpublished compounds.
