# Supplementary material for: Synthesis of Coumarin and Homoisoflavonoid Derivatives and Analogs: The Search for New Antifungal Agents
Source: Pharmaceuticals (Basel). 2022 Jun 3;15(6):712. doi: 10.3390/ph15060712 (PMC9227125; doi:10.3390/ph15060712)
Supplement: Supplementary file 1 [file pharmaceuticals-15-00712-s001.zip › pharmaceuticals-1677248-supplementary.pdf]

Supporting Information

Synthesis of coumarin and homoisoflavonoid derivatives and analogs: The search for new antifungal agents

Alana R. Ferreira [1], Danielle da N. Alves [2], Ricardo D. de Castro [2], Yunierkis P. Castillo [3] and Damião P. de Sousa [1,*]

[1] *Laboratory of Pharmaceutical Chemistry, Department of Pharmaceutical Sciences, Federal University of Paraiba, João Pessoa, Paraíba 58051-900, Brazil; alana.rf@ltf.ufpb.br (A.R.F); damião_desousa@yahoo.com.br (D.P.d.S)*

[2] *Laboratory of Experimental Pharmacology and Cell Culture of the Health Sciences Center, Department Clinical and Social Dentistry, Federal University of Paraiba, João Pessoa, Paraíba 58051-900, Brazil; dnobregaalves@msn.com (D.d.N.A.); rcastro@css.ufpb.br (R.D.d.C)*

[3] *School of Physical and Mathematical Sciences, University of the Americas, Quito 170125, Ecuador; yunierkis@gmail.com*

* Correspondence: damião_desousa@yahoo.com.br; Tel.: +5583-9-9964-6611

Table S1. Results of docking **compound 8** to its potential targets.

Target	Pose	PLP[(a)]	Z_PLP[(b)]	GS[(c)]	Z_GS[(d)]	CS[(e)]	Z_CS[(f)]	ASP[(g)]	Z_ASP[(h)]	Aggregated Score
GRE3	1	52.29	1.27	5.93	0.87	20.72	1.83	33.37	2.02	1.50
	2	52.96	1.41	-0.08	0.22	19.28	1.31	32.2	1.68	1.16
ALD2	1	50.41	2.2	12.79	0.73	14.3	-0.21	30.99	2.01	1.18
	2	49.17	1.92	7.58	0.41	17.28	0.92	28.7	1.25	1.12
	3	43.27	0.59	21.4	1.26	17.83	1.12	28.2	1.08	1.01
GCY1	1	46.31	0.33	19.95	2.18	17.83	0.67	36.03	2.43	1.4
	2	47.28	0.63	13.8	1.71	18.43	0.99	29.07	0.87	1.05
	3	46.81	0.48	-6.42	0.16	20.3	1.99	31.99	1.53	1.04
ARA1	1	42.24	2.4	-28.15	0.43	10.25	1.75	17.71	1.31	1.47
	2	37.82	1.34	1.24	1.45	8.02	1.14	18.84	1.56	1.37
	3	34.84	0.63	-0.06	1.4	6.87	0.82	18.37	1.46	1.08
ALD5	1	67.85	1.33	26.67	0.67	26.92	0.98	39.54	1.14	1.03
ALD1	1	74.91	3.73	-3.66	-1.31	27.29	2.41	35.39	2.12	1.74
	2	61.93	0.87	11.17	0.03	27.65	2.57	34.33	1.73	1.3
UGA2	1	46.41	0.96	10.6	0.66	16.17	1.26	24.04	1.16	1.01
FBA1	1	53.41	2.07	26.35	1.19	13.25	2.07	15.34	0.74	1.52
	2	51.15	1.48	22.29	0.78	10.33	0.53	19.06	2.16	1.24
	3	53.06	1.98	23.89	0.94	11.92	1.37	12.76	-0.25	1.01
ERG1	1	50.34	1.19	7.73	0.87	25.34	1.54	27.51	1.13	1.18
	2	50.27	1.18	4.65	0.79	23.44	1.12	27.02	1.05	1.04
BTS1	1	59.4	1.43	33.17	1.13	25.32	1.77	32.55	0.32	1.16
	2	59.38	1.42	30.27	0.94	24.93	1.47	32.74	0.42	1.06
	3	61.3	2.32	9.73	-0.39	24.83	1.4	33.42	0.75	1.02
HST2	1	68.52	2.48	35.96	1.74	26.69	1.16	34.39	1.65	1.76
	2	62.57	1.05	21.8	0.37	27.67	1.65	32.45	1.09	1.04

[(a)] PLP score, [(b)] Scaled PLP score, [(c)] GoldScore score, [(d)] Scaled GoldScore score, [(e)] ChemScore score, [(f)] Scaled ChemScore score, [(g)] ASP score, [(h)] Scaled ASP score

Table S2. Predicted free energies of binding of **compound 8** to its potential targets and its components according to the MM-PBSA method. Energy values are expressed as kcal/mol.

Target	Conformer	MM-PBSA Component							ΔG TOTAL
		VD WAALS	EEL	EPB	ENPOLAR	EDISPER	ΔG gas	ΔG solv.	
GRE3	1	-22.02	-3.17	16.40	-16.95	29.58	-25.19	29.03	3.85
	2	-26.41	-3.80	15.66	-19.79	33.34	-30.21	29.21	-1.00
ALD2	1	-15.18	-4.22	11.02	-12.63	21.21	-19.40	19.60	0.20
	2	-15.94	-3.88	11.39	-12.62	21.40	-19.83	20.16	0.34
	3	-21.57	-9.05	15.62	-17.15	26.70	-30.62	25.18	-5.44
GCY1	1	-30.87	-18.53	31.05	-23.66	38.31	-49.41	45.71	-3.70
	2	-32.59	-6.64	23.39	-24.45	38.54	-39.23	37.48	-1.75
	3	-32.27	-7.35	23.35	-24.47	38.69	-39.62	37.56	-2.05
ARA1	1	-24.81	-4.99	16.09	-19.14	31.52	-29.81	28.47	-1.34
	2	-23.87	-1.66	13.69	-18.54	30.68	-25.53	25.82	0.29
	3	-28.84	-15.57	24.06	-22.82	36.08	-44.41	37.31	-7.10
ALD5	1	-35.50	-16.13	30.33	-24.99	41.68	-51.63	47.02	-4.61
ALD1	1	-37.92	-20.77	40.69	-27.05	44.92	-58.69	58.56	-0.13
	2	-34.99	-26.01	43.57	-25.17	42.73	-61.00	61.13	0.13
UGA2	1	-36.62	-8.22	30.32	-26.40	44.87	-44.83	48.79	3.96
FBA1	1	-34.46	-20.66	48.42	-22.40	39.38	-55.12	65.40	10.28
	2	-38.12	-12.21	41.92	-24.85	42.79	-50.33	59.86	9.54
	3	-26.55	-24.91	73.57	-18.72	32.73	-51.45	87.58	36.13
ERG1	1	-36.00	-2.55	18.50	-27.34	46.52	-38.54	37.68	-0.86
	2	-36.43	-20.97	32.57	-27.45	46.13	-57.39	51.25	-6.14
BTS1	1	-39.01	-8.61	26.66	-27.01	43.53	-47.62	43.18	-4.44
	2	-37.78	-3.52	32.61	-27.21	45.86	-41.30	51.25	9.95
	3	-37.94	-6.22	33.97	-26.96	45.55	-44.17	52.56	8.39
HST2	1	-37.90	-14.08	33.59	-26.77	44.70	-51.98	51.52	-0.46
	2	-33.08	-2.09	19.50	-24.42	40.12	-35.17	35.21	0.03

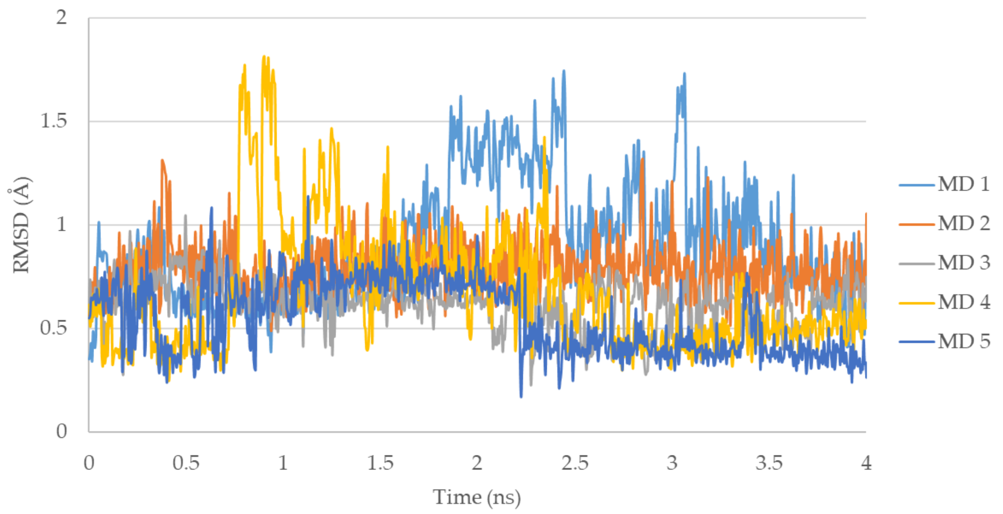

Figure S1. Ligand RMSD vs. time for the ALD1-compound **8** complex (docking pose 1).

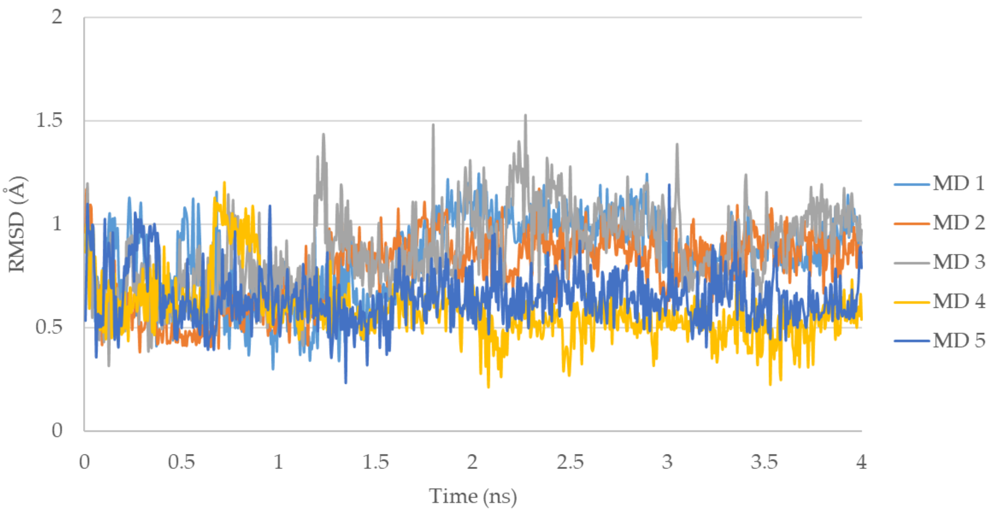

Figure S2. Ligand RMSD vs. time for the ALD1-compound **8** complex (docking pose 2).

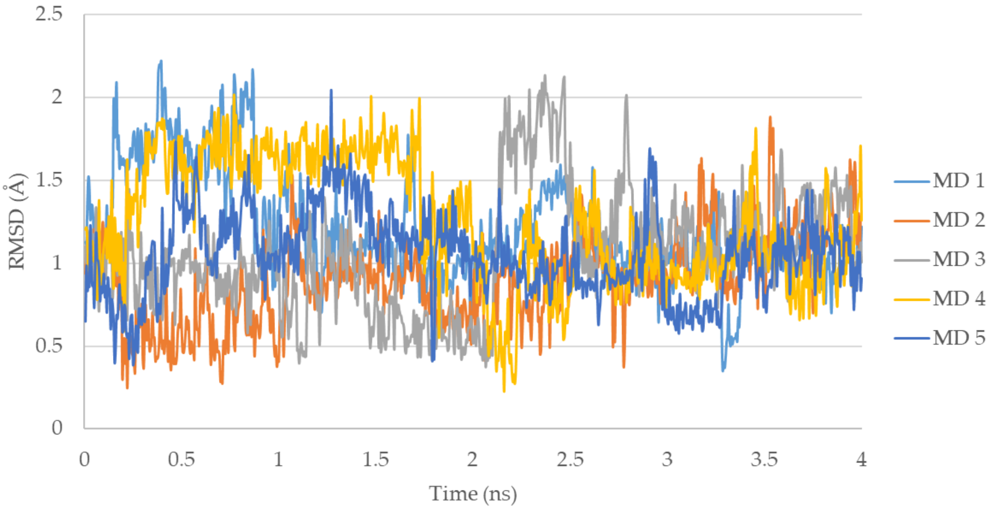

Figure S3. Ligand RMSD vs. time for the ALD2-compound **8** complex (docking pose 1).

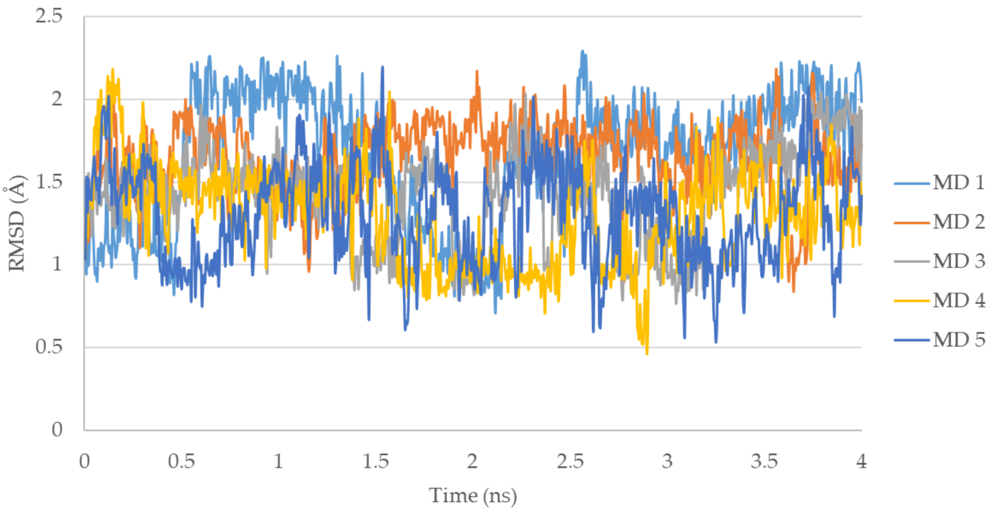

Figure S4. Ligand RMSD vs. time for the ALD2-compound **8** complex (docking pose 2).

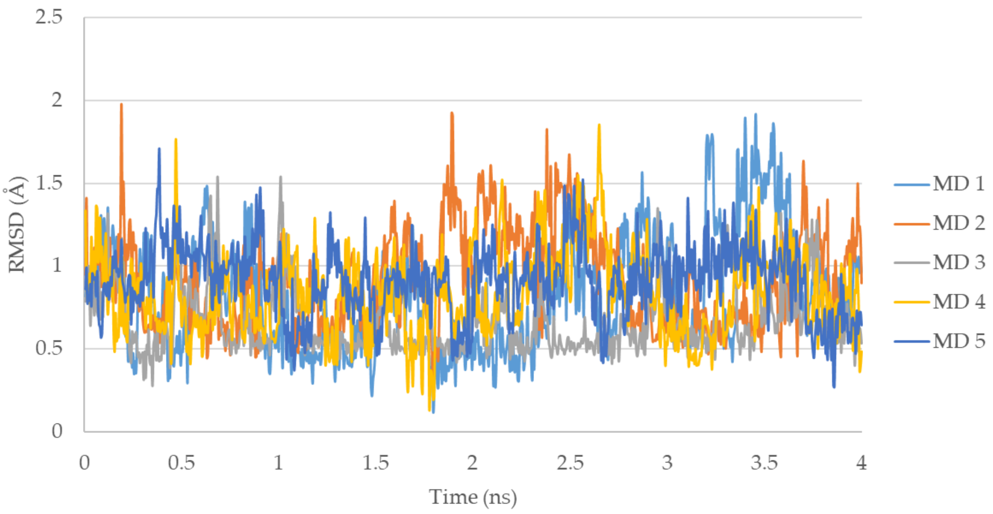

Figure S5. Ligand RMSD vs. time for the ALD2-compound **8** complex (docking pose 3).

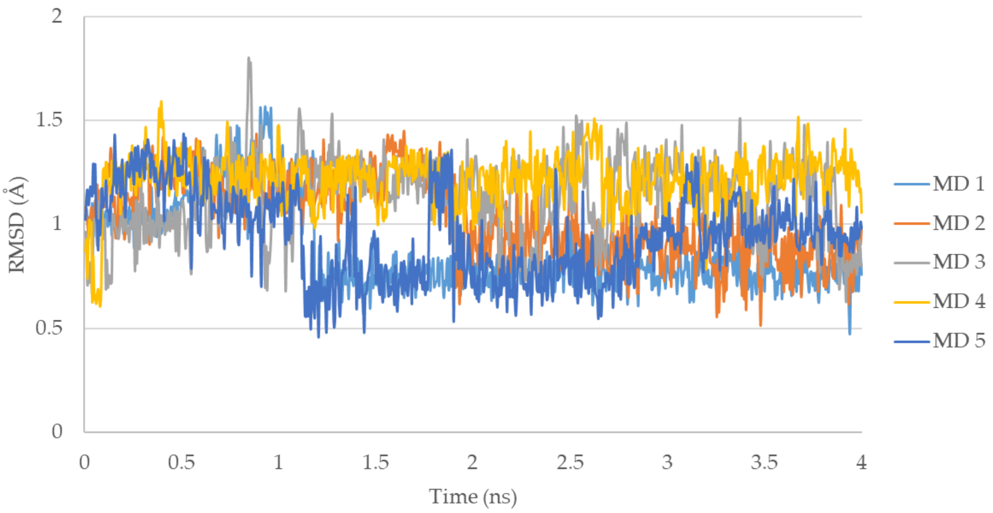

Figure S6. Ligand RMSD vs. time for the ALD5-compound **8** complex (docking pose 1).

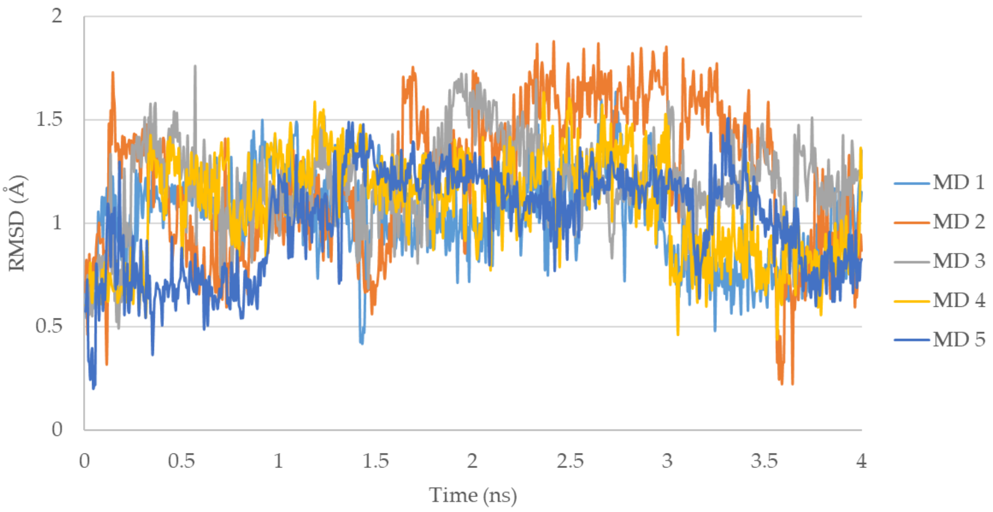

Figure S7. Ligand RMSD vs. time for the ARA1-compound **8** complex (docking pose 1).

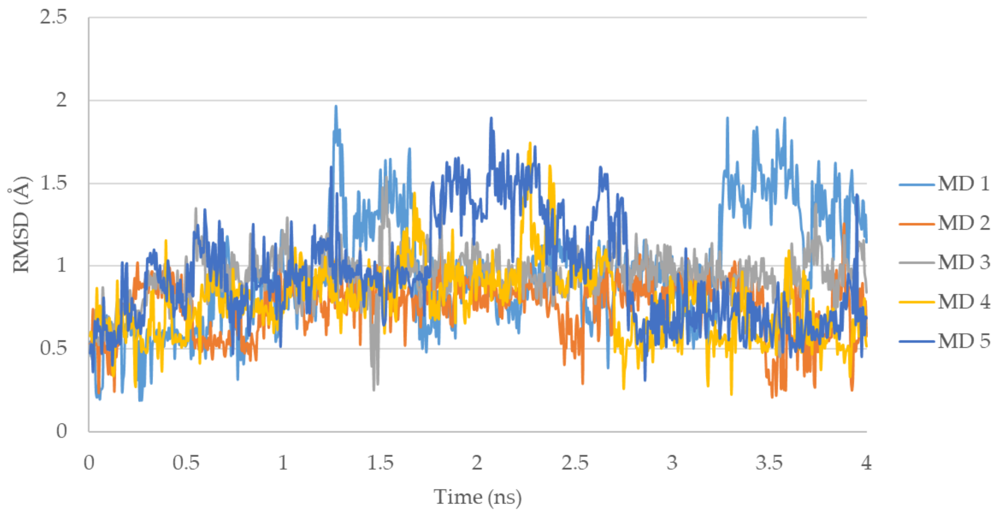

Figure S8. Ligand RMSD vs. time for the ARA1-compound **8** complex (docking pose 2).

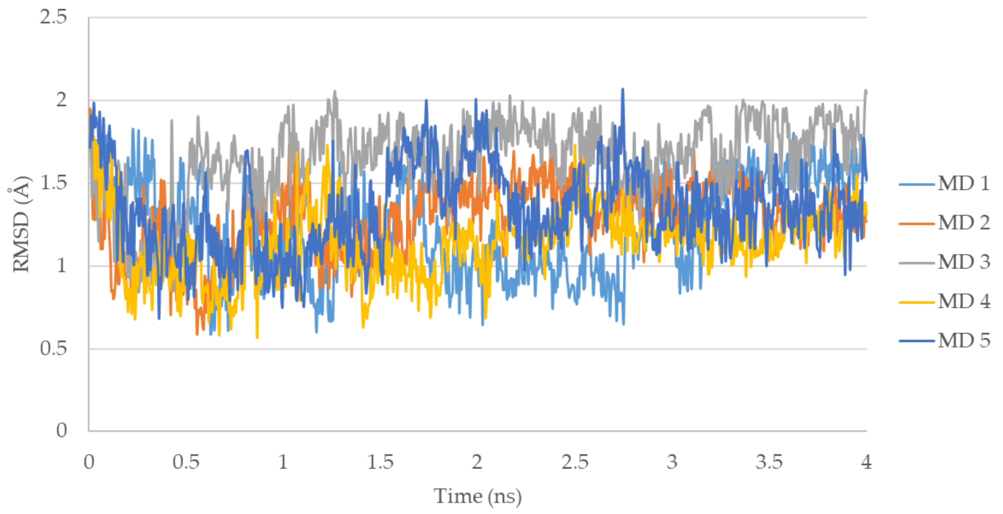

Figure S9. Ligand RMSD vs. time for the ARA1-compound **8** complex (docking pose 3).

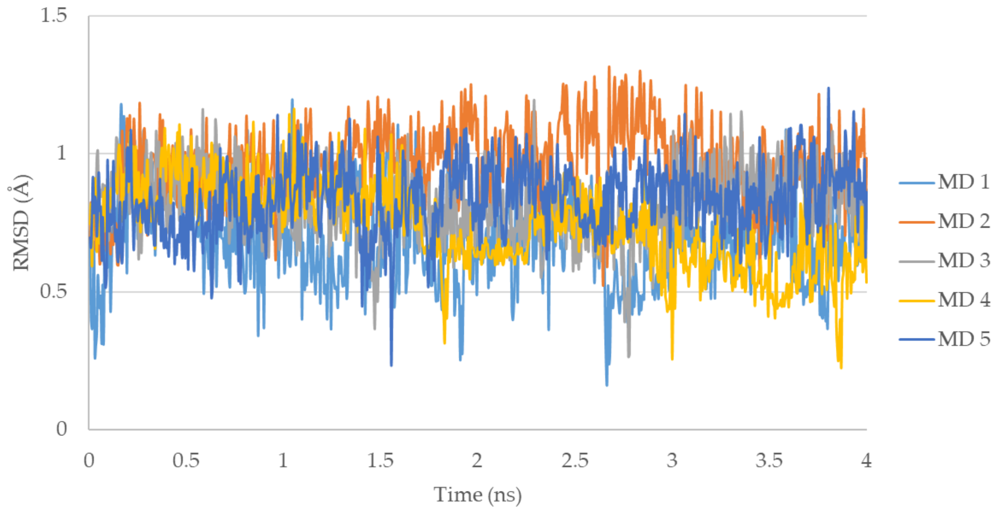

Figure S10. Ligand RMSD vs. time for the BTS1-compound **8** complex (docking pose 1).

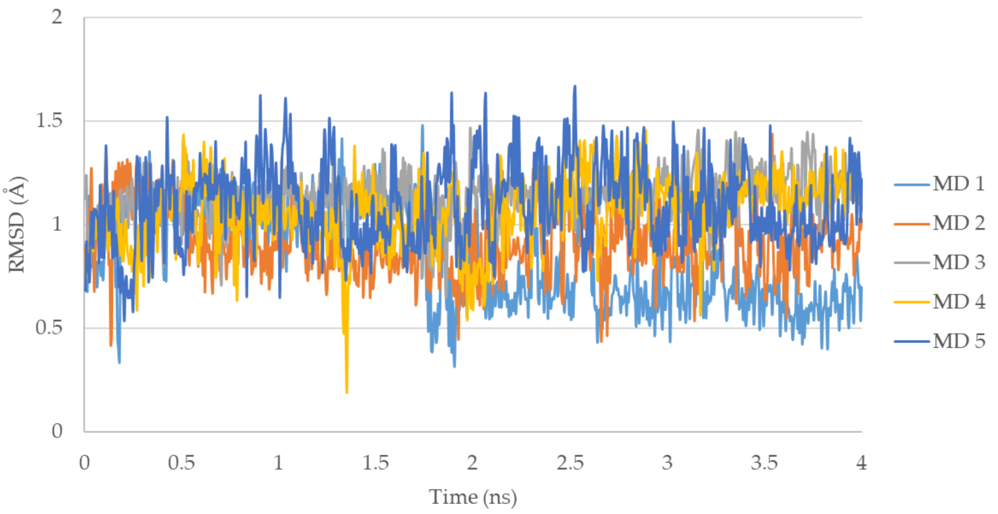

Figure S11. Ligand RMSD vs. time for the BTS1-compound **8** complex (docking pose 2).

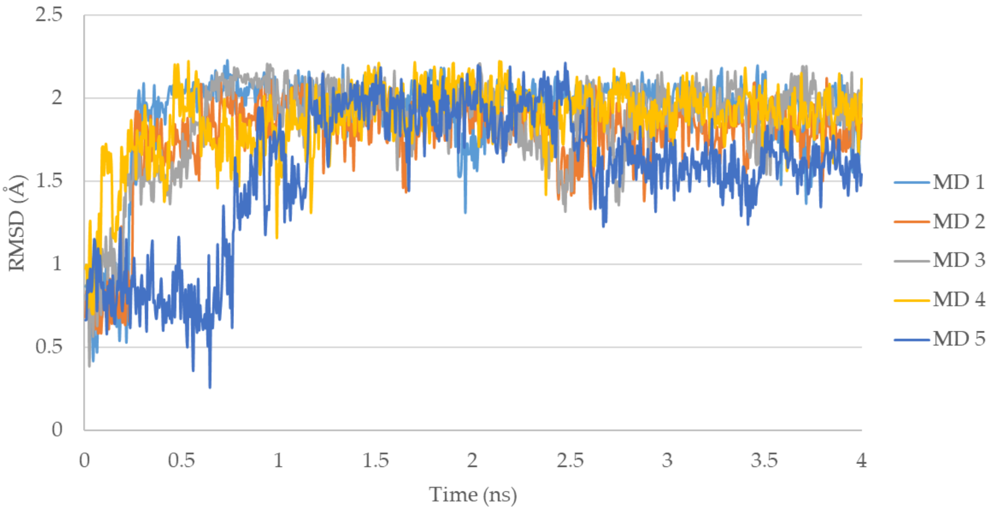

Figure S12. Ligand RMSD vs. time for the BTS1-compound **8** complex (docking pose 3).

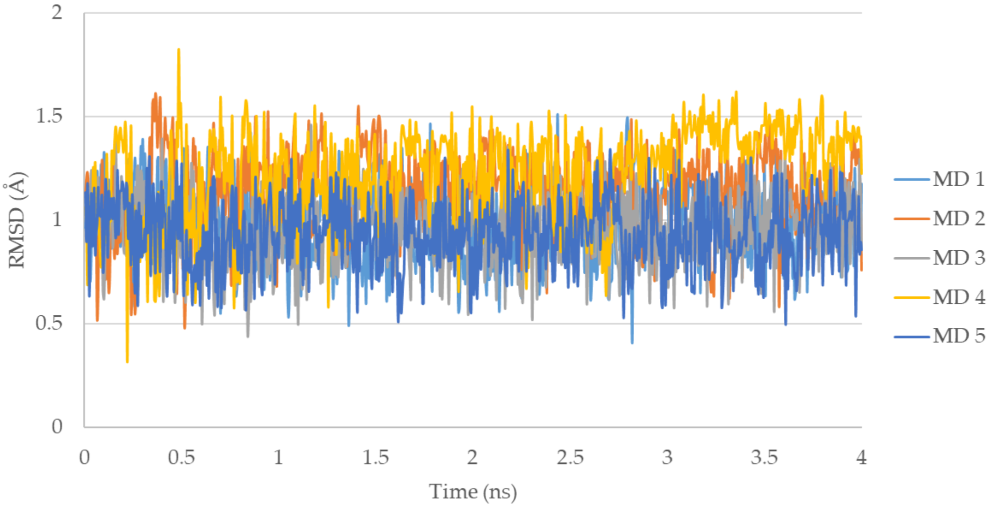

Figure S13. Ligand RMSD vs. time for the ERG1-compound **8** complex (docking pose 1).

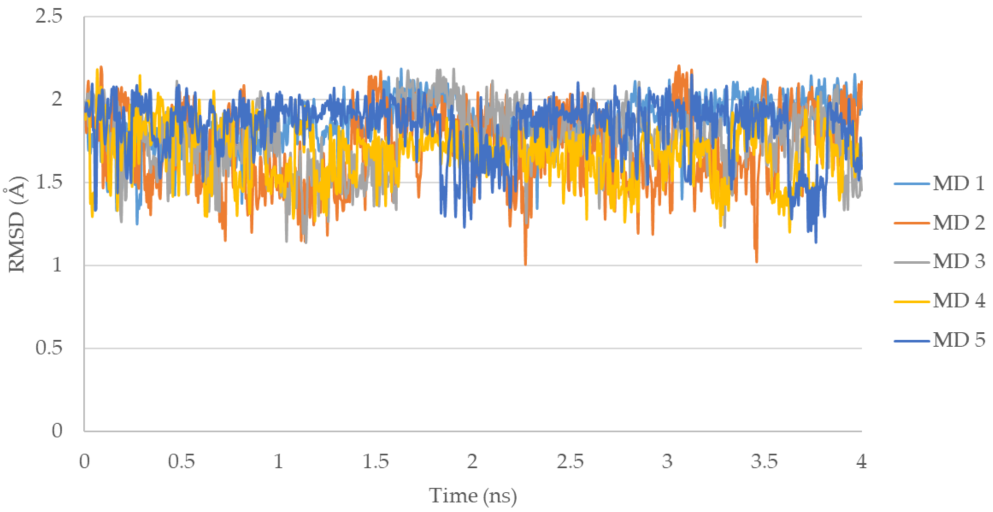

Figure S14. Ligand RMSD vs. time for the ERG1-compound **8** complex (docking pose 2).

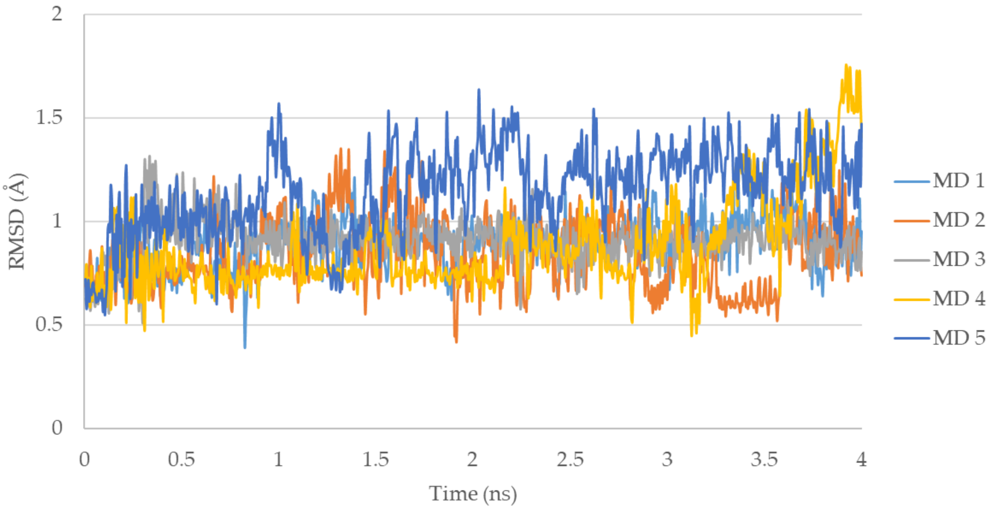

Figure S15. Ligand RMSD vs. time for the FBA1-compound **8** complex (docking pose 1).

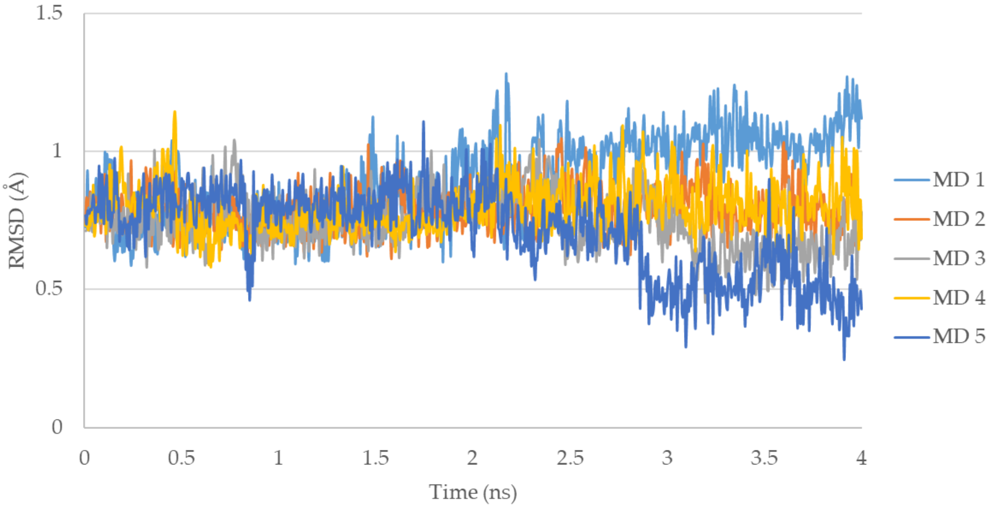

Figure S16. Ligand RMSD vs. time for the FBA1-compound **8** complex (docking pose 2).

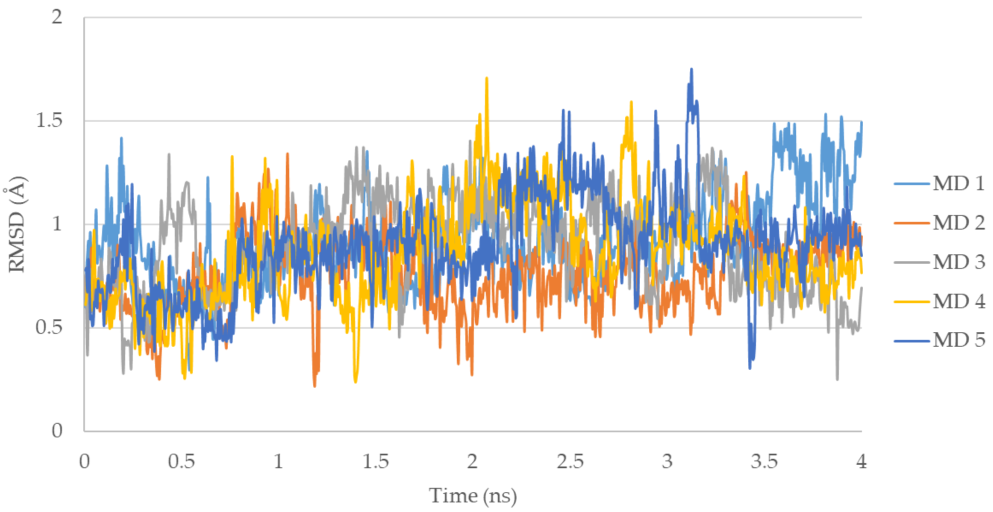

Figure S17. Ligand RMSD vs. time for the FBA1-compound **8** complex (docking pose 3).

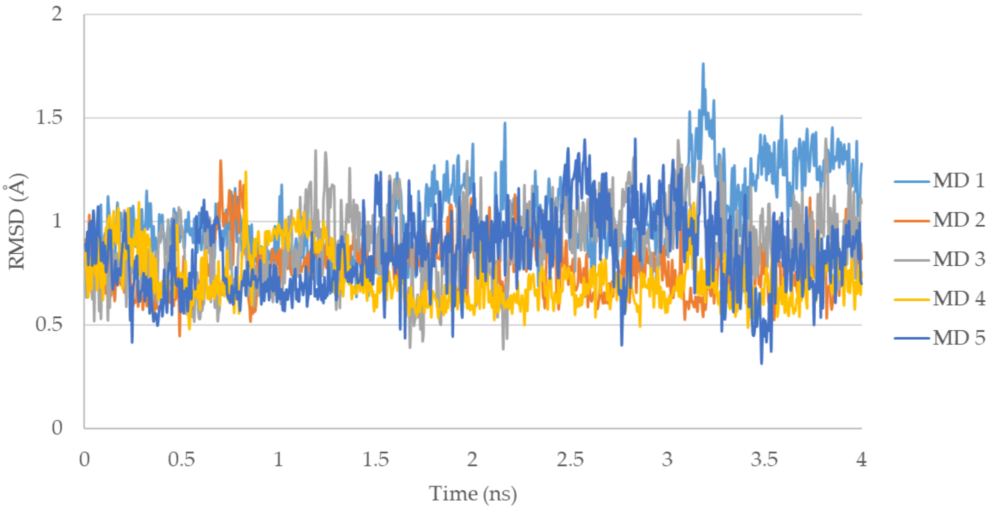

Figure S18. Ligand RMSD vs. time for the GCY1-compound **8** complex (docking pose 1).

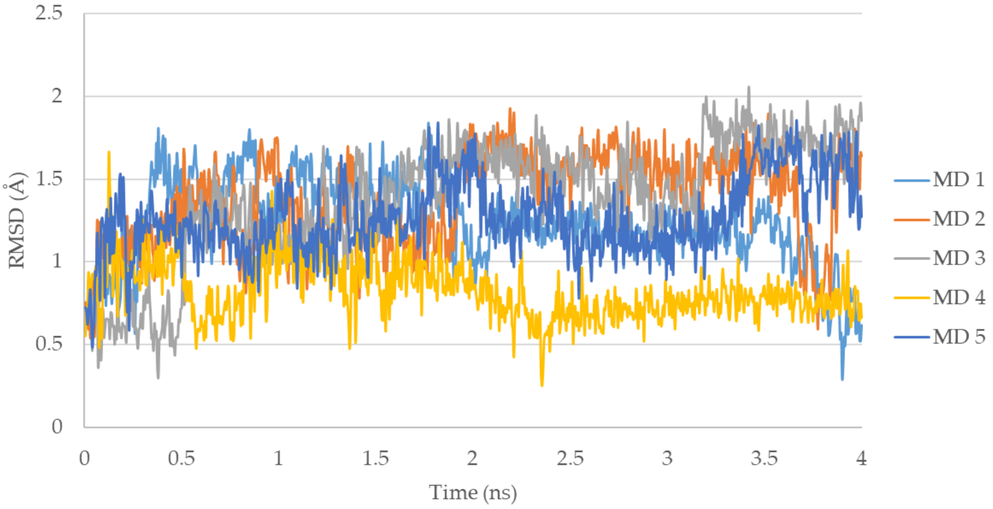

Figure S19. Ligand RMSD vs. time for the GCY1-compound **8** complex (docking pose 2).

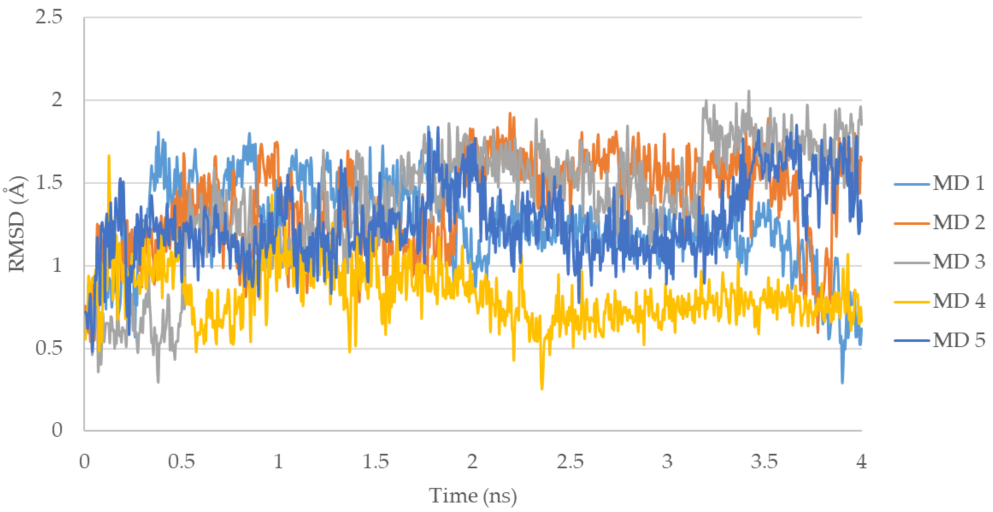

Figure S20. Ligand RMSD vs. time for the GCY1-compound **8** complex (docking pose 3).

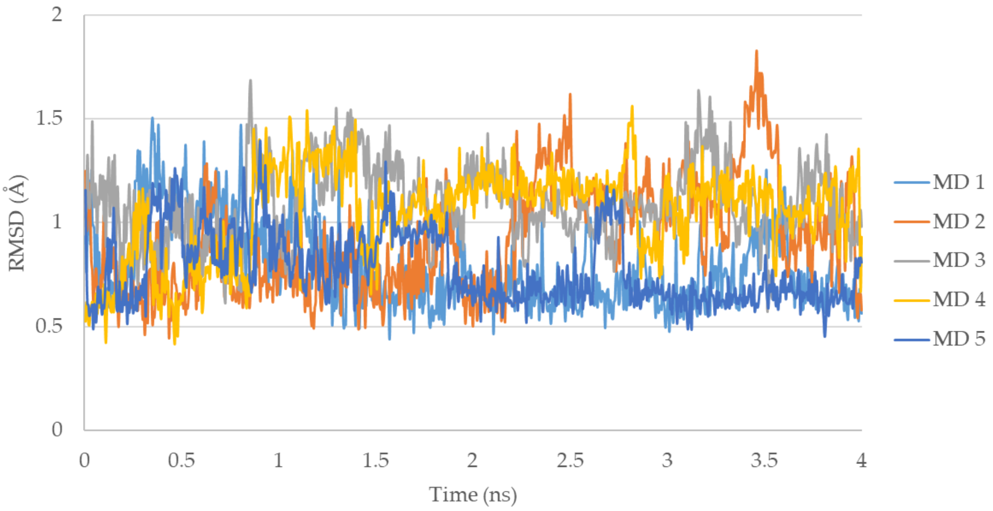

Figure S21. Ligand RMSD vs. time for the GRE3-compound **8** complex (docking pose 1).

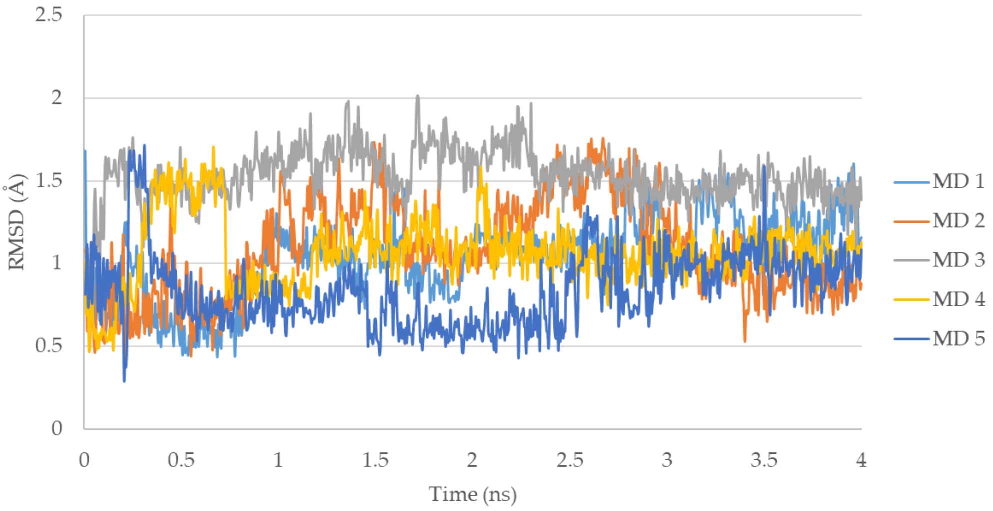

Figure S22. Ligand RMSD vs. time for the GRE3-compound **8** complex (docking pose 2).

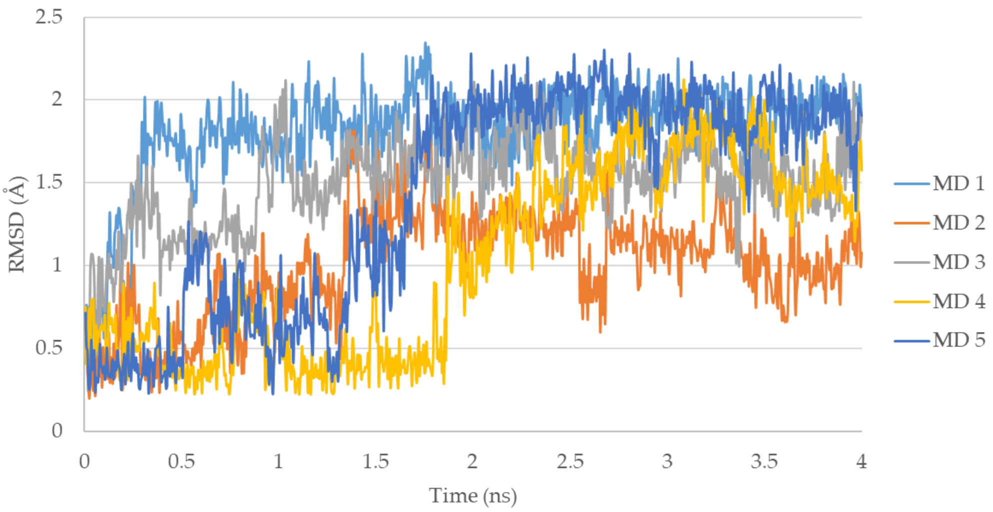

Figure S23. Ligand RMSD vs. time for the HTS2-compound **8** complex (docking pose 1).

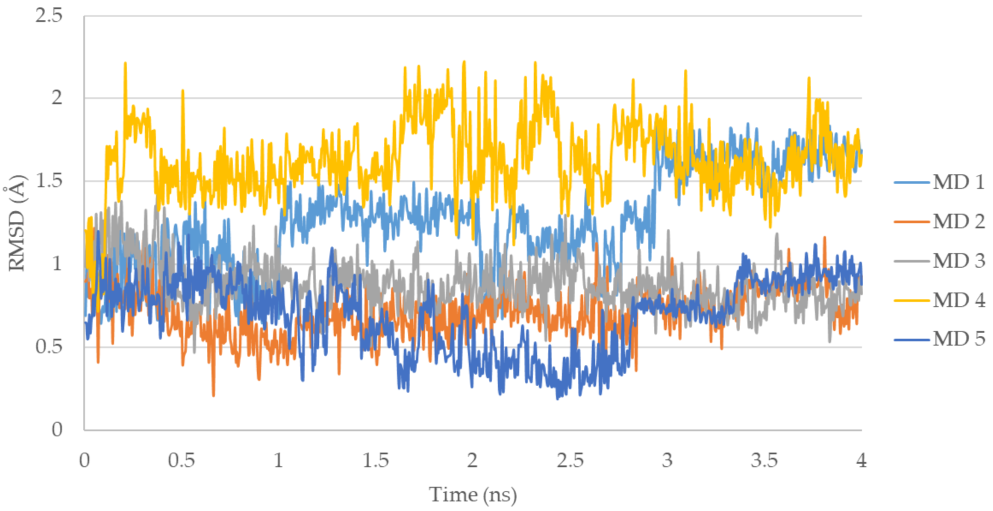

Figure S24. Ligand RMSD vs. time for the HTS2-compound **8** complex (docking pose 2).

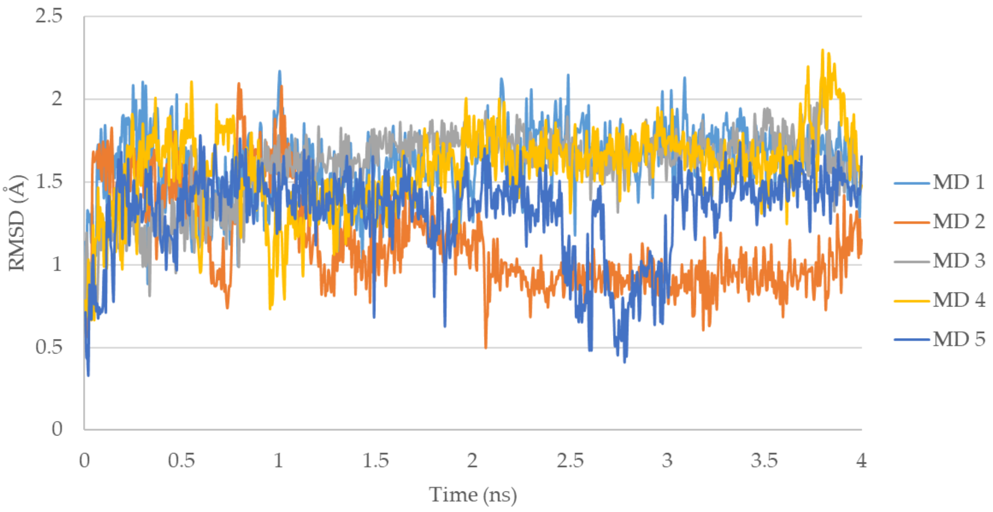

Figure S25. Ligand RMSD vs. time for the UGA2-compound **8** complex (docking pose 1).

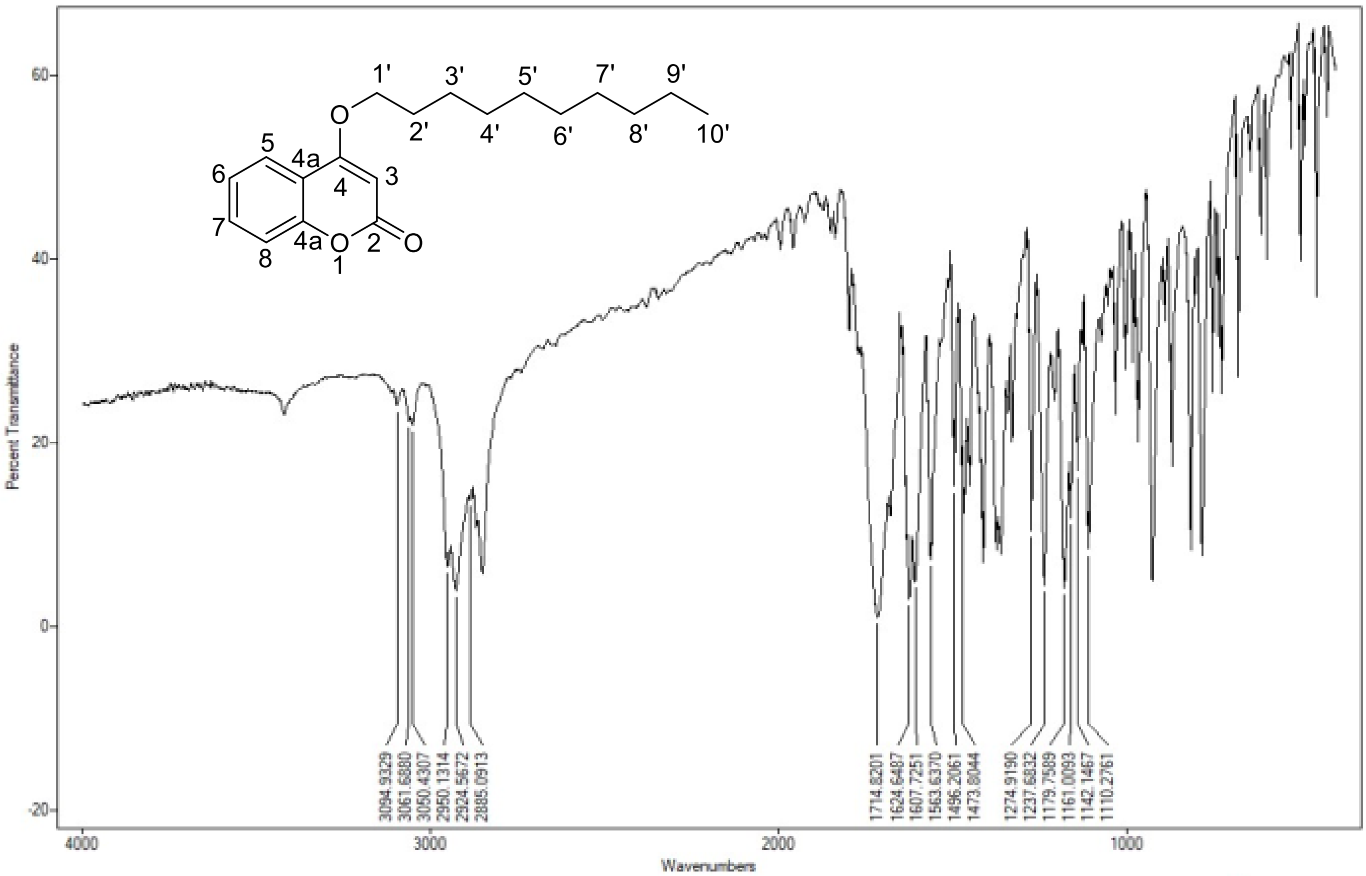

Spectum S1. Infrared sprectrum υmax (KBr, cm-1) of compound **3.**

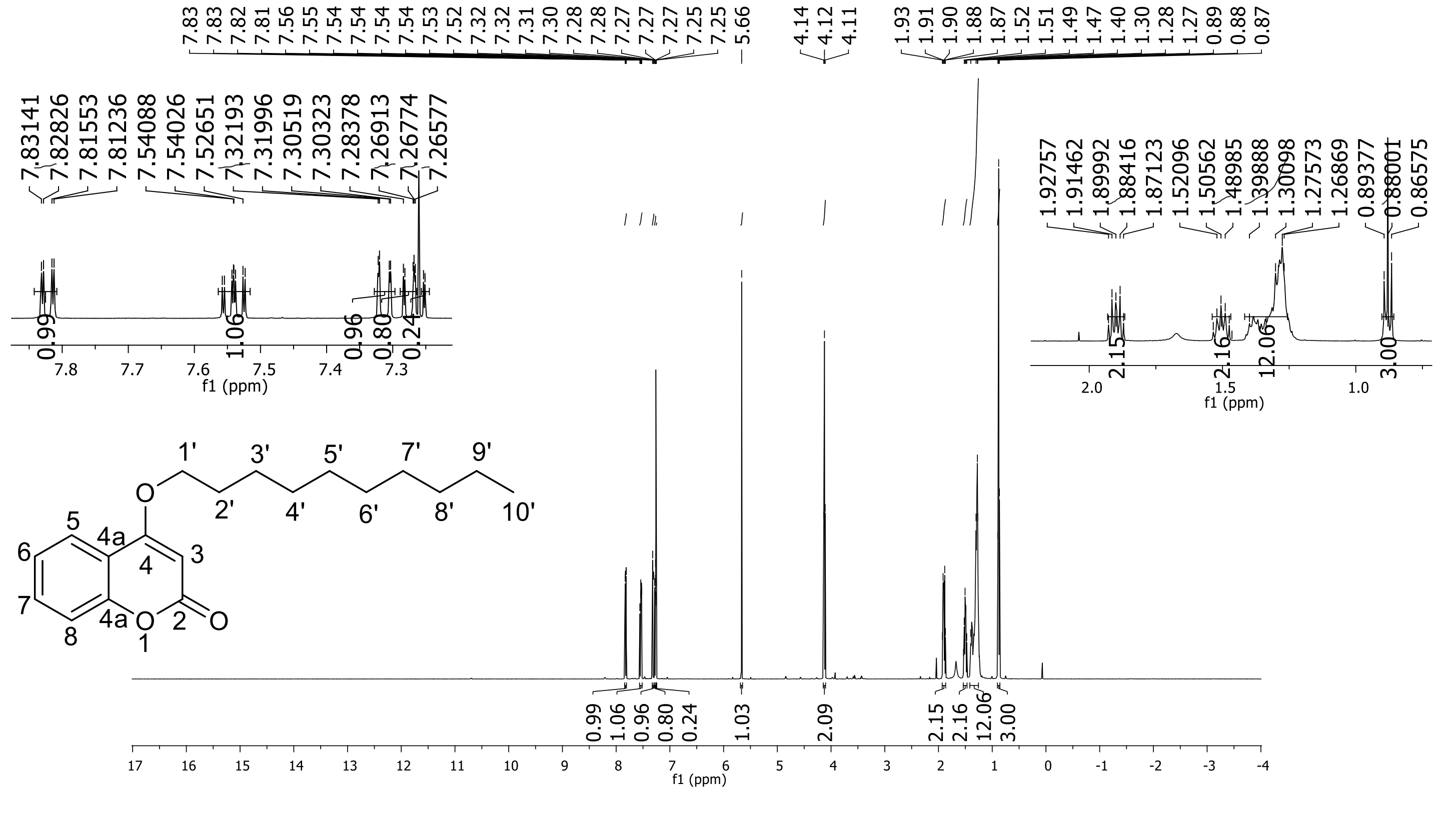

Spectrum S2. 1H NMR spectrum (500 MHz, $CDCl_3$) of compound **3.**

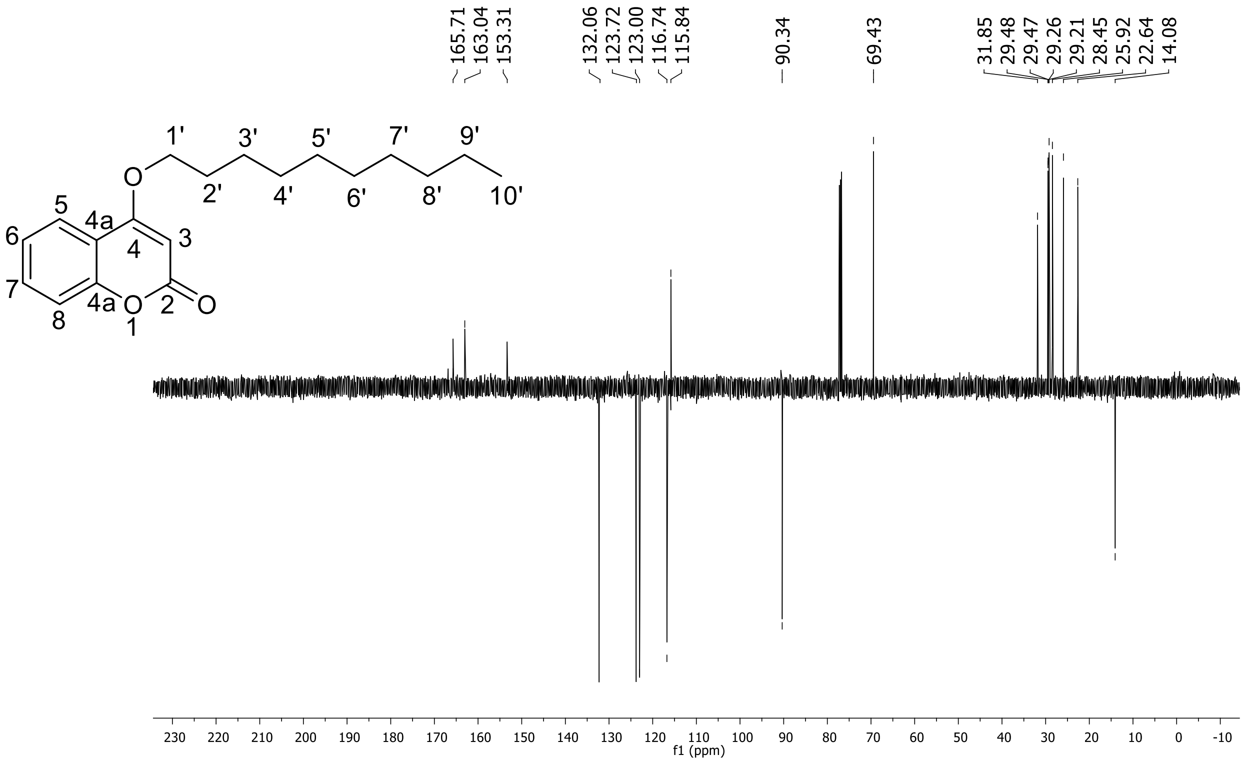

Sperctrum S3. ^{13}C NMR (125 MHz, $CDCl_3$) spectrum of compound **3.**

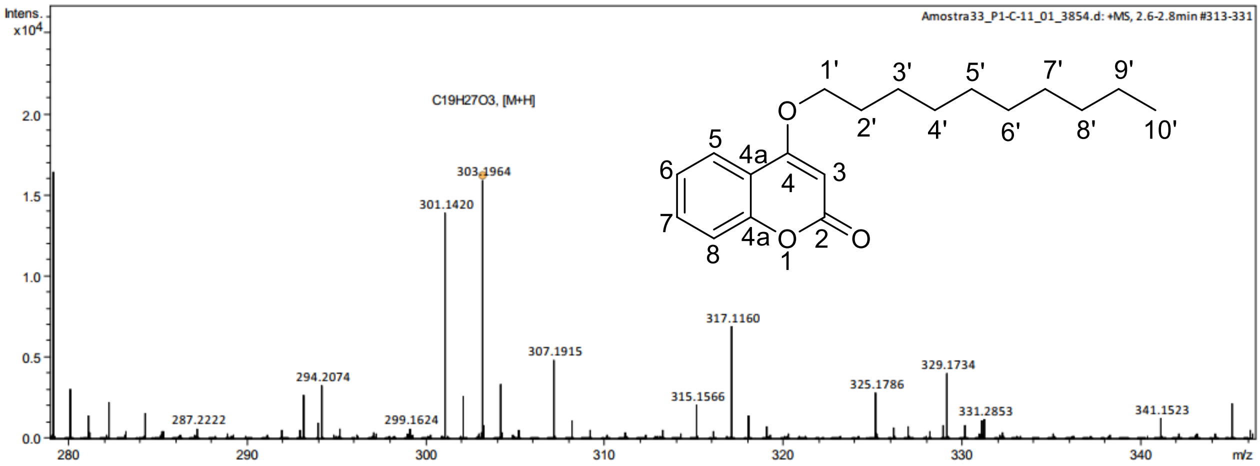

Spectrum S4. HRMS spectrum of compound **3.**

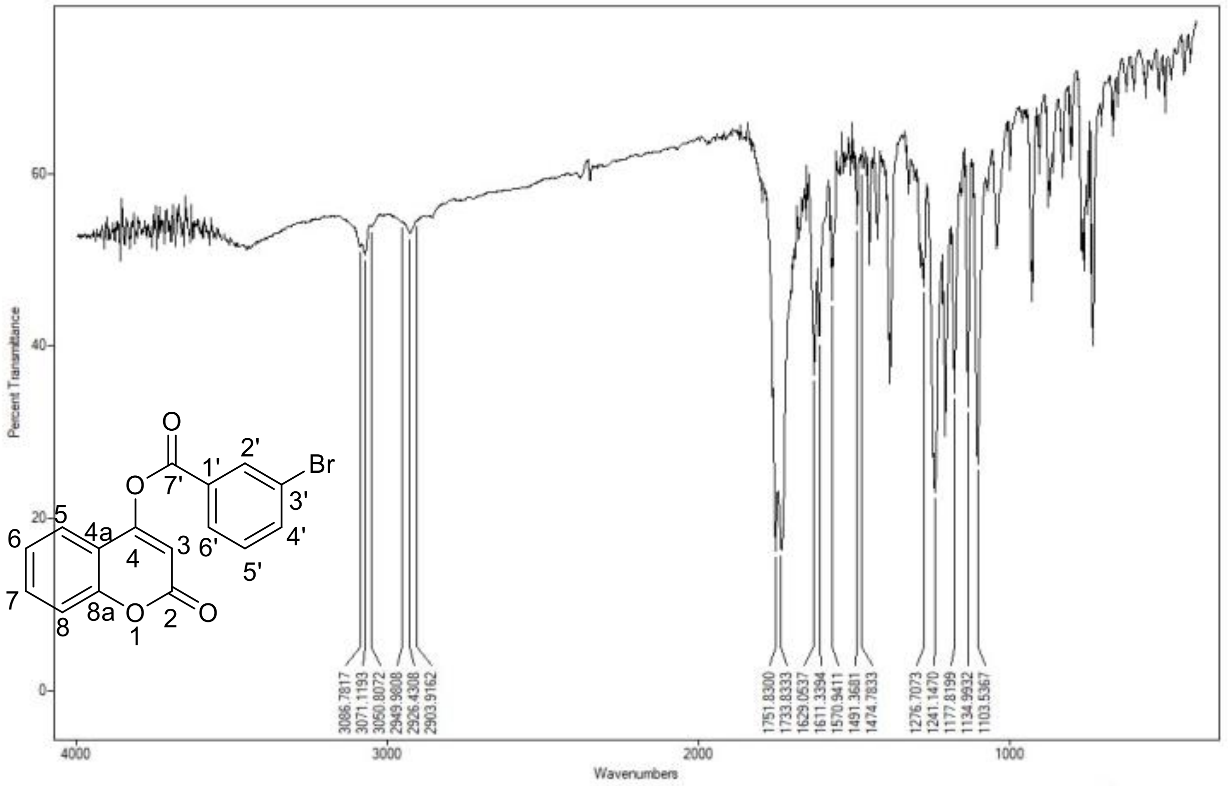

Spectrum S5. Infrared sprectrum υmax (KBr, cm-1) of compound **4.**

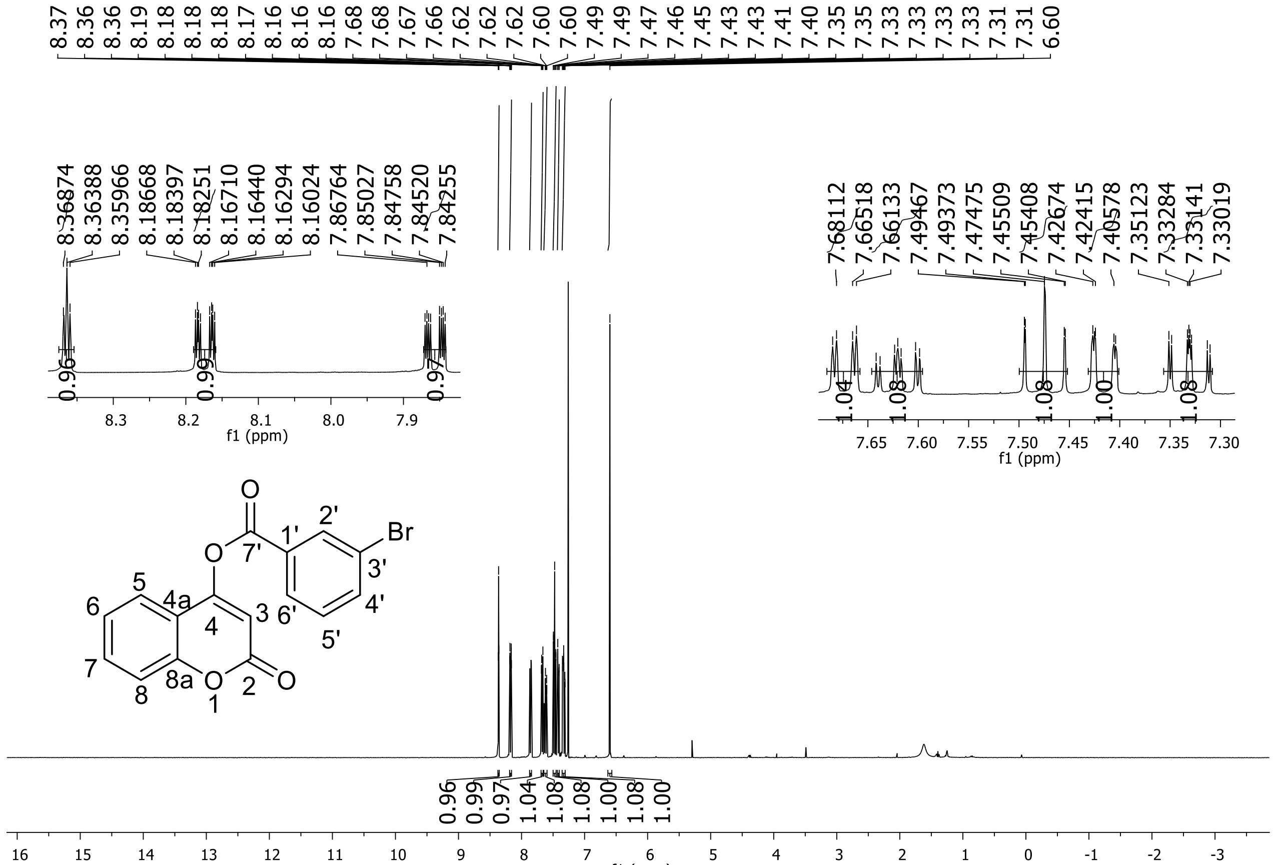

Spectrum S6. ^{1}H NMR spectrum (400 MHz, $CDCl_3$) of compound **4.**

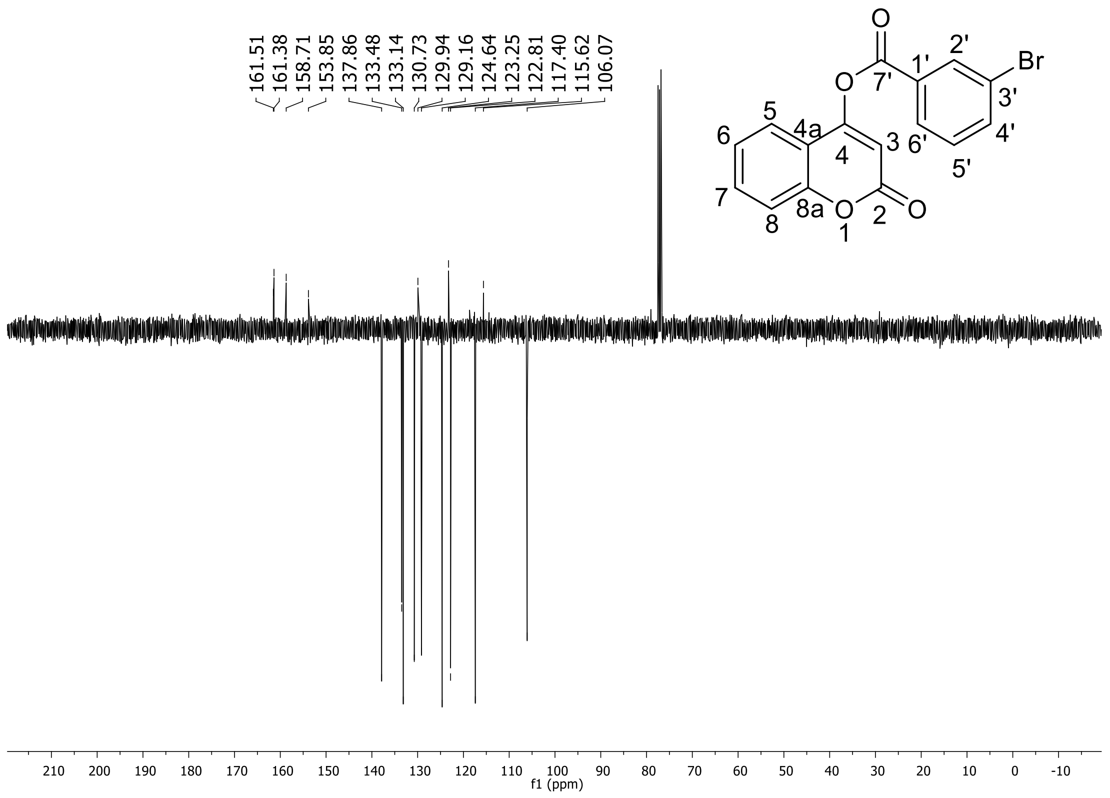

Spectrum S7. ^{13}C NMR spectrum (100 MHz, $CDCl_3$) of compound **4.**

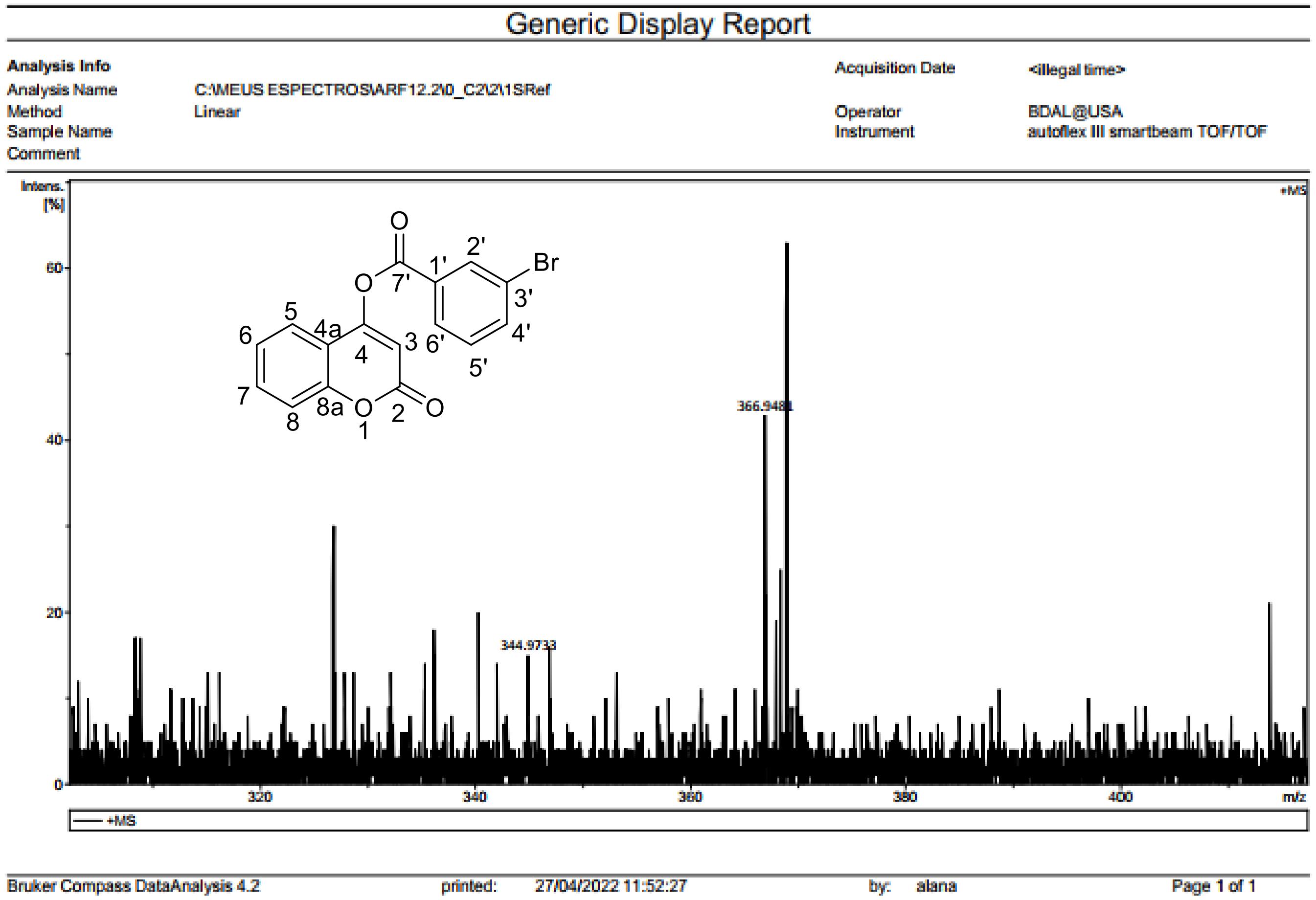

Spectrum S8. HRMS spectrum of compound **4.**

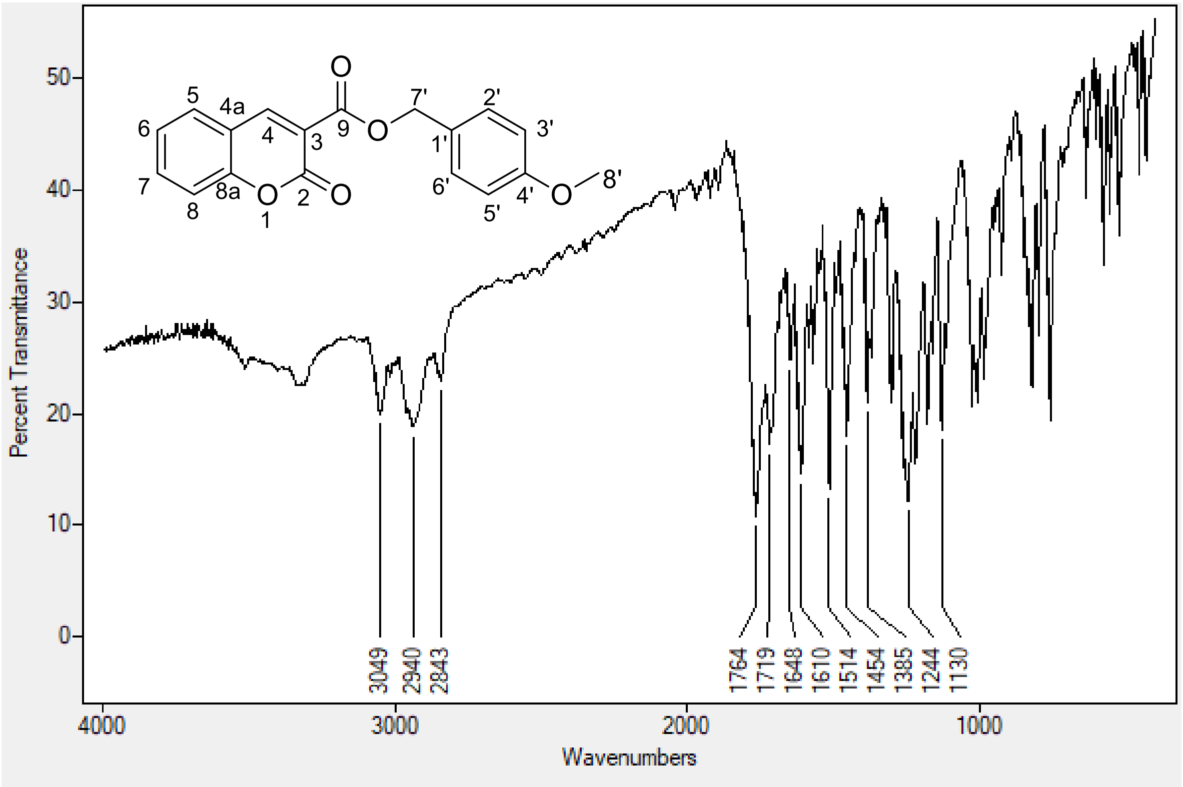

Spectrum S9. Infrared sprectrum υmax (KBr, cm-1) of compound **10 .**

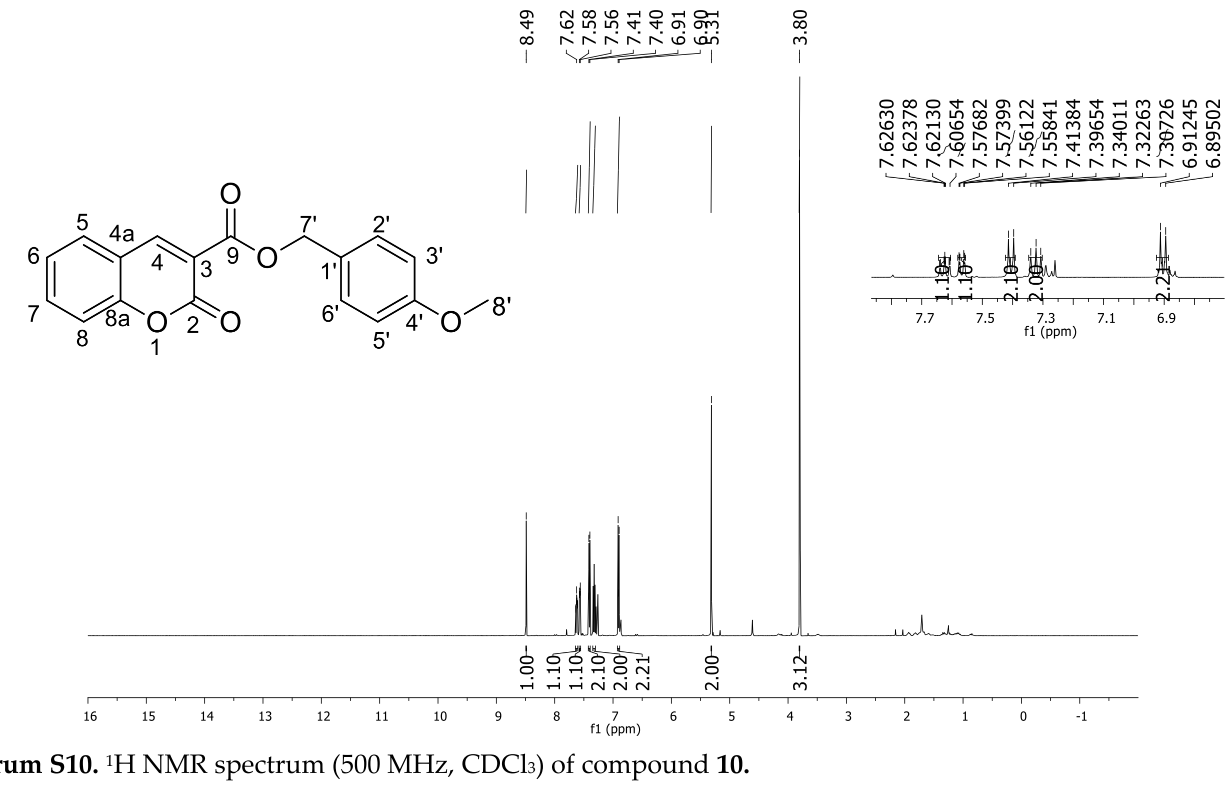

Sperctrum S10. ^{1}H NMR spectrum (500 MHz, $CDCl_3$) of compound **10.**

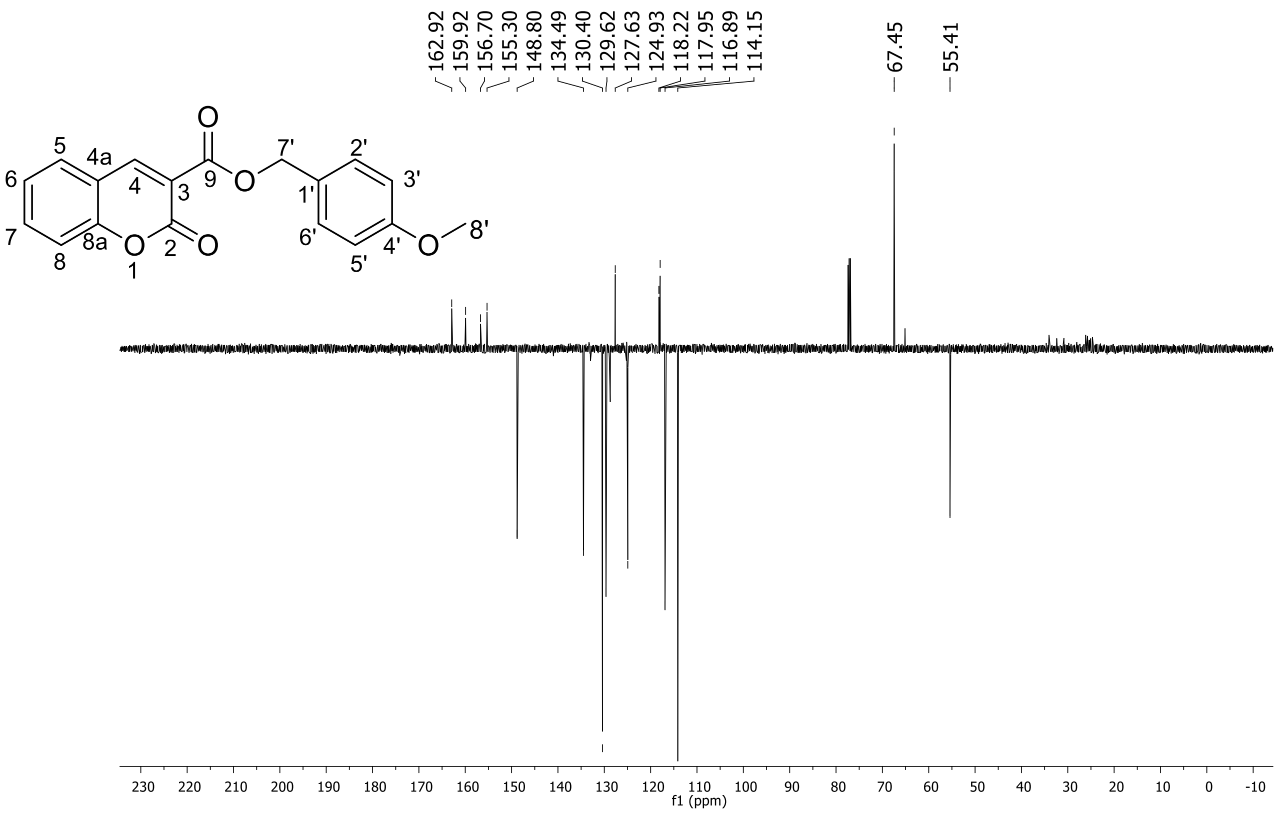

Spectrum S11. ^{13}C NMR spectrum (125 MHz, $CDCl_3$) of compound **10.**

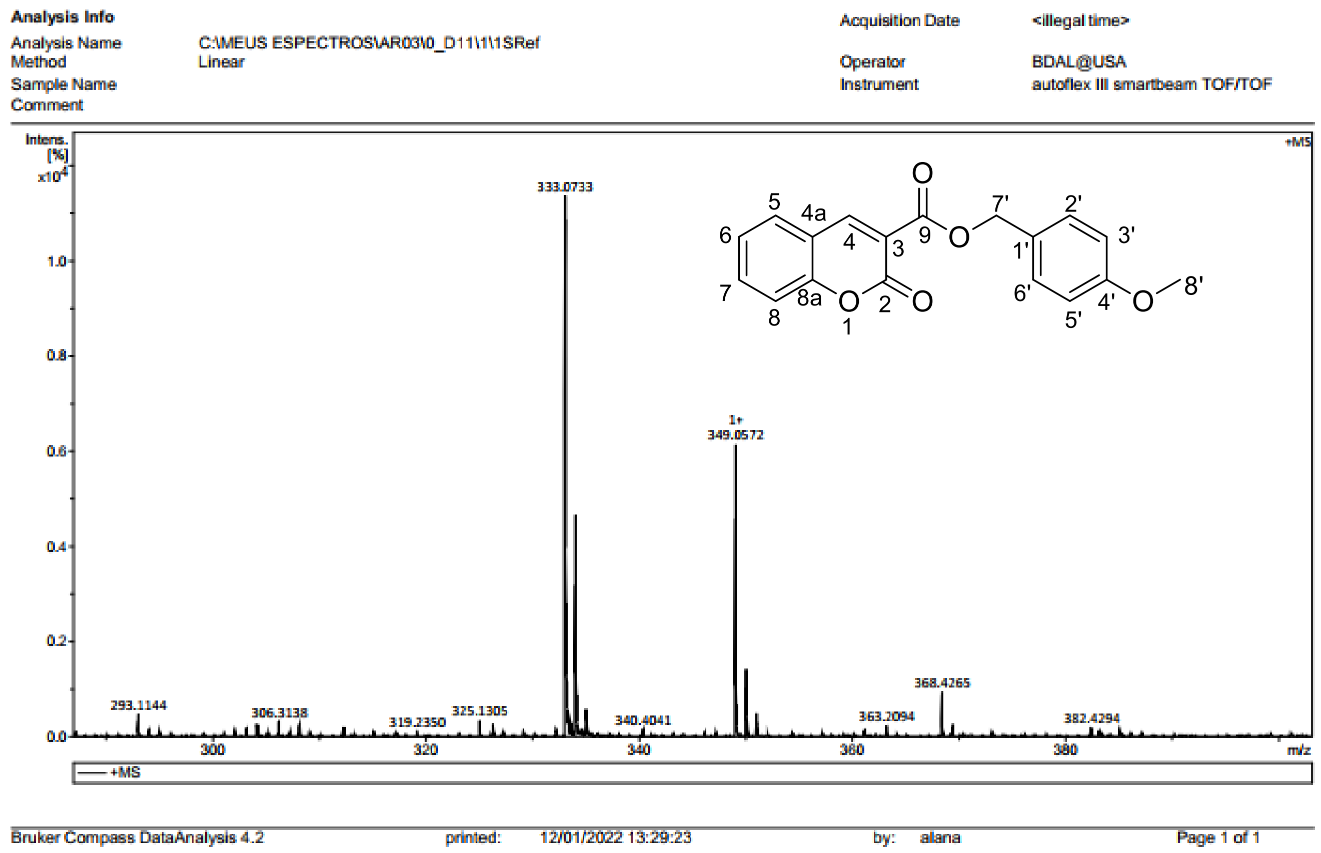

Spectrum S12. HRMS spectrum of compound **10.**

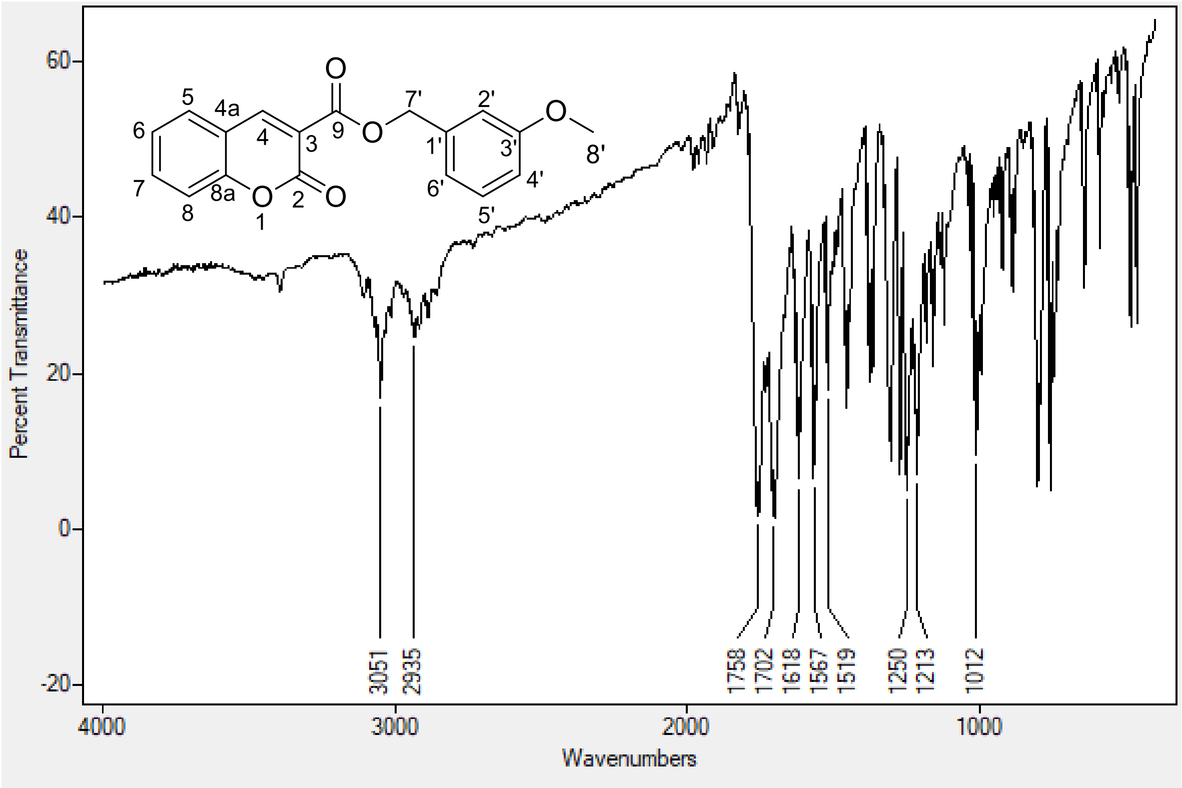

Spectrum S13. Infrared sprectra υmax (KBr, cm-1) of compound 11.

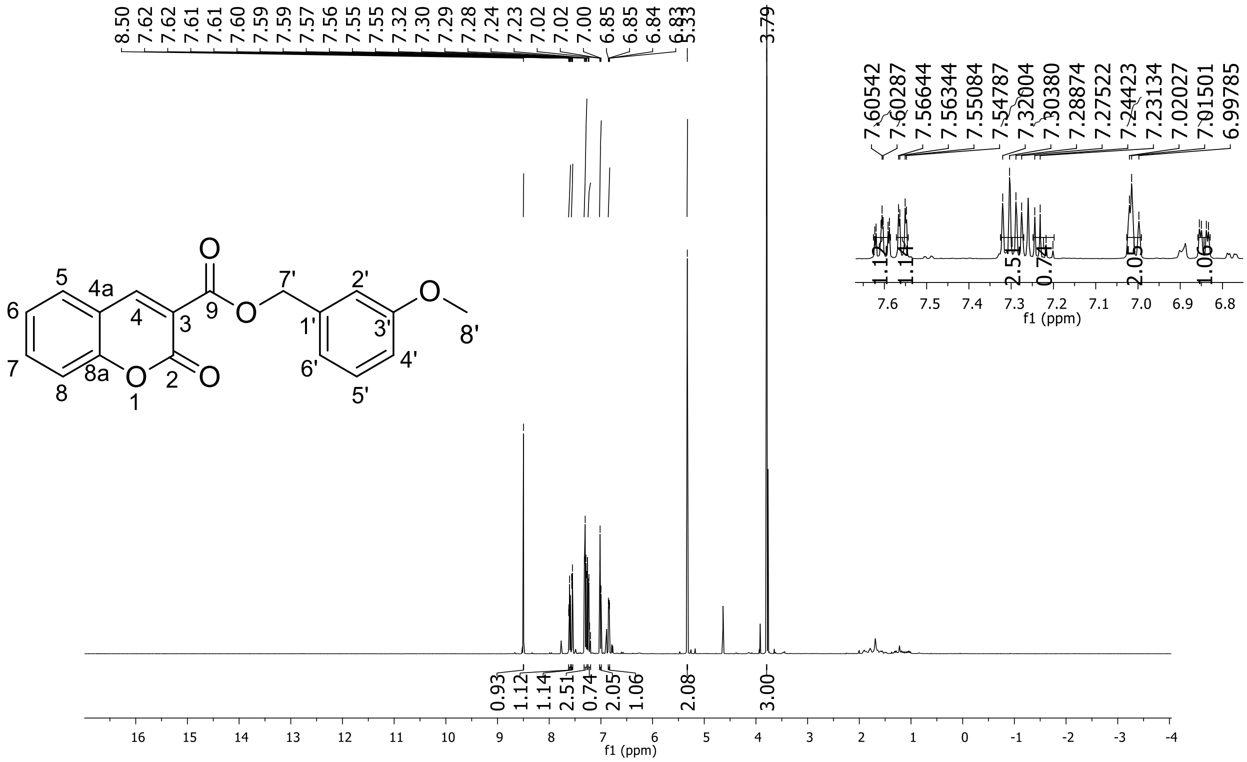

Spectrum S14. ¹H NMR spectrum (500 MHz, $CDCl_3$) of compound **11.**

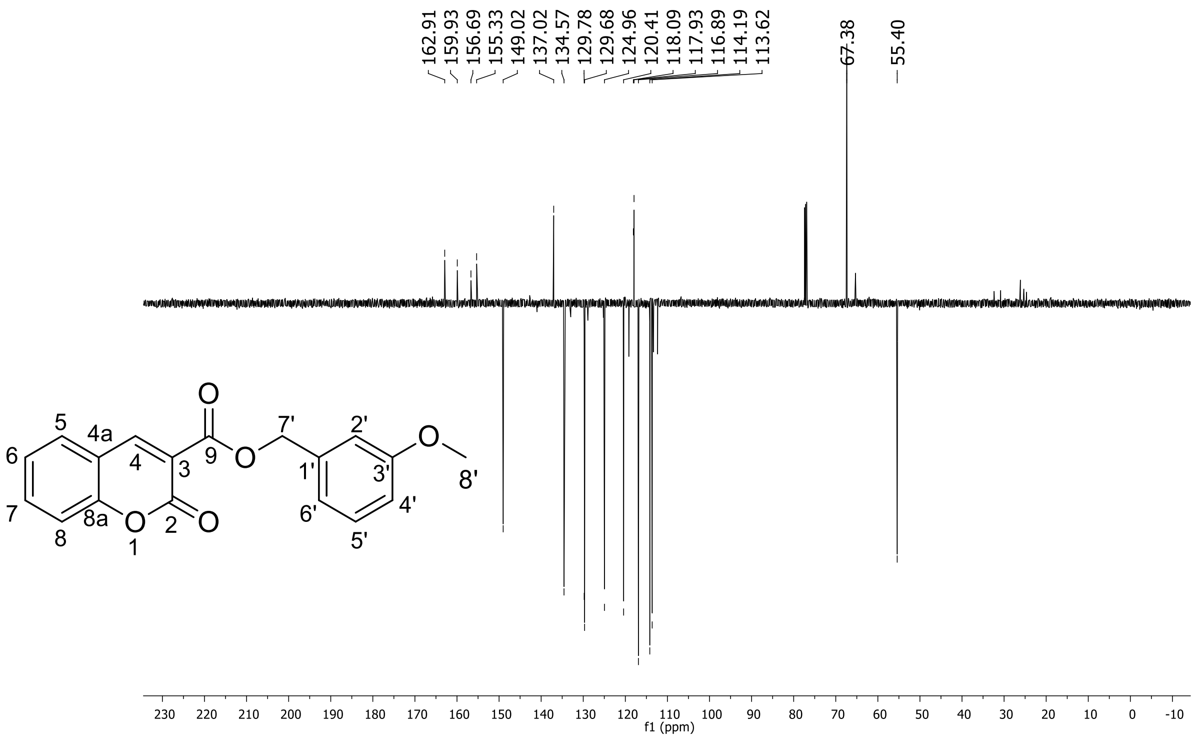

Spectrum S15. ^{13}C NMR spectrum (125 MHz, $CDCl_3$) of **11.**

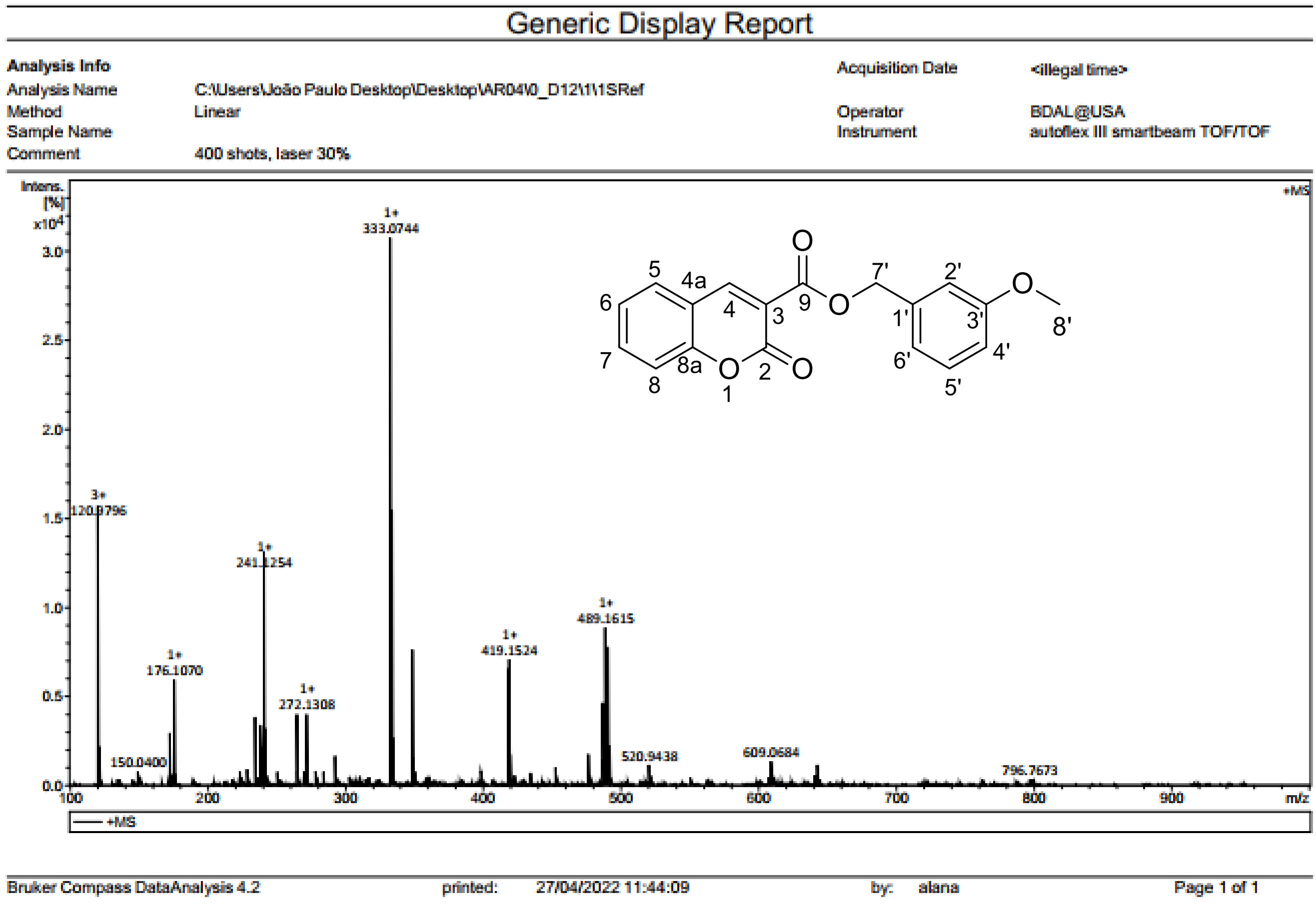

Spectrum S16. HRMS spectrum of compound **11.**

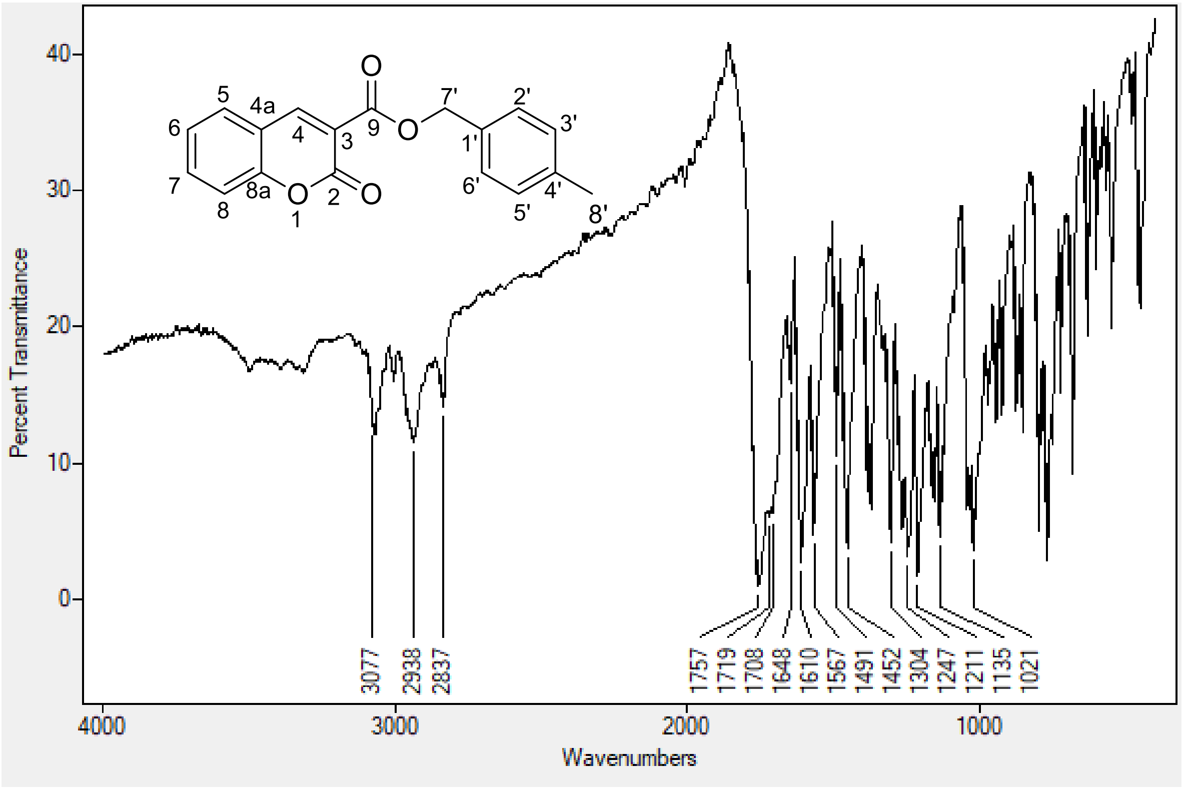

Spectrum S17. Infrared sprectrum ʋmax (KBr, cm-1) of compound **12.**

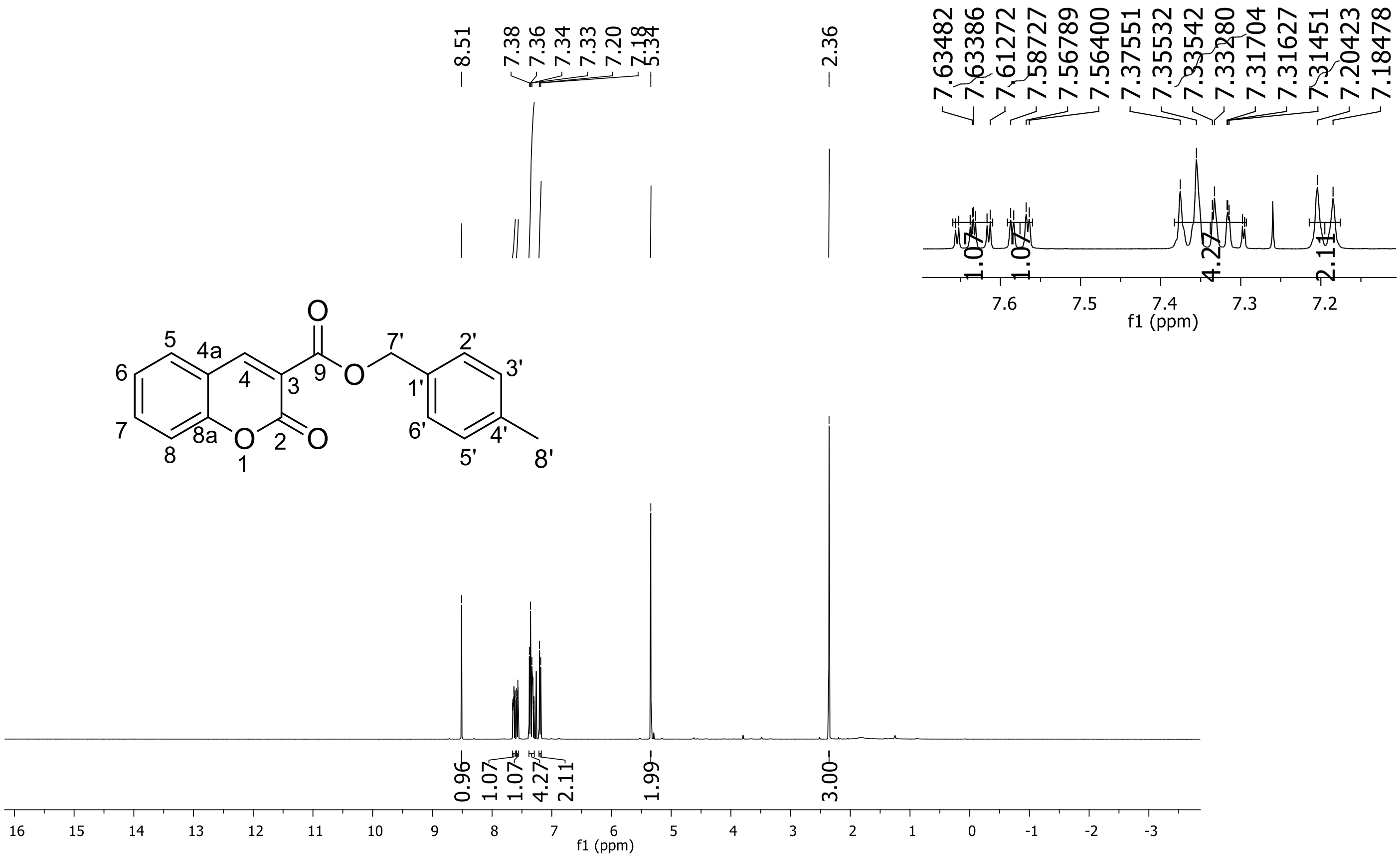

Spectrum S18. ^{1}H NMR spectra (400 MHz, $CDCl_3$) of compound **12.**

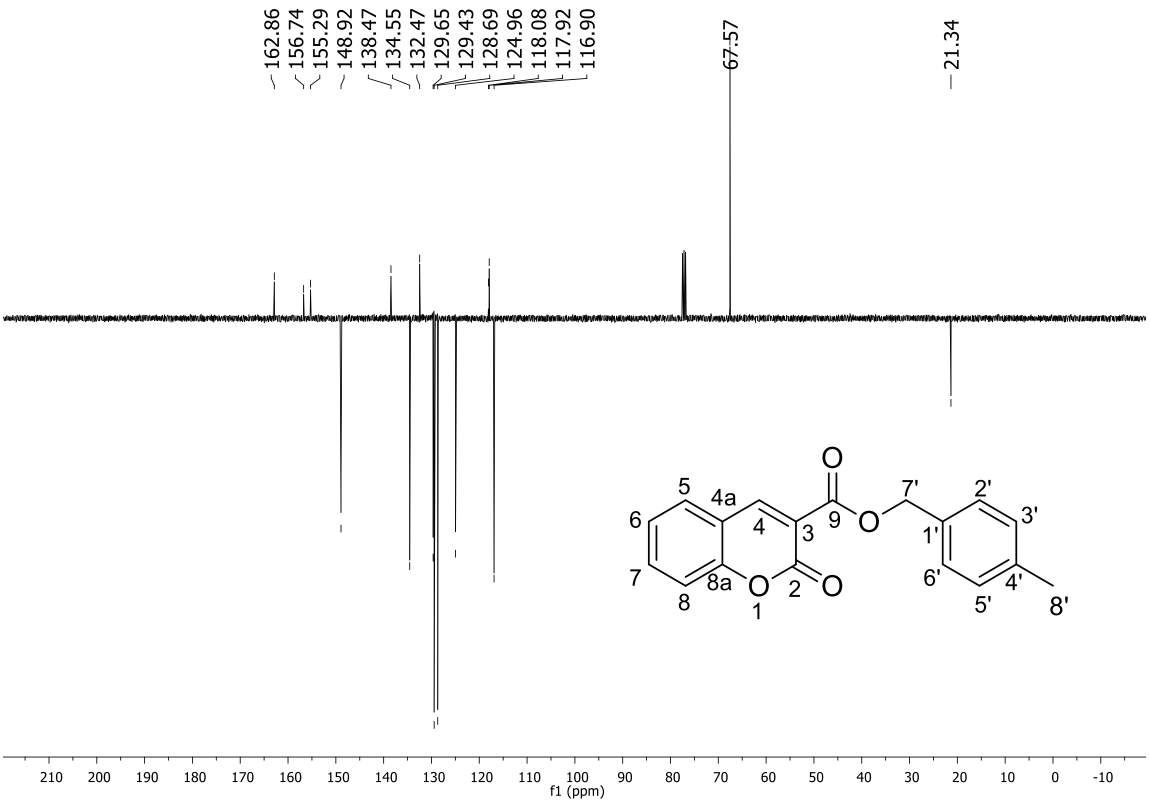

Spectrum S19. ^{13}C NMR spectrum (125 MHz, $CDCl_3$) of compound **12.**

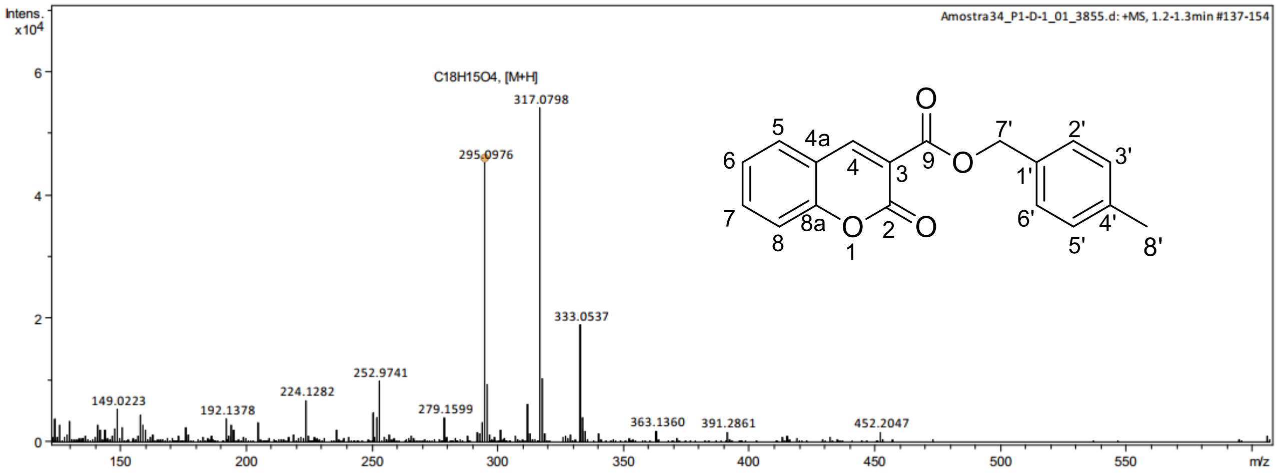

Spectrum S20. HRMS spectrum of compound **12.**

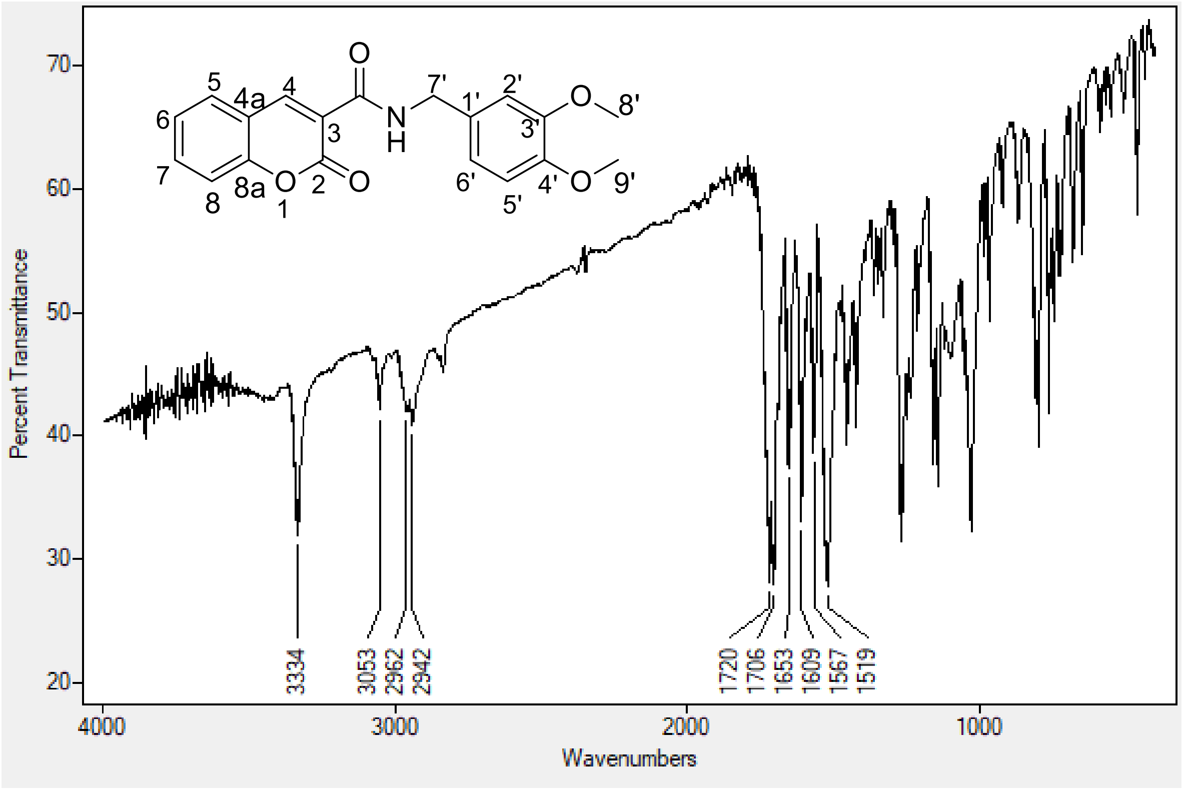

Spectrum S21. Infrared sprectrum υmax (KBr, cm^{-1}) of compound **16.**

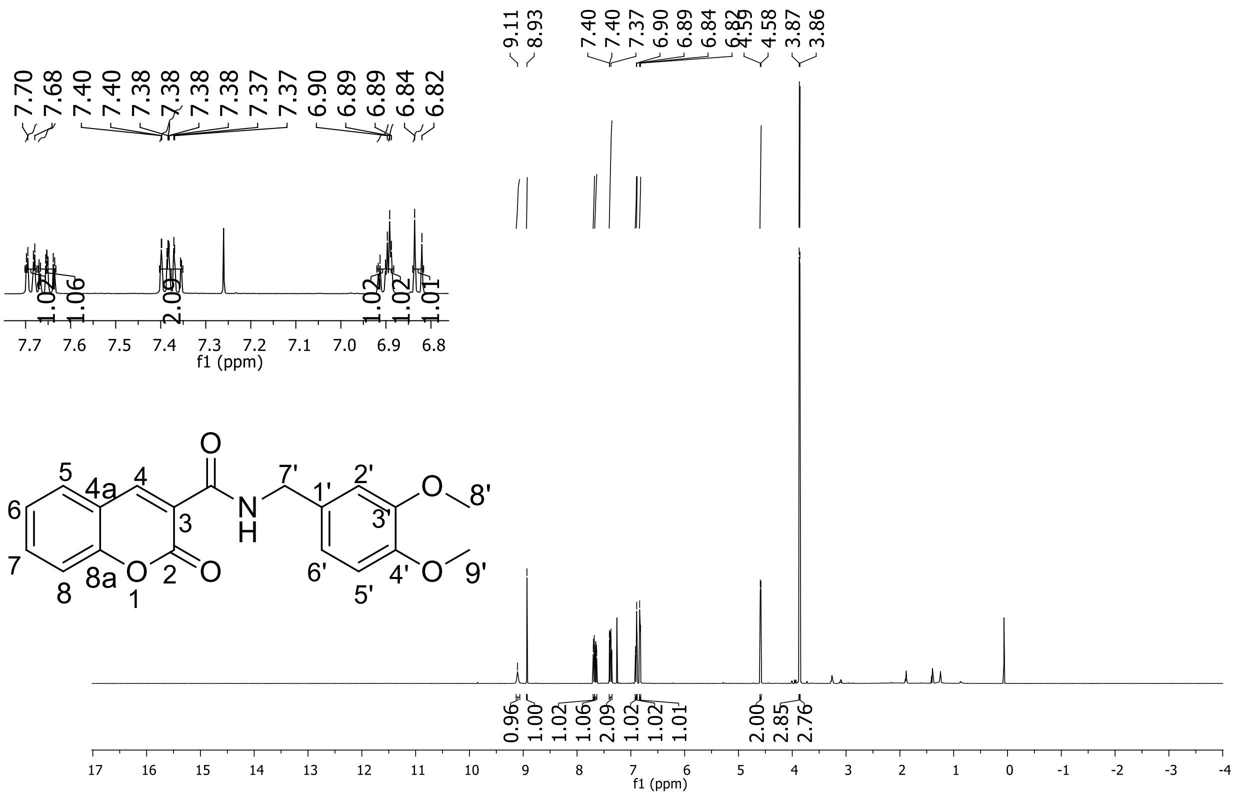

Spectrum S22. ^{1}H NMR spectrum (500 MHz, $CDCl_3$) of compound **16.**

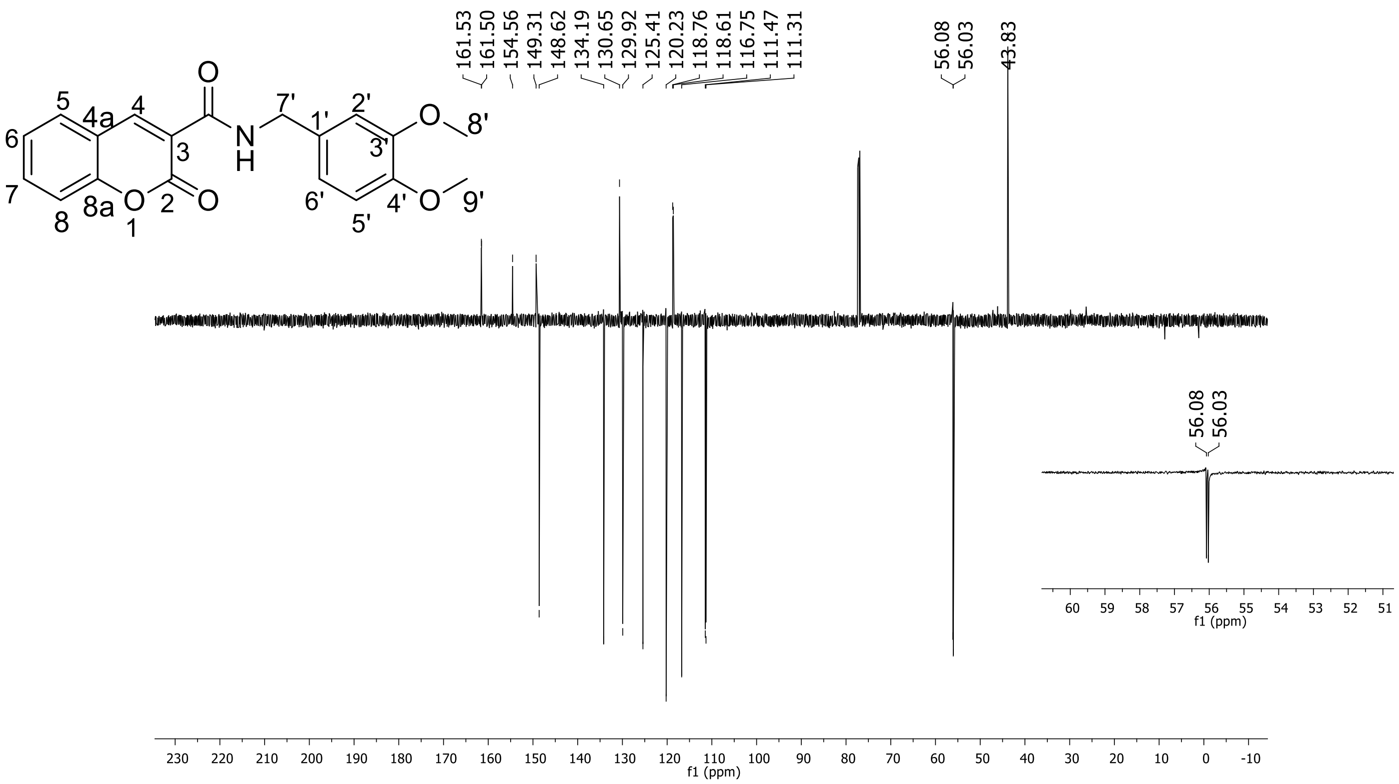

Spectrum S23. ^{13}C NMR spectrum (125 MHz, $CDCl_3$) of compound **16.**

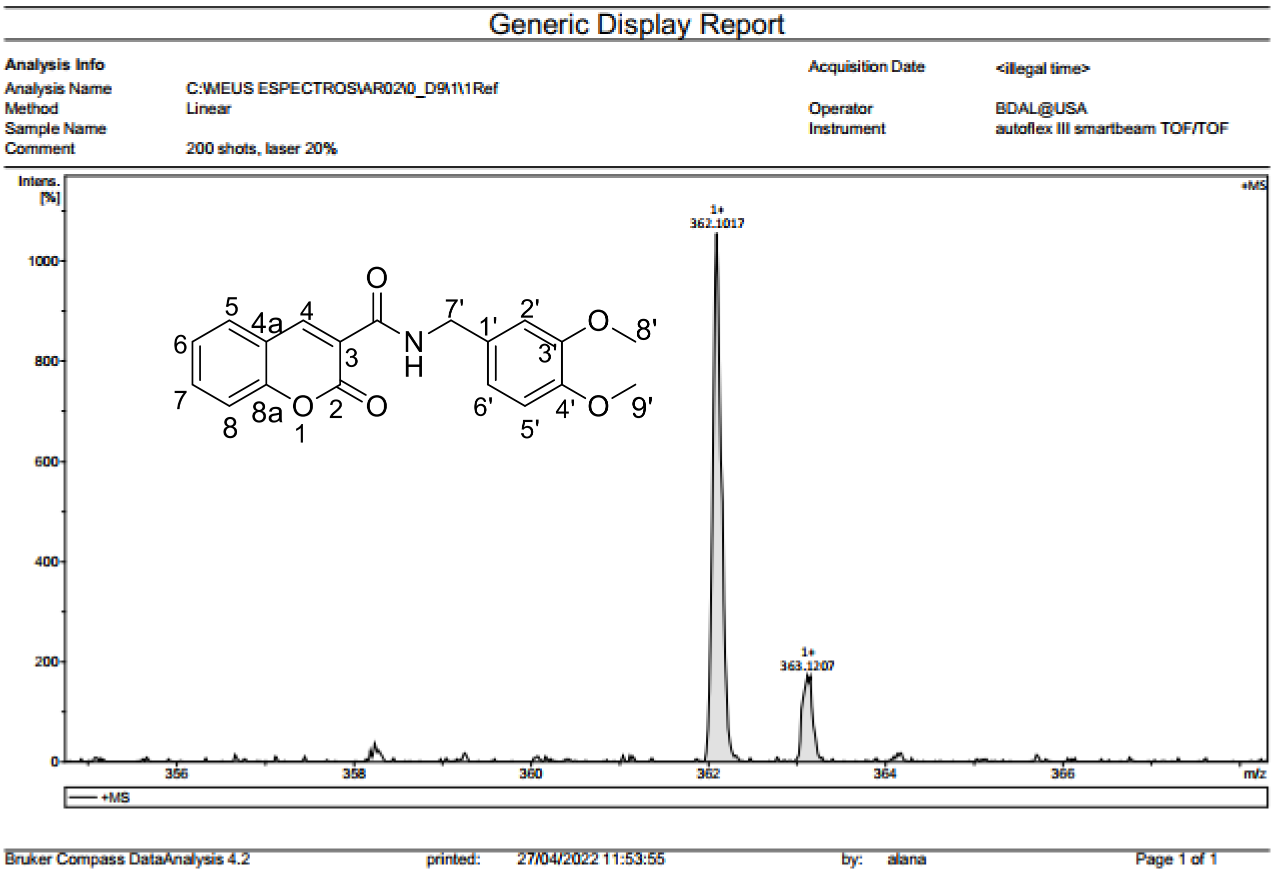

Spectrum S24. HRMS spectrum of compound **16.**
